# Can reflective multicriteria be the new paradigm for healthcare decision-making? The EVIDEM journey

**DOI:** 10.1186/s12962-018-0116-9

**Published:** 2018-11-09

**Authors:** Mireille M. Goetghebeur, Marjo S. Cellier

**Affiliations:** 1 School of Public Health, University of Montreal, 7101 Park Ave, Montreal, H3N QC Canada; 2 Montreal, QC Canada

**Keywords:** Ethics, Framework, Multicriteria decision analyses, Health technology assessment, Priority-setting, Legitimacy, Value, Equity, Sustainability, Relevance

## Abstract

**Background:**

Multiple technologies, procedures and programs call for fairly-based decisions for prioritization of healthcare interventions. There is a diversity of perspectives of what constitutes a legitimate decision, which depends on both the process and the reasoning applied. Current approaches focus on technical aspects while methods to support alignment of decisions with the compassionate impetus of healthcare systems is lacking.

**Methods:**

The framework was developed based on an analysis of the foundations of healthcare systems, the reasoning underlying decisions and fair processes. The concept of reflective multicriteria was created: it assumes that decisionmakers guided by a generic interpretative frame rooted in the compassionate impetus of healthcare systems, can sharpen their reasoning, raise awareness of their motivation and increase legitimacy of decisions. The initial framework was made available through a not for profit organization (the EVIDEM Collaboration, 2006–2017) to stimulate its development with thought leaders and stakeholders in an open source philosophy. Development was tailored to the real-life needs of decisionmakers and drew on several domains of knowledge including healthcare ethics, evidenced-based medicine, health economics, health technology assessment and multicriteria approaches.

**Results:**

The 10th edition framework builds on four dimensions: (1) the universal impetus of healthcare systems, (2) reasoning, values and ethics, (3) evidence and knowledge on interventions, and (4) a transformative process. Mathematical aspects of the framework are designed to help clarify, express and share individual reasoning; this non-conventional use of numbers requires a cultural change and needs to be phased in slowly. The framework includes four tools for easy adaptation and operationalization: (a) concepts and operationalization, (b) adapt and pilot, (c) evidence matrix, (d) mathematical representation of reasoning. Application is useful throughout all types of healthcare interventions, for all levels of decision, and across the globe.

**Conclusion:**

By clarifying their reasoning while keeping decisionmakers aware of the impetus of healthcare systems, reflective multicriteria provides an effective approach to increase the legitimacy of decisions. Beyond a tool, reflective multicriteria pioneered by EVIDEM is geared to transform our vision of the value of healthcare interventions and how they might contribute to relevant, equitable and sustainable healthcare systems.

**Electronic supplementary material:**

The online version of this article (10.1186/s12962-018-0116-9) contains supplementary material, which is available to authorized users.

## Background

Since the establishment of the first universal healthcare systems about 50 years ago, a number of domains of research and applications have emerged to operationalized the impetus which led to their creation. This compassionate impetus stemmed from providing care and alleviating suffering at the population level, thereby expanding the compassionate impetus which takes place at the individual level during a patient-healthcare practitioner encounter. Operationalization of such a noble cause meant providing the most relevant care at the individual and at the population level, and in a sustainable manner.

The exponential increase of healthcare technologies, procedures and programs resulted in many benefits but the concomitant steep increase in costs have challenged the foundation of healthcare systems, calling for fairly-based decisions and prioritization of healthcare interventions. The WHO [1] recently pointed out that countries ought to be accountable to the populations they serve and that they should establish processes for legitimate healthcare decisions and prioritization. Berwick has called for a deep reflection on the “triple aim” (patient, population, and sustainability) and how these often conflicting aims might be resolved when making choices [2].

Both evidenced-based medicine, developed to ensure best choices at individual level, and health economics, developed to inform allocation of resources at the population level, and the associated cost-effectiveness paradigm, have contributed to the quest for legitimacy. Health technology assessment (HTA), developed to ensure best choices in a given society, was an important step in this quest [3], with a key contribution of the EUnehTA core model [4]. However, these fields focused on technical aspects, while decisions are value-laden and based on a “complex reasoning which takes place amidst a diversity of perspectives of what constitutes a fair decision [5].

Healthcare ethics have built on theories established over the centuries to support legitimacy of decisions. Two decades ago, Daniels and Sabin [6] proposed four conditions for a fair process in their seminal Accountability for Reasonableness (A4R) framework. More recently, Clark and Weale [7] structured social values into procedural and substantive values, as two key aspects of legitimacy in healthcare decisions. Recently Daniels et al. called for an expansion of HTA [8]. However, approaches to support alignment of decisions with the compassionate impetus of healthcare systems is lacking.

Multicriteria decision analysis (MCDA) has emerged as a possible approach to further the quest for legitimacy in healthcare decisions [9, 10]. MCDA is traditionally seen as reductionist approach to a decision problem, which seems ill adapted to the quest for fair reasoning and legitimate decisions [11, 12]. Reflective multicriteria assumes that decisionmakers guided by a generic interpretative frame rooted in the compassionate impetus of healthcare systems, can sharpen their reasoning, raise awareness of their motivation and increase legitimacy of decisions. The objective of this paper is to provide an overview of this approach pioneered by EVIDEM.

## Methods

### Analysis of the foundations of healthcare systems, reasoning and fair processes

The framework was developed based on an analysis of the foundation of healthcare systems, the reasoning underlying decisions and fair processes. It is assumed that legitimacy of decisions depends on consideration of such foundations, on a fairly-based reasoning (individual level), and on a fairly-based process (institutional level).

The foundation of healthcare systems is the compassionate impetus to achieve health for all. This can be expressed into three general ethical imperatives (normative aspects): (1) prevent/alleviate suffering in individual patients with relevant healthcare interventions, (2) prioritize those who are worst off while providing the greatest benefit for the greatest number, and (3) ensuring long term sustainability of healthcare systems; combined with the wisdom to make decisions adapted to the context (feasibility aspect). These aspects include elements of key ethical theories and positions such as deontology, distributive justice, utilitarianism and virtue ethics (practical wisdom).

At the individual level, the reasoning underlying a decision includes: (1) the general motivation of the individual, (2) the reflection on the criteria considered and the evidence (scientific, colloquial, imputed by logic) for each criteria for the intervention appraised, and (3) the conclusion of this reflection which represents the balancing act of one’s individual perspective and interpretation in regard of the healthcare system impetus. A fairly-based reasoning presupposes some alignment of the individual motivation with the compassionate impetus of healthcare systems.

Institutional decisions require processes that support legitimacy. The A4R framework of Daniels and Sabin [6] proposes four conditions for a fair process with deliberation among representative stakeholders being essential. These conditions were recently re-stated by Daniels as follows: “publicity of rationales for choices; relevance of criteria agreed to by a broad range of stakeholders; revisability of the decision in light of new evidence or arguments; and enforcement that means the other conditions are met” [12]. The A4R has been criticized for lacking guidance for the relevance condition and for relying mainly on committee members’ values.

To support legitimate decisions, the framework therefore needs to derive criteria from the impetus of healthcare systems, to structure the reasoning in all its aspects and requires a process derived from the principles of A4R. The concept of reflective multicriteria was created based on this analyses: it assumes that decisionmakers, when guided through a fair process by a generic interpretative frame rooted in the compassionate impetus of healthcare systems, can sharpen their reasoning, raise awareness of their motivation and increase legitimacy of decisions. An initial framework was thus designed as part of a research grant [13].

### Reflections drawing on current domains of research and applications

The initial framework was then made available through a not for profit organization with free membership (the EVIDEM Collaboration) to stimulate its development for over 10 years (2006–2017). Development was tailored to the real-life needs of decisionmakers (bottom-up approach) and drew on several domains of knowledge including decision ethics, evidenced-based medicine, health economics, health technology assessment and multicriteria approaches. Below are examples of lines of reflection derived, through an open source philosophy, from academic and experiential knowledge of thought leaders and stakeholders for more than 40 countries who joined the membership.

#### Observation 1: Traditional ethical theories and positions, selection of criteria and ethical dilemmas

The framework needs to be rooted in the compassionate impetus of healthcare systems so its basis is acceptable from a universal standpoint; resolution of ethical dilemmas that arise more and more often is then guided by the fulfillment of this impetus.

Reflection: If we attempt to derived criteria to operationalize this impetus using a single traditional ethical theory in healthcare (e.g., utilitarianism), we will miss aspects of other traditional ethical theories (e.g., deontology, distributive justice, virtue ethics, procedural ethics), which are directly related to the compassionate impetus of healthcare systems and that are considered in real-life decisions to tackle ethical dilemmas
Approach selected: Criteria are derived from the compassionate impetus of healthcare systems in a generic manner, and thereby incorporate aspects of major traditional ethical theories; each criteria is justified by at least one aspect of a traditional ethical theory. Ethical dilemmas are tackled by reminding decisionmakers of the ethical aspect underlying each criteria, and whether the intervention (or group of interventions) considered contributes to more relevance, more equity, more sustainability and is adapted to the context (concept of maximum value).

#### Observation 2: Traditional health economics, cost and cost-effectiveness

For an application at the system level, the framework needs to prioritize (rank) interventions based on their fulfillment of the compassionate impetus of health care system (maximum value), including financial sustainability; current approaches prescribe cost and effectiveness as opposed concepts rather than contributing both to the value of an intervention.

Reflection: If we attempt to perform a value measurement based on all aspects except cost (which traditional health economics tend to promote), it will not address the sustainability imperative. Similarly, the concept of cost-effectiveness considers cost as an element distinct of the value of an intervention.
Approach selected: The value measurement integrate economic aspects (cost of intervention, associated cost or savings in medical and non medical resources); if possible, cost effectiveness is omitted as a criteria as it is a composite measure combining data from several generic criteria (efficacy, safety, cost of intervention, other types of cost, etc.); this combination does not facilitate interpretation of concepts, and tends to create double counting and confused reasoning.

#### Observation 3: Traditional HTA and real-life judgments

To facilitate sharing of reasoning among committee members and stakeholders, the framework needs to capture interpretation of evidence and associated inherent judgment that takes place on the spot in real-life deliberation.

Reflection: If, to assess the performance of interventions on each criteria, we use computed scoring scales as recommended in traditional MCDA (computed scales are based solely on the numerical data provided as evidence; e.g., 10 mm Hg reduction would be one of the scoring scale options for efficacy of antihypertensives), the framework will not address the fact that “interpretation of data that takes place during appraisals requires judgement” (Sir Rawlins, founder of NICE, at the Health Technology Assessment International plenary session in Seoul in 2013).
Approach selected: Interpretative scoring scales were designed to capture the interpretation of numbers (rather than transforming numbers into other numbers); this is a non-conventional use of numbers since in this case numbers represent a judgment. Thus, if, for a given criteria, it can be agreed by users what constitutes low and high end of such a scale, then this criterion may be considered quantitatively; if not, quantitative scoring should not be performed, and solely qualitative considerations should be reported in the multicriteria grid.

#### Observation 4: Traditional MCDA and the need for a comprehensive interpretive frame

The framework cannot be reductionist but it has to be pragmatic with regard to the number of criteria, and these criteria have to be legitimate in regards to the compassionate impetus of healthcare systems.

Reflection: If we attempt to have only a few criteria to keep the framework simple, it will not address the point that many criteria will anyway be considered during the deliberation leading to the decision. In addition, fulfilling the compassionate impetus of healthcare systems requires a comprehensive set of criteria encompassing the various aspects of legitimacy in a given context.
Approach selected: The high level value system derived from the compassionate impetus can be structured into a set of generic criteria (e.g., effectiveness of intervention in the generic sense rather than specific outcomes such as effect on blood pressure) to provide an overarching generic interpretative frame (i.e., independent from the specificities of healthcare interventions); such generic frame can however be further specified with sub-criteria (e.g., specific outcomes measures, specific characteristics of a device). The generic interpretive frame should not constrain reasoning but rather clarifies individual reasoning in the context of the compassionate impetus of healthcare systems. It needs to encompass a comprehensive set of criteria derived from this impetus. Difficulties in operationalization of a criteria (e.g., lack of evidence, complexity) should not be a reason for not including it in a generic framework. Indeed, it is likely that it will be considered anyway during the deliberation and a comprehensive set of criteria is key in legitimate decisions. Finally, criteria need to be defined bearing in mind the multicriteria principles of non-redundancy, independence, operationalizability and completeness.

#### Observation 5: Traditional weights and representation of individual value system

Traditional weight elicitation techniques in MCDA are geared to elicit preferences of individuals, independently of the compassionate impetus of healthcare systems. Individuals making a decision with the compassionate impetus of healthcare systems in mind need to identify their individual value systems in this regard. This value system is therefore related to this impetus rather than to the specificities of the intervention assessed. Weighting should therefore be one independently of the interventions being assessed.

Reflection: If we use traditional approaches focusing on preferences, we will not develop a reflection of what can be done collectively to develop interventions that are contributing to the compassionate impetus of healthcare systems.
Approach selected: To raise awareness of the compassionate impetus of healthcare systems, the framework elicits, through direct weighting methods, the value system of an individual (or of a group) rather than preferences. The weights are thus independent of a given healthcare intervention. In this non-conventional approach, the trade-off between the criteria is the trade-off between the underlying ethical imperative of each criteria (e.g. equity). When aware of the ethical imperatives underlying each criteria, direct weighting techniques become values elicitation technique stimulating deeper reflection on individual value systems (non-conventional use of weighting techniques) Weighting therefore becomes a key element to the ‘reflective” approach, which only direct weighting methods can support (indirect weighting methods are not geared to stimulate reflection).

Over the years, numerous lines of reflection have been made using this methodological frame of reflection, discussing with researchers and users in the organic manner that open source philosophy relies on.

## Results

The framework is designed to support fair reasoning and deliberation to increase legitimacy of decisions. This is done by guiding decisionmakers through a fair process, using a generic interpretive frame rooted in the compassionate impetus of healthcare systems. Such frame, by providing a common road map is geared to facilitate communication across policy committee members, patients and physicians, and healthcare stakeholders at large.

The framework builds on four dimensions described below: (i) Universal impetus of healthcare systems, (ii) Reasoning, values and ethics, (iii) Evidence and knowledge on interventions, and (iv) Transformative process over time. It includes four tools for easy adaptation and operationalization: (a) concepts and operationalization (Additional file 1), (b) Adapt and pilot [step by step manual, Additional file 2), (c) Evidence matrix (step by step manual, Additional file 3), (d) Mathematical representation of reasoning (Excel calculator, Additional file 4).

### EVIDEM 10th edition

**Fig. 1:**
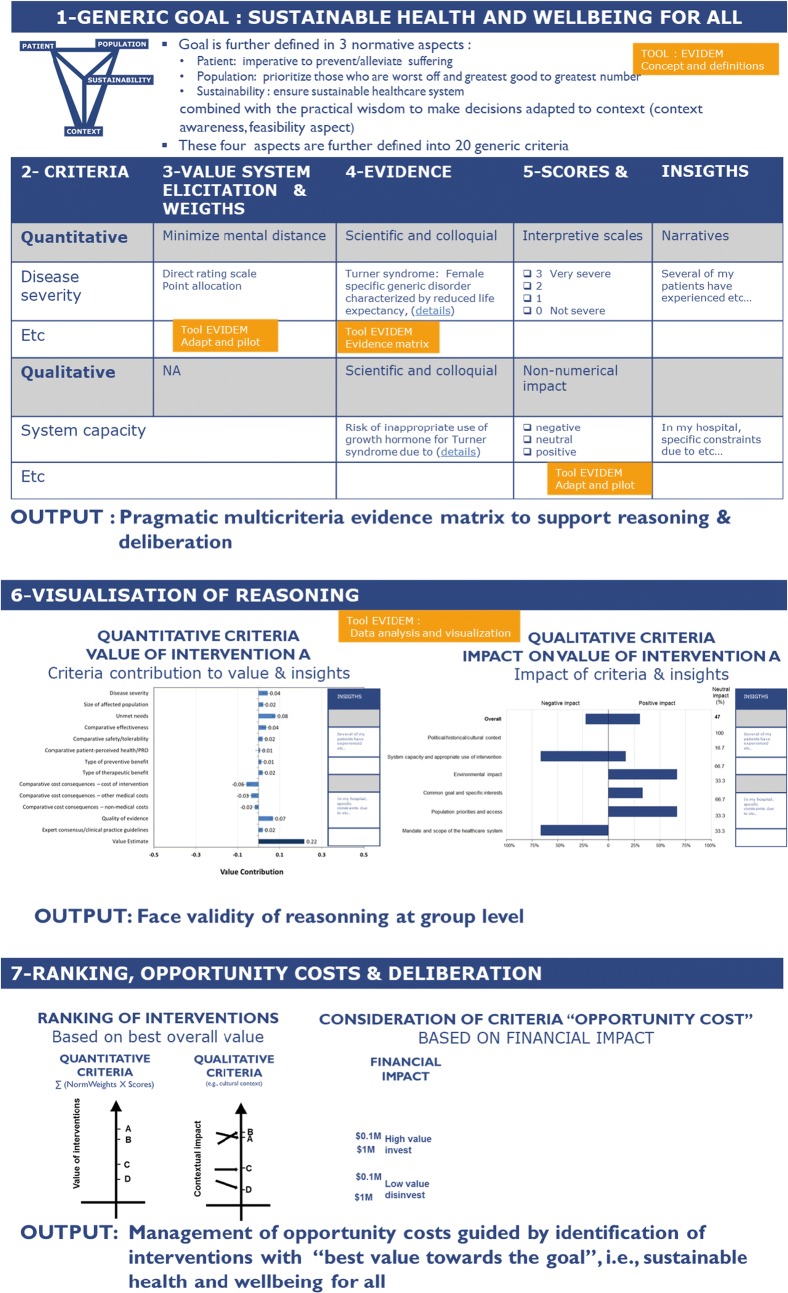
Visual representation of EVIDEM 10th edition

The 10th edition is based on 10 years of open source development. Although EVIDEM does use some features of MCDA, its roots are not in this methodology but rather in real-life deliberation and decision (bottom-up approach). However, to facilitate its understanding, its visual representation reflects the standard steps in MCDA (Thokala) [14] [(1) goal, (2) criteria, (3) weights, (4) evidence, (5) scores, (6) visualization and uncertainty, (7) ranking and deliberation] (Fig. 1).

#### Universal impetus of healthcare systems

To ensure that the universal compassionate impetus of healthcare systems (i.e., achieve health for all) remains at the core of decisions, the framework includes generic criteria directly derived from this impetus. “Generic” is used in the sense of being applicable to all interventions, types of decision and region. Four universal aspects related to this impetus and 20 criteria, derived from informal and formal consultation and research [15, 16] are defined including (see part 1 and 2 of Fig. 1):

- Three ethical imperatives (normative aspects):
  1. Alleviate/prevent suffering of patients (5 criteria: efficacy, safety, patient perspective, type of preventive benefit, type of therapeutic benefit).
  2. Prioritize those who are worst off while ensuring greatest good for greatest number (4 criteria: disease severity, unmet needs, size of population, country population priorities).
  3. Ensure sustainability (6 criteria: cost of intervention; other medical costs; non-medical costs; opportunity costs for the system; scope of healthcare system; environmental impact).
- Contextual imperatives (feasibility aspect), defined as the wisdom of making decisions informed by knowledge and adapted to context (5 criteria: quality of evidence; expert consensus; system capacity; specific interests; political, historical and cultural context).

This set of generic criteria creates a generic interpretive frame which, by design, is a reminder of the compassionate impetus of healthcare systems. This should be borne in mind when adapting the framework by removing/adding generic criteria. Limiting the number of generic criteria for methodological reasons may constrain the reasoning and compromise the integrity of the comprehensive interpretive frame on which EVIDEM is built. Of note, for each generic criterion, several sub-criteria are proposed to reflect specificities of therapeutic areas or types of interventions. Details are available in the EVIDEM tool *Concepts and definitions* which provides a rationale on each aspect of the framework and a guidance for adaptation (Additional file 1).

#### Reasoning, values and ethics

Criteria are organized in an operationalizable framework, designed to structure and clarify individual reasoning in all its aspects:

1. The general motivation of the individual: by stating the compassionate impetus clearly, the framework raises awareness on one’s individual motivation and its alignment with this impetus
2. The individual reflection on:
  - the generic criteria considered: what matters most to me according to my own value system? what is the relative importance of criteria?; This can be elicited informally or formally through a value system elicitation method (weighting) combined with a narrative and face validity exercise to confirm that the weights reflect the value system of the individual (for details, see previous research [17] and options proposed in the Tool Adapt and pilot, Additional file 2).
  - the evidence considered: what is the evidence available (scientific, colloquial, imputed by logic, my own insights) for each criterion for the intervention appraised? Evidence is made available for each criterion using instructions described in the Tool Evidence matrix, Additional file 3); how the intervention performs on each of these criteria? This can be elicited informally or formally through an interpretive scoring scale capturing judgment (see Tool Adapt and pilot, Additional file 2).
3. The conclusion of this individual reflection: the balancing act of these considerations is facilitated by the interpretive grid, which serves as a reminder us of the underlying ethical imperative associated with each criterion [18].

As illustrated in Fig. 1, the framework allows to elicit individual value systems (part 3), aligns criterion with associated evidence which give a visual cartography of available knowledge (part 4). This structure clarifies reasoning and allows to express this reasoning through interpretative scoring scales and or insights (part 5).

#### Evidence and knowledge on interventions

The way evidence and knowledge on interventions is conveyed to the decisionmaker has a critical impact on the decision. Therefore, detailed instructions based on good HTA practices [19] adapted to multicriteria approaches to research, synthesize and report evidence for each criterion are provided in the tool Evidence matrix (Additional file 3). These instructions aim at synthesising and presenting best available and most relevant evidence (scientific, colloquial, imputed by logic) for each criterion in a clear format. These instructions also ensure that the reflection is as unobstructed as possible by irrelevant or biased data. The tool also includes instruments to assess quality of evidence (clinical, patient reported outcomes, economic, epidemiologic), initially derived from a number of quality assessment tools [13] and updated over the years.

#### A transformative process

The framework is designed to promote a fair process by stimulating reflection and deliberation, fostering transparency and clarity, ensure accountability and relevance of decisions while facilitating communication, participation and collaboration as well as appeal and revision. It is geared to generates a transformation of current processes into processes that are more aligned with the principles of the A4R.

Individual reasoning made explicit through the interpretive grid of the framework becomes shareable into a group deliberation; each aspect of the grid is enriched by insights and reflection from other members of the group, transforming the individual perspective into a rich exchange though a deliberation leading to an equilibrium on which the group decision is based. Decisions made through such a process, with a constant reminder of the compassionate impetus, in a committee composed of members that represent the diverse perspective at stake, provides a good basis for legitimate decisions.

The framework implementation is meant to be phased in carefully depending on the culture of the institution and the region in which it is to be established (. e.g., perception on transparency, positioning towards diversity of perspectives). Of note, although mathematical aspects of the framework are designed to help clarify, express and share individual reasoning, this non-conventional use of numbers requires a cultural change and needs to be phased in slowly.

In a first step, the framework can be applied in a qualitative mode that uses the interpretive frame (multicriteria grid) to capture individual interpretations for each criterion in a narrative form (see Additional file 2). This supports the group deliberation, using implicit weights and scores to arrive at a decision. This piloting allows to experience the process, reveals to users its pros (efficiency gains, clarity, ease of use) and cons (changes required in current process), and initiates a reflection and transformation of activities surrounding decision processes.

A mixed qualitative/quantitative approach can be phased in if there is interest to further the transformation. When using the quantitative aspects of the framework, it is crucial to bear in mind that they are meant to help visualize and share the reasoning. They involve technical aspects such as weight and score elicitation and their aggregation, and face validity needs to be done at each step to ensure that the mathematical transformation truly reflects the reasoning (see tool Data analysis and visualisation of reasoning, Additional file 4). Such mathematical transformation allows to rank interventions. Ranking is modulated by qualitative considerations. Over time, this supports the transformation of the basket of healthcare services towards interventions well adapted to the context and contributing to more relevance, more equity and more sustainability of healthcare systems (concept of maximum value). The mathematical aspects are thus designed to help clarify, express and share the reasoning, not as a substitute of it; they increase the power of transformation towards healthcare systems geared to achieve the compassionate impetus on which they were created. For details see the important notice available in each EVIDEM tool (Additional files 1, 2, 3 and 4).

### A reminder of the motivation: name and logo

**Fig. 2:**
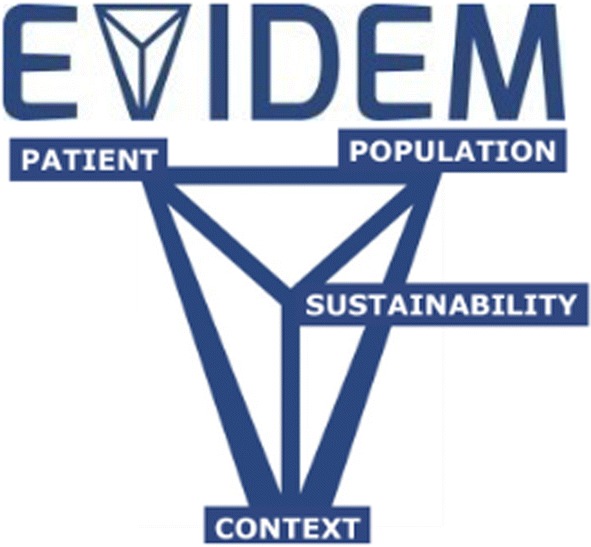
The EVIDEM logo—the stylized V represents an equilibrium across the three ethical imperatives (patient, population, sustainability) derived from the compassionate impetus of healthcare systems

The name EVIDEM (Evidence and Values Impact on DEcision Making) reflect its objective: support decision making, and the associated reasoning which requires consideration of evidence and values. The stylized V of the Logo (Fig. 2) represents the three basic ethical imperatives underlying decisions for healthcare systems: relevance at the patient level, equity at the population level and sustainability (top of V), which need to be based on a good understanding of the Context (bottom of V, contextual imperative) in which healthcare interventions are to be used. It serves as a reminder of the compassionate impetus of healthcare systems and the aspects to be kept in mind when making a decision. This “reminder” aspect is at the heart of EVIDEM.

### Applications

Application is useful throughout all types of healthcare interventions, for all levels of decision, and across the globe. By design, the framework facilitates interpretations of any decision-making situation, both at the individual and a t the group level. This resulted in a variety of applications including coverage decisions (its initial object), but also for benefit-risk evaluations, shared decision-making, clinical research question prioritization, design of clinical trials. Applications are ongoing in several countries, most of these are not public domain. A few applications of EVIDEM have been published [18, 20–27].

For example, in Italy, the Lombardy Health Directorate developed a reflective multicriteria approach combined with the EUnetHTA core model to appraise health technologies; this has been in place to make coverage decisions since 2012 [28]. The reasoning of the committee members is made available openly on a web site in a format combining visual representations of the decision rationale and narratives. A key output is the facilitation of the interactions with stakeholders as a result of a transparent process. The process is evolving to further increase legitimacy by involving a wider array of stakeholders such as citizens or patients.

In Colombia, the Ministry of Health and Social Protection, carried out a consultation to adapt EVIDEM criteria to the Colombian context, as part of its exploration of methodologies for coverage decisions. In this pilot, criteria weights were elicited through participation of more than 200 stakeholders including experts and citizens [29]. A pilot testing of the adapted framework revealed that a reflective multicriteria approach for complemented by a budgeting exercise was well adapted to assess thoroughly healthcare interventions. It helped to structure and clarify reasoning and deliberation of committee members. Further developments are ongoing to implement participative approaches rooted in A4R enhanced by multicriteria approaches.

Recently, the EVIDEM framework was adapted to build the list of priority devices for cancer care published in 2017 by the WHO. It was used as a mean to collect experiential knowledge from a diversity of experts around the world and to support the reflection leading to the inclusion/exclusion from the list of priority devices. The generic tools developed for this application were also designed to be directly usable, with some contextual adaptation, by members states to make fair decisions regarding medical devices [30].

In 2017, to celebrate its 10 years of success and to facilitate its widespread use, the EVIDEM Collaboration resolved to make freely available the 10th edition of the EVIDEM Framework, with no legal binding. This represented the dissolution of the legal entity, the not-for-for profit EVIDEM Collaboration. This important step led to the transformation of the network of active members into an open international community who shares an interest in reflective multicriteria approaches.

## Discussion

**Fig. 3:**
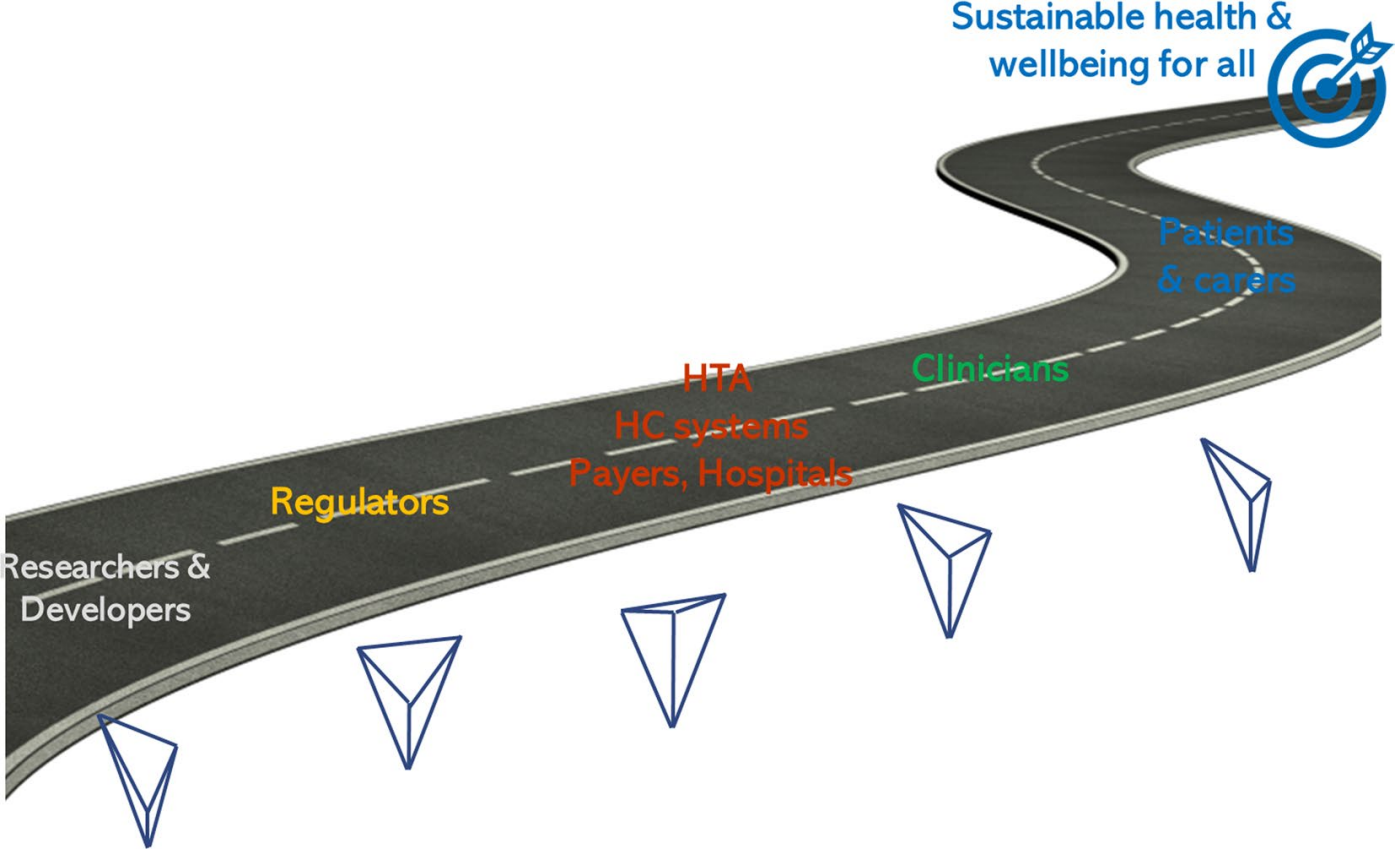
A common road map with the compassionate impetus of healthcare systems to transcend the diversity of perspectives; the variation in the stylized V of the EVIDEM logo represent the diversity of emphasis on the ethical imperatives derived from the compassionate impetus (patient, population, sustainability)

As mentioned by the Canadian Minister of Health Jane Philpott, Parliament Sept. 29, 2016, Canada and societies all over the world “need to find ways to put healthcare on the road to long-term sustainability”. EVIDEM is proposed as a common generic road map to collectively achieve health for all (Fig. 3).

One can wonder what would happen if all levels of decisions were reminded to reflect on the compassionate impetus of healthcare systems and implement decision frameworks that remind them of it in their very design? If researchers and developers had in mind the constraints of HC systems to make their decision to develop? Regulators thinking not only of their remit but also encompassing the concept of sustainability that policymakers must consider? What would be our healthcare systems if all levels of decision had a clear idea of what patients need and how clinicians can best help them prevent and alleviate their suffering? Collectively, we can transform or establish more meaningful healthcare systems, with a vision that reflective multicriteria can help clarify in a diversity of contexts.

A paradigm change is mandatory to adapt healthcare systems to the twenty first century. Reflective multicriteria rooted in the objectives of universal healthcare systems opens the door to a deeper reflection and proposes a fair process to increase legitimacy, accountability and reasonableness of decisions [31]. Reasonableness stems from a balance of obvious and subtle elements, which comes from experiential knowledge of decision processes. Many aspects need to be considered to create the conditions of successful implementation and political feasibility of such approaches; one of them is piloting and learning from successes achieved so far in several regions of the world. Another is to communicate clearly that current approaches are not sustainable, that healthcare systems are approaching a perfect storm, and that change is mandatory.

## Conclusion

By clarifying their reasoning while keeping decisionmakers aware of the compassionate impetus of healthcare systems, reflective multicriteria provides an effective approach to increase the legitimacy of decisions at all the levels. Beyond a tool, reflective multicriteria pioneered by EVIDEM is geared to transform our vision of the value of healthcare interventions and how they might contribute to relevant, equitable and sustainable healthcare systems. It is hoped that reflective multicriteria will continue contributing to the movement “en marche” which is transforming current approaches focused on technical aspects into holistic approaches rooted in the ethical frames underpinning social desirability [32]. To paraphrase André Malraux, a French philosopher of the twentieth century: “Healthcare systems of the twenty first century will be based on decision processes aligned with their original compassionate impetus, or they will not be”.

## Additional files

**Additional file 1.** Concepts and definitions with detailed rationale on each aspect of the framework and guidance for their adaptation and implementation.

**Additional file 2.** Adapt and pilot. A step by step directly applicable manual to adapt and pilot the framework in context.

**Additional file 3.** Evidence matrix, Detailed instructions based on HTA principles to research, synthesize and report evidence for each criterion; instruments to assess quality of evidence.

**Additional file 4.** Data analysis and presentation; Excel calculator to facilitate compilation of data and narratives, perform calculation for quantitative approaches and combine them in visual representation to clarify reasoning.
