# Supplementary material for: Can reflective multicriteria be the new paradigm for healthcare decision-making? The EVIDEM journey
Source: Cost Eff Resour Alloc. 2018 Nov 9;16(Suppl 1):54. doi: 10.1186/s12962-018-0116-9 (PMC6225552; doi:10.1186/s12962-018-0116-9)
Supplement: Supplementary file 1 — Additional file 1. Concepts and definitions with detailed rationale on each aspect of the framework and guidance for their adaptation and implementation. [file 12962_2018_116_MOESM1_ESM.docx]

**Additional file 1**

**10th Edition of EVIDEM: Concept & definitions**

**Table of contents**

[1 Visualisation of evidem concept 6](#_Toc474782938)

[2 CRITERIA Conceptualization 7](#_Toc474782939)

[2.1 Normative universal criteria –core model 7](#_Toc474782940)

[2.2 Contextual criteria – CONTEXTUAL TOOL 8](#_Toc474782941)

[2.2.1 Normative contextual criteria 8](#_Toc474782942)

[2.2.2 Feasibility contextual criteria 8](#_Toc474782943)

[3 Criteria details: definition, scoring instructions and rationale 9](#_Toc474782944)

[3.1 Criteria of the MCDA CORE MODEL 9](#_Toc474782945)

[**Disease severity** 10](#_Toc474782946)

[**Size of affected population** 11](#_Toc474782947)

[**Unmet needs** 12](#_Toc474782948)

[**Comparative effectiveness** 13](#_Toc474782949)

[**Comparative safety / tolerability** 14](#_Toc474782950)

[**Comparative patient-perceived health / patient-reported outcomes** 15](#_Toc474782951)

[**Type of preventive benefit** 16](#_Toc474782952)

[**Type of therapeutic benefit** 17](#_Toc474782953)

[**Comparative cost consequences – cost of intervention** 18](#_Toc474782954)

[**Comparative cost consequences – other medical costs** 19](#_Toc474782955)

[**Comparative cost consequences – non-medical costs** 20](#_Toc474782956)

[**Quality of evidence** 22](#_Toc474782957)

[**Expert consensus / clinical practice guidelines** 23](#_Toc474782958)

[3.2 Criteria of the CONTEXTUAL TOOL 24](#_Toc474782959)

[**Mandate and scope of healthcare system** 24](#_Toc474782960)

[**Population priorities and access** 25](#_Toc474782961)

[**Common goal and specific interests** 26](#_Toc474782962)

[**Environmental impact** 26](#_Toc474782963)

[**System capacity and appropriate use of intervention** 27](#_Toc474782964)

[**Political, historical & cultural context** 28](#_Toc474782965)

[3.3 Consideration of opportunity costs 29](#_Toc474782966)

[**Opportunity costs and affordability** 29](#_Toc474782967)

**IMPORTANT NOTICE - SUMMARY**

**Please read before using EVIDEM**

EVIDEM is a reflective multicriteria approach designed to support the culture of reasonable decision-making by promoting procedural and substantive legitimacy; this includes selection of representative decisionmakers, relevance of reasons for the decision, publicity, appeal, and implementation – based on the ethical framework of accountability of reasonableness (A4R).

To help insure that decisions are based on relevant reasons (substantive legitimacy), EVIDEM provides a set of generic decision criteria derived from the ethical imperatives that underlie the common goal of healthcare and its ultimate motivation: compassion. This represents a generic interpretive frame (MCDA reflective grid) that can be used to elicit individual values and facilitate sharing of diverse perspectives during committee deliberations or for other applications (e.g., patient-clinician shared decisionmaking). These generic criteria can be further concretized to reflect specificities of therapeutic areas or types of interventions. EVIDEM also provides a common structure for all members to express their interpretation of the evidence for each criterion and thereby share their reasoning with others. These interpretations can be expressed quantitatively through interpretive scores (quantitative criteria), qualitatively through impacts (qualitative criteria) as well as narratively through comments (all criteria).

To pursue its objectives, EVIDEM was designed to reduce constraints of the natural decision and deliberation process by ensuring that: all relevant generic criteria are included (whether they are considered qualitatively or quantitatively); scientific and colloquial evidence relevant to each criterion is made available through an efficient synthesis methodology; and face validity is checked at each step of the process (weights, scores and corresponding narratives, aggregated measures) to ensure that visual representations of quantitative outputs reflect the reasoning of individuals or, for system level decisions (e.g., for HTA or MoH), of the committee within and across assessments.

**IMPORTANT NOTICE - DETAILS**

**Please read before using EVIDEM**

Although EVIDEM does use some features of MCDA, its roots are not in the methodology itself but rather in the natural decision and deliberation process. Its goal is to stimulate reflection, deliberation and reasonable decisions rather than algorithms approaches that traditional MCDA tends to promote. Each aspect of its design is geared to support the natural thinking process. It is built to minimize constraints of the natural reasoning in decisionmaking by providing a generic interpretive frame that can be shared across policy committee members, patients and physicians, and healthcare stakeholders at large.

**CRITERIA**

Criteria are selected to support the substantive legitimacy of the decision with regard to the common goal of healthcare systems. The goal is articulated in three normative aspects (ethical imperatives): 1 - alleviate/prevent suffering of patients; 2 – prioritize those who are worst off while ensuring greatest good for greatest number; and 3- ensure sustainability. This is combined with the wisdom of making decisions informed by knowledge and adapted to context (feasibility aspect). These aspects are expressed in 20 criteria in agreement with MCDA methodological principles of non-redundancy, independence, operationalizability and completeness. This creates a generic interpretive frame which, by design, is a reminder of the common goal of healthcare and its ultimate motivation.

THIS SHOULD BE BORNE IN MIND WHEN ADAPATING THE FRAMEWORK BY REMOVING/ADDING GENERIC CRITERIA

*Note 1: Attempts to limit the number of generic criteria for methodological reasons may constrain the reasoning and compromise the integrity of the comprehensive interpretive frame on which EVIDEM is built.*

*Note 2: For each generic criterion, a number of subcriteria are proposed in EVIDEM, which can be broken down further and integrated to reflect specificities of therapeutic areas or types of interventions.*

**REASONING LEADING TO A DECISION**

In the interpretive frame, the narratives and insights of the natural reasoning are structured by criteria and are complemented by qualitative and possibly quantitative outputs and visualization to facilitate their sharing and support the deliberation process.

***Evidence for Healthcare interventions***

When evaluating specific interventions, the goal of evidence synthesis and presentation (scientific and colloquial) is to provide for each criterion the best available and most relevant evidence in a clear format and ensure that the reflection is as unobstructed as possible by irrelevant or biased data.

***Qualitative approach*** The framework can be applied in a qualitative mode that uses the interpretive frame (MCDA grid) to capture interpretations of the available evidence for each criterion in a narrative form and uses implicit weights to arrive at a decision. A q*ualitative approach is recommended until a culture of the non-conventional use of numbers that EVIDEM proposes is well established in users.*

WHEN USING THE QUANTITATIVE ASPECTS OF THE FRAMEWORK BEAR IN MIND THET THEY ARE MEANT TO HELP VISUALIZE AND SHARE THE REASONNING

***Mixed-Qualitative - Quantitative approach:***

*Qualitative considerations*

Since some criteria are not suitable for scoring (e.g., cultural and historical context) but nonetheless are an integral part of the reasoning, the framework provides a simple qualitative assessment tool to consider the impact of these criteria (positive, neutral or negative) on the value of interventions.

*Quantitative considerations involve weight and score elicitation and their aggregation.*

*Value system elicitation (Weights) for generic criteria*: Weighting of generic criteria is approached in EVIDEM as a way to explore the value systems (values) of individuals. Since its objective is to stimulate reflection on what matters most to each individual, direct (rather than indirect) weight elicitation methods are proposed, combined with a narrative and face validity exercise to confirm that the weights reflect the value system of the individual

*Preferences (Weights) for specific subcriteria*: subcriteria that are specific to a therapeutic area or type of intervention can be elicited within a generic criterion (e.g., growth hormone: efficacy/effectiveness subcriteria: height [outcome 1], metabolism [outcome 2] etc.); the weights assigned to these represent individual preferences.

*Scores*: Reflective multicriteria analysis encourages the user to reflect on the evidence and make a judgment on its meaning using an interpretive scoring scale and also to provide a narrative to explain the reasoning that underlies the score. (Scores are thus a quantitative representation of an interpretation of the evidence, not a mathematical transformation of data.) These narratives can be summarized for each criterion at the group level for committees’ deliberations. Face validity of the visual representation of the scores is essential to ensure that the scores reflect the reasoning.

*Weights and scores aggregation*: Simple linear aggregation models are applied to create as little mental distance as possible between the measurement and the reasoning. To check face validity, users are presented with a visual representation of the aggregated measurement along with the contribution of each criterion and the associated narratives.

*Modulation by qualitative criteria*: the impact of each qualitative criteria on the aggregated measurement is considered. Face validity is checked at the group level, with a visual representation and associated narratives.

*Last criterion for consideration*:

After the evaluation based on all the other criteria is completed, the criterion “Opportunity cost and financial feasibility” is considered through a budgeting exercise, the committee performs a final deliberation on all aspects brought up and the decision is made.

*Ranking*

As the committee performs multiple assessments, face validity checks are carried out to ensure that the ranking based on the modulated aggregated measures does reflect the group reasoning within and across assessments. Adaptation of the framework is carried out as applicable over time.

THE MATHEMATICAL ASPECTS ARE THUS DESIGNED TO HELP EXPRESS AND SHARE THE REASONING

# Visualisation of the evidem concept

**In a first step, the framework is adapted to the decisionmaking context and values to reflect both normative (what should we do in this context? *i.e.*, with defined mandate and priorities) and feasibility aspects (what can be done?): criteria are selected accordingly. Once adapted, multicriteria evidence matrices are generated for each intervention and can be used as such for a qualitative approach.**

**For a mixed qualitative-quantitative approach, some qualitative criteria may be operationalized into quantitative criteria (redesign required); value system elicitation (weighting) method is selected, scoring scales are adjusted as applicable. For appraisal, weights, scores and insights are provided by evaluators, input is visualized, ranking is performed and can be used to guide management of opportunity costs. (see details in section below and in other EVIDEM tools).**


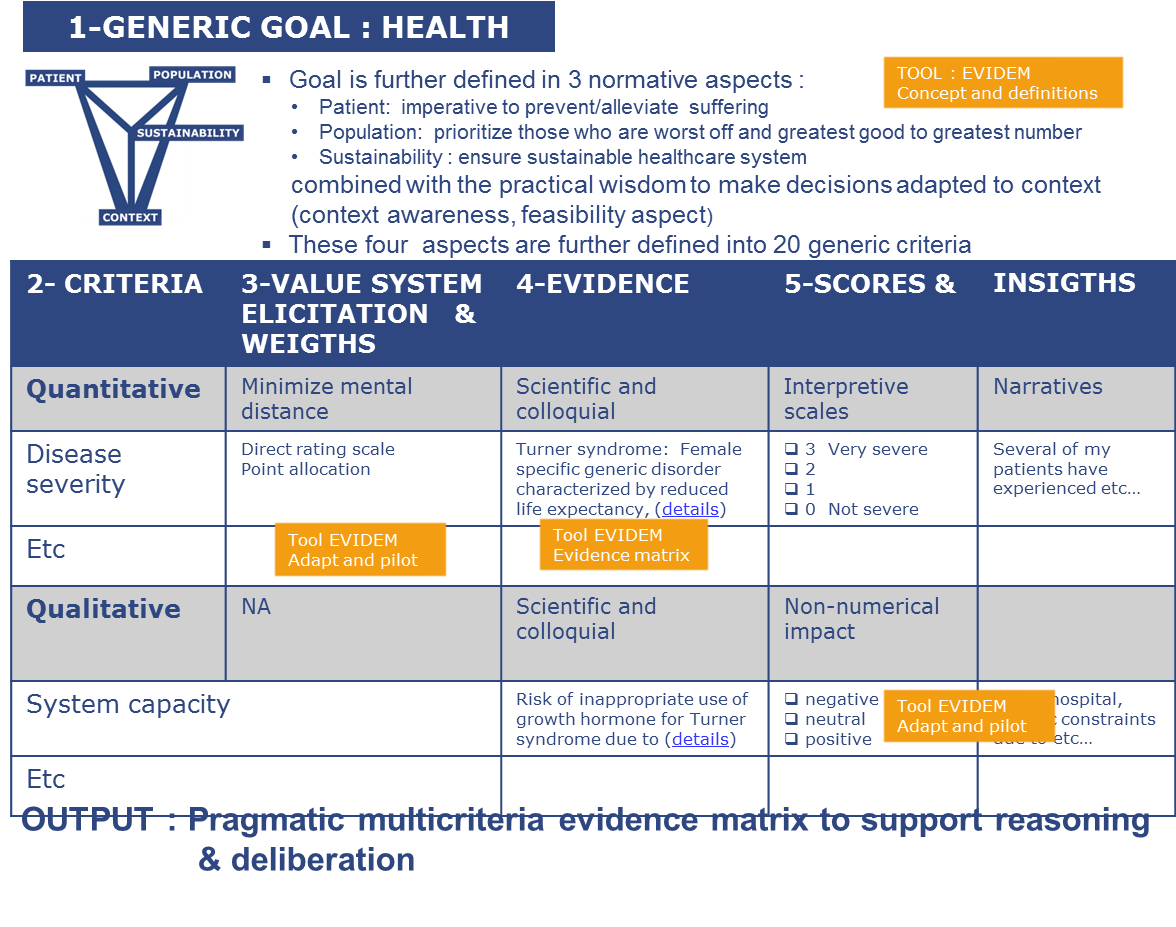

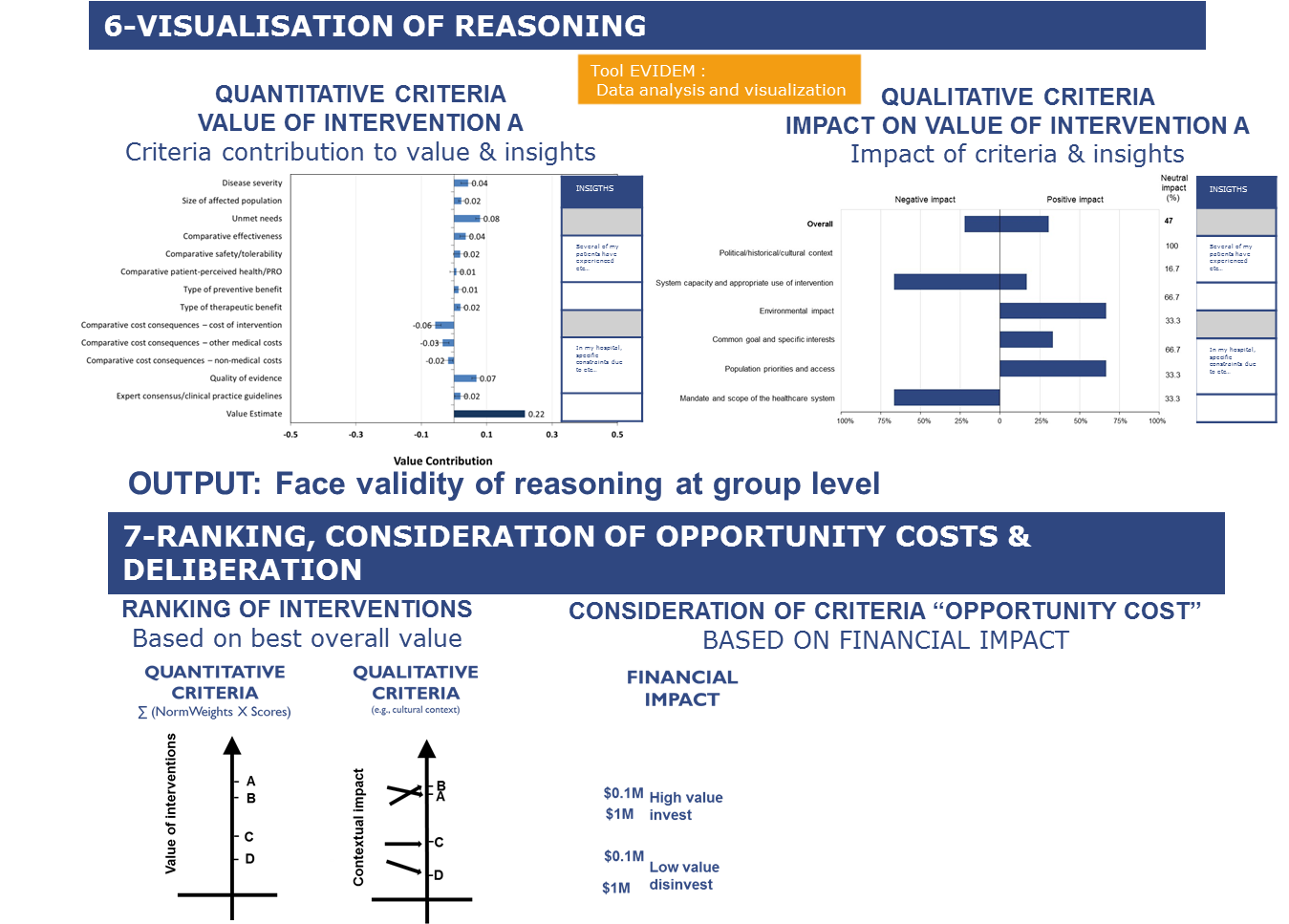


# CRITERIA Conceptualization

The relevance of the decision, or substantive legitimacy, is assumed by defining the common goal of healthcare systems, the ethical imperatives underlying such goal, and transforming them into generic criteria.

The goal is defined in 3 normative aspects (1- imperative to alleviate/prevent suffering of patients; 2 – prioritize those who are worst off while ensuring greatest good to greatest number; Ensure sustainability) combined with the wisdom of making decision adapted to knowledge and context (feasibility aspect). These aspects are defined into 20 criteria in agreement with MCDA methodological principles of non-redundancy, independence, operationalizability and completeness.

**Universal criteria, defined as those** for which low and high ends of the scales can be defined *a priori* (*i.e.*, they are universally agreed upon), constitute the EVIDEM Core Model (normative universal criteria).

## Normative universal criteria –core model

The framework assumes that highest rank (or priority) should be given to healthcare interventions:

- For **severe disease**
- For **common disease**
- For disease with many **unmet needs**
- Either conferring major **risk reduction** or major **alleviation of suffering**; *this design allows to consider both* *preventive and therapeutic interventions, without giving a priori priority to either one*
- Conferring major improvement in **efficacy/effectiveness** over standard of care
- Conferring major improvement in **safety & tolerability** over standard of care
- Conferring major improvement of **patient-reported outcomes/patient-perceived health** over standard of care
- That result in **savings** in **treatment** expenditures as well as other **medical** and **non-medical** expenditures
- For which there is sufficient data, that is **fully reported** and **valid and relevant**
- Recommended in consensus guidelines by **experts**

These criteria constitute the MCDA Core Model. Most of these criteria include sub-criteria that can be added by end-users to the MCDA Core Model (see section below for details on definition, sub-criteria, ethical foundation and rationale for each criterion).

*Note: Cost-effectiveness is a composite measure of date considered in other criteria and does not comply with the non-redundancy design requirement of MCDA. It may be included in the framework since many decisionmaking processes currently rely on this composite measure, but it should be ultimately replaced by the criteria with which it overlaps.*

## Contextual criteria – CONTEXTUAL TOOL

The Contextual Tool is used as a guide to tailor the framework to the context of decisionmaking. It includes seven generic criteria/themes, with a number of sub-criteria from which end-users can select those most relevant to their setting. Contextual criteria and sub-criteria, once identified, can remain in the Contextual Tool for qualitative consideration, or be added to the MCDA Core Model for quantitative assessment if they can be operationalized (*i.e.*, the low and high ends of the scale are clearly defined and stable across interventions). The Contextual Tool includes normative and feasibility contextual criteria*.*

### Normative contextual criteria

- **Mandate and scope of healthcare system**: *the mandate and scope of the healthcare system/plan need to be defined explicitly; once defined, this criterion can be added to the MCDA Core Model for quantitative assessment to assign more value to interventions that are fully aligned with the scope and mission of the healthcare system.*
- Population **priorities and access** of healthcare system/plan: *the* *priorities of decisionmakers/society need to be identified explicitly (*e.g.*, priority to children, population in remote communities); once defined, this criterion can be added to the MCDA Core Model for quantitative assessment to assign more value to interventions that are fully aligned with the identified priorities.*
- **Common goal and specific interests**: *usually not quantifiable* *but important to realize and to address to ensure that the decision is fair-minded and driven by the common goal and not unduly influenced by specific interests.*
- **Environmental impact**: *Extent to which the production, use, or implementation of the intervention causes environmental damages; if clearly operationalized in a given context, this criterion can be added to the MCDA Core Model for quantitative assessment to assign more value to interventions that do not cause environmental damage.*

### Feasibility contextual criteria

Feasibility aspects need to be tailored to the specific context, including:

- **System** **capacity** and **appropriate use of intervention**: *a number of aspects may need to be considered under this umbrella (*e.g.*, skill requirements, organizational requirements - see section below for other specific criteria); criteria identified as critical by end users can either be kept in the Contextual Tool for qualitative considerations or, if operationalizable, can be added to the MCDA Core Model for quantitative assessment.*
- **Political, historical and cultural** **context**: *a number of aspects need to be considered under this umbrella (*e.g.*, cultural acceptability, precedence, current political priorities, impact on healthcare research – see section below for other specific criteria); criteria identified as critical by end-users can either remain in the Contextual Tool for qualitative consideration or, if operationalizable, be added to the MCDA Core Model for quantitative assessment.*

***Final system consideration***

- **Opportunity** **costs** and **affordability**: *the* *actual financial impact of an intervention and the available budget need to be aligned using budgeting tools; considerations of opportunity costs and silo budgeting should be part of this financial exercise* *(note: the normative aspects of economic considerations [*i.e.*, encouraging savings] are included in the MCDA Core Model).*

# Criteria details: definition, scoring instructions and rationale

## Criteria of the MCDA CORE MODEL

**Criteria were grouped (clustered) by objective to facilitate their consideration and to comply with MCDA requirements on clustering.**

| **Objectives / Criteria** | **Definition** | **Scoring scale & instructions**  **Low High** | | **Ethical foundation, rationale for design & inclusion in the MCDA Core Model** |
| --- | --- | --- | --- | --- |
| **CORE MODEL - Normative universal OBjECTIVES ­– quaNtitative appraisal** | | | | |
| **Need for intervention** | | | | |
| **Disease severity**  *Possible sub-criteria:*   - *Effect of disease on life-expectancy* - *Effect of disease on morbidity (includes disability and function)* - *Effect of disease on patients’ quality of life* - *Effect of disease on caregivers’ quality of life* | Severity of the health condition of patients treated with the intervention (or severity of the health condition that is to be prevented) with respect to mortality, morbidity, disability, function, impact on quality of life, clinical course (*i.e.*, acuteness, clinical stages). | **Not severe**  **Very severe**  Score the severity of disease by considering its impact on the patient; consider the disease as a whole, not only the aspect of the disease that the intervention is targeting.  (Economic aspects are not considered here – see *Economic consequences of intervention* cluster).  *Score from an absolute point of view (not relative to comparative interventions)*  *Scoring scale example (for information only):*  *5 Very severe*  *4*  *3*  *2*  *1*  *0 Not severe*  | | Ethical foundation: Alleviate suffering in those who are **worst-off** (aspect of *distributive justice* – *fairness*).  Rationale for inclusion: An intervention for patients with a severe, life-threatening or disabling disease or an intervention to prevent such a disease would have greater value than an intervention for patients with a less severe disease or to prevent a less severe disease.  Disease severity contributes to the value of an intervention since it is usually agreed that alleviating a severe condition is more important than alleviating a mild condition. The scale direction is thus based on the ethical principle of fairness which gives more value to interventions that are alleviating those who are worst off.  Inclusion of this criterion with such scale direction implies that, for example, growth hormone treatment for patients with Turner syndrome would have a greater value estimate than the same treatment for people with idiopathic short stature because people with Turner syndrome, due to increased morbidity and mortality are on the whole, are worse off than those who are only short.  The overall economic burden of the disease is a composite measure of disease severity, size of affected population and cost of current treatment and is therefore not included as an independent criterion. The value of the intervention with respect to disease severity and size of population is captured in separate criteria. The value in reducing the economic burden is captured in the *Economic consequences of intervention* cluster.  *This criterion captures a measure of absolute value (not relative to comparative interventions).* |
| **Size of affected population**  *Possible sub-criteria:*   - *Prevalence* - *Incidence* | Number of people affected by the condition (treated or prevented by the intervention) among a specified population at a specified time; can be expressed as annual number of new cases (annual incidence) and/or proportion of the population affected at a certain point in time (prevalence). | **Common disease**  **Very rare disease**  Score the size of the population affected by the condition keeping in mind that this would be the size of the population potentially benefitting from the intervention. (Economic aspects are not considered here – see *Economic consequences of intervention* cluster).  *Score from an absolute point of view (not relative to comparative interventions)*  *Scoring example (for information only):*  *5 X > 500/10,000*  *4 X < 500/10,000*  *3 X < 100/10,000*  *2 X < 10/10,000*  *1 X < 5/10,000 (rare)*  *0 X < 2/100,000 (ultra rare)*  | | Ethical foundation: Alleviate suffering **in as many individuals** as possible (aspect of *utilitarianism*).  Rationale for inclusion: An intervention potentially benefiting a larger number of people would have greater value than an intervention potentially benefiting fewer people.  Size of population contributes to the value of an intervention because the larger the population, the larger the contribution to overall health improvement. The scale direction is thus based on the ethical principle of utility which aims at “doing the greatest good for the greatest number of people”.  The economic impact of the size of the population is captured in the *Economic consequences of intervention* cluster.  *This criterion captures a measure of absolute value (not relative to comparative interventions).* |
| **Unmet needs** *Possible sub-criteria:*   - *Unmet needs in efficacy* - *Unmet needs in safety* - *Unmet needs in patient reported outcomes* - *Patient demand* | Shortcomings of comparative interventions in their ability to prevent, cure, or ameliorate the condition targeted; also includes shortcomings with respect to safety, patient-reported outcomes and convenience. | **No unmet needs**  **Many unmet needs**  Score unmet needs of comparative interventions with respect to treatment or prevention of the targeted condition. (Limitations due to cost of intervention are not considered here – see *Economic consequences of intervention* cluster).  *Scoring example (for information only):*  *5 Many unmet needs*  *4*  *3*  *2*  *1*  *0 No unmet needs*  **A: Alzheimer's disease**  **B: Appendicitis** 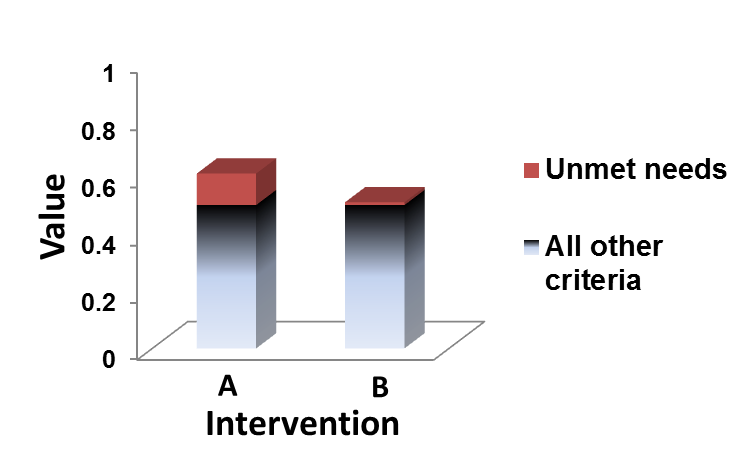 | | Ethical foundation: Alleviate suffering in individuals **with limited alternative interventions** (aspect of *distributive justice*).  Rationale for inclusion: An intervention that targets a health condition for which there are no alternative interventions or where alternative interventions have major limitations would have greater value than an intervention for which there are alternatives that have no or minor limitations.  Limitations of other existing interventions contribute to the value of an intervention because it is generally agreed that more value can be drawn in the context of current limitations. This criterion thus captures the need of the target population in the context of available interventions to manage the disease.  This is not captured by the disease severity criterion because a disease (*e.g.*, diabetes) could be severe, but could nevertheless be effectively treated with current interventions, while a less severe condition (*e.g.*, seasonal allergy) could have limited treatment options. To what extent the intervention may be able to overcome limitations of comparators and meet currently unmet needs is not assessed here but by the *Comparative outcomes of intervention* criteria.  To avoid redundancy, this criterion does not address limitations due to high cost because the value of replacing costly interventions by lower-cost alternatives is captured by *Comparative cost consequences – cost of intervention*.  *This criterion captures a measure of absolute value (not relative to comparative interventions),* i.e.*, the position of the intervention in the realm of existing interventions.* |
| **Comparative outcomes of intervention** | | | | |
| **Comparative effectiveness** *Possible sub-criteria:*   - *Magnitude of health gain* - *Percentage of the target population expected to achieve the anticipated health gain* - *Onset and duration of health gain* - *Sub-criteria for the measure of efficacy specific to the therapeutic area* | Capacity of the intervention to prevent or to produce a desired (beneficial) change in signs, symptoms or course of the targeted condition above and beyond beneficial changes produced by alternative interventions. | **Much better than comparator**  **Much worse than comparator**    Score the efficacy/effectiveness of the intervention in relation to comparative interventions presented (consider clinical significance of outcomes measures).  *Score from a relative point of view (relative to comparative interventions)*  *Scoring example (for information only):*  *5 Much better than comparator (positive contribution)*  *4*  *3*  *2*  *1*  *0 No difference*  *-1*  *-2*  *-3*  *-4*  *-5 Much worse than comparator (negative contribution)*  **A,B: HIV, leukemia**  **treatments** 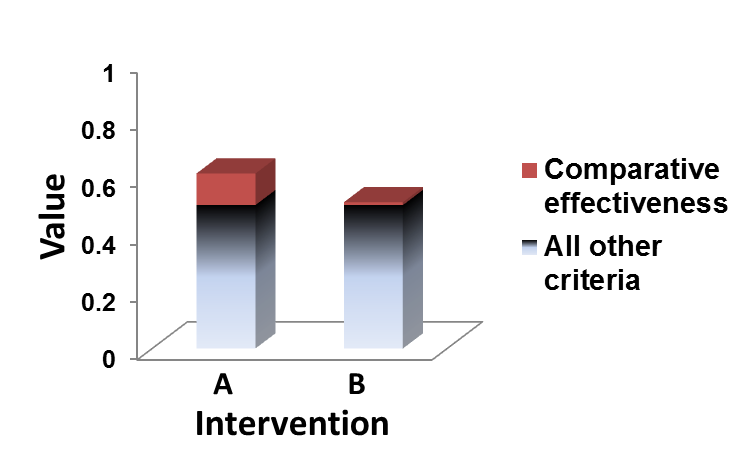 | Ethical foundation: Alleviate suffering to the **greatest extent** (aspect of *deontology*, *beneficence*).  Rationale for inclusion: An intervention that provides major improvement in efficacy/effectiveness would have greater value than an intervention with lower efficacy/effectiveness than comparators.  Improvement of efficacy/effectiveness contributes to the value of an intervention because it captures the extent to which an intervention is able to achieve its clinical goal relative to current comparators. Which types of outcomes were measured in trials is critical to assess value (*e.g.*, the use of a surrogate outcome measure may limit value as clinical benefit is not measured directly).  This criterion, together with *Comparative safety/tolerability* and *Comparative patient-perceived health/patient-reported outcomes*, were defined as “comparative” (*i.e.*, versus comparators), because decisions are usually taken in a context of existing treatments and practices. This design assumes that, everything else being equal, the value contribution of efficacy to the overall value of the intervention derives from what it adds rather than its absolute efficacy/ effectiveness.  The potential value of an intervention derived from fewer adverse events or improved patient-reported outcomes is captured separately and independently since their contribution to value may diverge (*e.g.*, a treatment may be highly efficacious but have many side effects or vice versa).  *This criterion captures a measure of the relative value of an intervention (*i.e.*, compared to interventions it is meant to replace) and includes a negative scale that reflects the full possible range of comparative effects.* | |
| **Comparative safety / tolerability** *Possible sub-criteria:*   - *Adverse events* - *Serious adverse events* - *Fatal adverse events* - *Short-term safety* - *Long-term safety* - *Tolerability* | Capacity of the intervention to produce a reduction in intervention-related harmful or undesired health effects compared to alternative interventions. | **Much better than comparator**  **Much worse than comparator**  Score the safety and tolerability of the intervention in relation to comparative interventions presented (consider clinical significance of adverse events).  *Score from a relative point of view (relative to comparative interventions)*  *Scoring example (for information only):*  *5 Much better than comparator (positive contribution)*  *4*  *3*  *2*  *1*  *0 No difference*  *-1*  *-2*  *-3*  *-4*  *-5 Much worse than comparator (negative contribution)*  **A,B: New generation**  **antipsychotics** 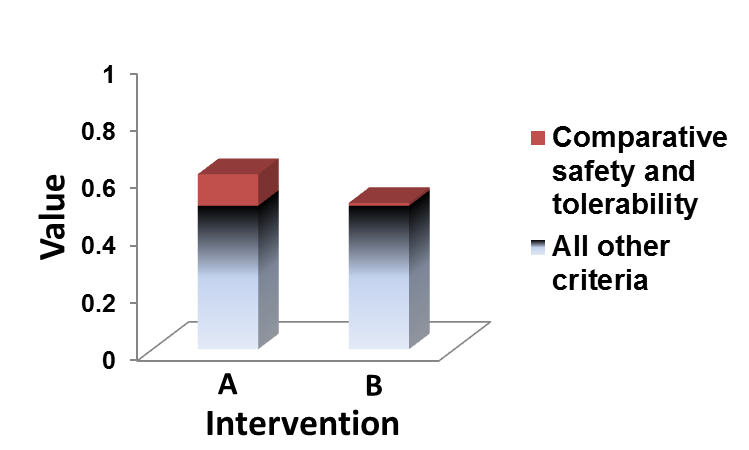 | Ethical foundation: Hippocratic Oath “***Do no harm***” (aspect of *deontology*, *non-maleficence*).  Rationale for inclusion: An intervention that provides major improvement in safety/tolerability would have greater value than an intervention with lower safety/tolerability than comparators.  Comparative safety contributes to the value of an intervention because it captures the extent to which an intervention is able to reduce harmful or undesired health effects (independently from *Comparative effectiveness* and *Comparative patient-perceived health/patient-reported outcomes*).  *This criterion captures a measure of the relative value of an intervention (*i.e.*, compared to interventions it is meant to replace) and includes a negative scale to reflect the full possible range of comparative effects.* | |
| **Comparative patient-perceived health / patient-reported outcomes** *Possible sub-criteria:*   - *Improvement in health-related quality of life* - *Impact on autonomy* - *Impact on dignity* - *Convenience / ease of use / mode & setting of administration* | Capacity of the intervention to produce beneficial changes in patient-perceived health and patient-reported outcomes (PROs) (*e.g.*, quality of life) above and beyond beneficial changes produced by alternative interventions; also includes improvement in convenience to patients. | **Much better than comparator**  **Much worse than comparator**  Score patient-reported outcomes of the intervention in relation to comparative interventions presented. (Consider quality of life data & convenience).  *Score from a relative point of view (relative to comparative interventions)*  *Scoring example (for information only):*  *5 Much better than comparator (positive contribution)*  *4*  *3*  *2*  *1*  *0 No difference*  *-1*  *-2*  *-3*  *-4*  *-5 Much worse than comparator (negative contribution)*  | Ethical foundation: Alleviate suffering as **perceived by the patient** (Hippocratic Oath *“*for the good of my patient*”)* (aspect of *deontology*, *respect for autonomy* & *dignity*).  Rationale for inclusion: An intervention that provides major improvement in PROs would have greater value than an intervention with worse PROs than comparators.  Comparative PROs contributes to the value of an intervention because it measures the extent to which an intervention is able to enhance patient perception of his/her health. This criterion captures the patient perspective regarding the impact of the intervention on his/her life, while *Comparative effectiveness* and *Comparative safety/ tolerability* represent results of the clinical assessment. Although there is often a correlation between these criteria and PRO improvement, there is no necessary link between these concepts; thus, for example, a treatment could be highly efficacious but result in poor PRO or vice versa.  *This criterion captures a measure of the relative value of an intervention (*i.e.*, compared to interventions it is meant to replace) and includes a negative scale to reflect the full possible range of comparative effects.* | |
| **Type of benefit of intervention** | | | | |
| **Type of preventive benefit** | Nature of the preventive benefit or risk reduction provided by the intervention (*e.g.*, eradication, prevention, reduction in disease transmission, reduction in the prevalence of risk factors). Public health perspective.  **No preventive benefit** | **Eradication**  Score what the intervention brings to the population with regard to risk reduction (consider the nature of the risk that is reduced, from a public health perspective).  *Score from an absolute point of view (not relative to comparative interventions)*  *Scoring example (for information only):*  *5 Eradication*  *4*  *3*  *2*  *1*  *0 No preventive benefit*  **A: Vaccine**  **B: Hypertension drug** 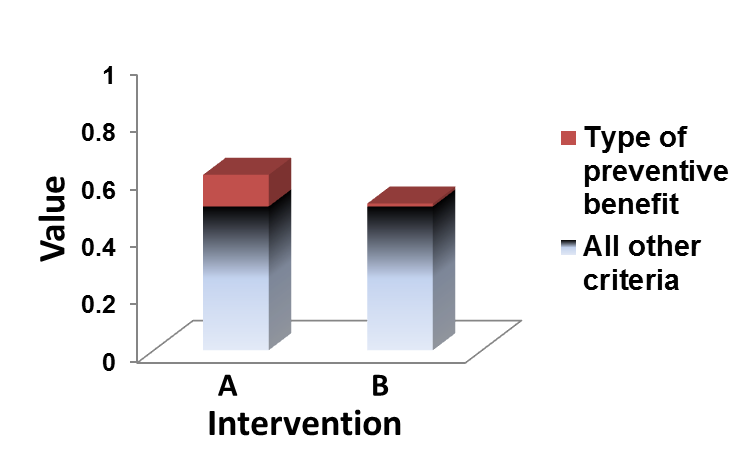 | Ethical foundation: To **protect** health and **prevent suffering** (aspect of *deontology*).  Rationale for inclusion: An intervention that provides a major reduction in disease risk to the population would have greater value than an intervention that provides no reduction in disease risk to the population.  Risk reduction contributes to the value of an intervention as it captures the benefit of preventing or reducing the risk of a disease at the population level. From a public health perspective, it is agreed that an intervention that eliminates the risk of a disease has more value than an intervention that provides no risk reduction.  This criterion is distinct from *Comparative effectiveness*, because it focuses on the nature (type) of the benefit rather than the extent to which this benefit is realized.  *This criterion captures a measure of absolute value (not relative to comparative interventions).* | |
| **Type of therapeutic benefit** | Nature of the clinical benefit provided by the intervention at the patient level (*e.g.*, symptom relief, prolonging life, cure).  **No therapeutic benefit** | **Cure**  Score what the intervention brings to the patient with regard to her/his current medical condition (consider a large range of benefits, *e.g.*, from cure to symptom relief).  *Score from an absolute point of view (not relative to comparative interventions)*  *Scoring example (for information only):*  *5 Cure*  *4*  *3*  *2*  *1*  *0 No therapeutic benefit*  **A: Cure (Holy Grail)**  **B: Minor symptom relief** 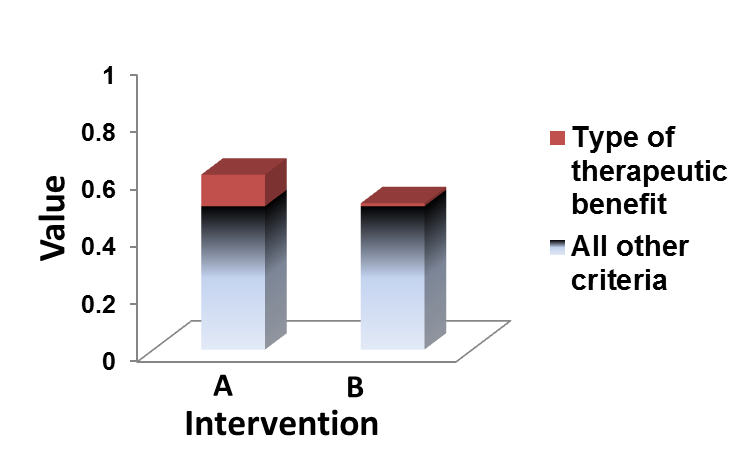 | | Ethical foundation: Aim to **eliminate** rather than merely alleviate suffering (aspect of *deontology*).  Rationale for inclusion: An intervention that provides a major medical service to the patient (*e.g.*, cure) would have greater value than an intervention that provides a minor service (*e.g.*, relief from a disease symptom).  The type of medical service (or clinical benefit) at the patient level contributes to the value of an intervention as it is generally agreed that an intervention that cures a disease has more value than an intervention that provides symptom relief only.  This criterion is distinct from *Type of preventive benefit* as it captures the type of service at the patient level, rather than at the population level (individual effect as opposed to population effect considered in *Type of preventive benefit*).  This criterion is distinct from *Comparative effectiveness*, because it focuses on the nature of the benefit itself rather than the extent to which this benefit is realized. For example, an intervention to treat migraine attacks may be a major improvement (*i.e.*, improved efficacy) in terms of how quickly the pain subsides or the percentage of patients responding; nevertheless, if this intervention does not cure the disease it is deemed to provide only an improvement of the condition.  *This criterion captures a measure of absolute value (not relative to comparative interventions).* |
| **Economic consequences of intervention** | | | | |
| **Comparative cost consequences – cost of intervention** *Possible sub-criteria:*   - *Net cost of intervention* - *Acquisition cost* - *Implementation/ maintenance cost* | Net cost of covering the intervention (excluding other spending). This represents the differential between expected expenditure for the intervention and potential cost savings that may result from replacement of other intervention(s) currently covered by the health plan. Limited to cost of intervention (*e.g.*, acquisition cost, implementation and maintenance cost).  *Note: in countries where part of the intervention cost is paid by patients (e.g. copayment), this criteria should be adjusted consequently*. | **Substantial additional expenditures**  **Substantial savings**  Score the net impact of the cost of the intervention (consider only the cost of intervention such as acquisition/implementation cost – other spending considered in *Comparative cost consequences – other medical costs* and *Comparative cost consequences – non-medical costs*).  *Score from a relative point of view (relative to comparative interventions)*  *Scoring example (for information only):*  *5 Substantial savings (positive contribution)*  *4*  *3*  *2*  *1*  *0 No change in spending*  *-1*  *-2*  *-3*  *-4*  *-5 Substantial additional expenditures (negative contribution)*  Note: Provide country or health plan range of spending for healthcare interventions to give a frame of reference  | Ethical foundation: **Use** scarce resources **wisely** to maximize health from a specific budget perspective (practical wisdom, aspect of *utilitarianism*).  Rationale for inclusion: Interventions that reduce treatment costs have greater value than those that increase treatment costs.  This criterion captures the value related to the extent of additional or reduced expenditures stemming from covering an intervention (usually includes consideration of reductions from replacement of comparative interventions currently covered by the health plan).  This economic impact is captured separately from the impact on other medical and non-medical spending because these concepts are often considered separately. Also, there is more certainty on the impact of purchasing than on potential impact on other spending.  *This criterion captures a measure of the relative value of an intervention (*i.e.*, compared to interventions it is meant to replace) and includes a negative scale to reflect the full possible range of comparative effects.* | |
| **Comparative cost consequences – other medical costs** *Possible sub-criteria:*   - *Impact on primary care expenditures* - *Impact on hospital care expenditures* - *Impact on long-term care expenditures* | Impact of the intervention on other medical costs (excluding intervention cost, such as hospitalization, specialist consultations, adverse events costs, long-term care, etc.  *Note: in countries where part of the medical costs are paid by patients (e.g. copayment), this criteria should be adjusted consequently*. | **Substantial savings**  **Substantial additional expenditures**    Score the impact of the intervention on other medical spending (consider a broad perspective if data is available). Exclude intervention cost because it is considered in *Comparative cost consequences – cost of intervention*.  *Score from a relative point of view (relative to comparative interventions)*  *Scoring example (for information only):*  *5 Substantial savings (positive contribution)*  *4*  *3*  *2*  *1*  *0 No change in spending*  *-1*  *-2*  *-3*  *-4*  *-5 Substantial additional expenditures (negative contribution)*  **A: Major reduction of**  **hospitalization cost**  **B: Minor reduction of**  **diagnostic test cost** 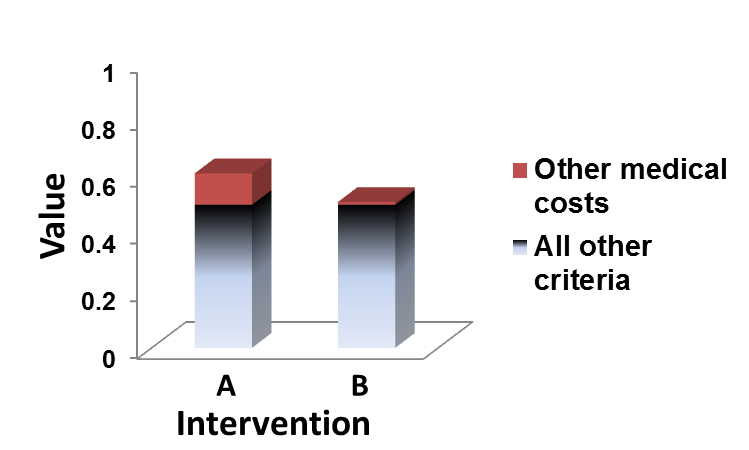 | Ethical foundation: **Use** scarce medical resources **wisely** from a broad perspective (aspect of *utilitarianism*).  Rationale for inclusion: Interventions that free up other medical resources have greater value than those that require the use of additional medical resources.  This criterion captures the economic consequences of the intervention in terms of other medical costs for the health plan as well as for other payers (*e.g.*, patients), excluding the cost of the intervention itself and the interventions that it is directly replacing. (This is captured in *Comparative cost consequences – cost of intervention*.) It captures the capacity of the intervention to reduce the burden of the disease in terms of medical costs (*e.g.*, hospitalization).  *This criterion captures a measure of the relative value of an intervention (*i.e.*, compared to interventions it is meant to replace) and includes a negative scale to reflect the full possible range of comparative effects.* | |
| **Comparative cost consequences – non-medical costs** *Possible sub-criteria:*   - *Impact on productivity* - *Financial impact on patients* - *Financial impact on caregivers* - *Costs to the wider social care system* | Impact of the intervention on non-medical costs (excluding intervention cost and other medical costs) such as disability costs, social services, lost productivity, caregiver time, etc. | **Substantial savings**  **Substantial additional expenditures**  Score the impact of the intervention on other non-medical spending (consider a broad perspective if data is available). Exclude intervention cost because it is considered in *Comparative cost consequences – cost of intervention*.  *Score from a relative point of view (relative to comparative interventions)*  *Scoring example (for information only):*  *5 Substantial savings (positive contribution)*  *4*  *3*  *2*  *1*  *0 No change in spending*  *-1*  *-2*  *-3*  *-4*  *-5 Substantial additional expenditures (negative contribution)*  **A: Major reduction of**  **lost productivity costs**  **B: Minor reduction of**  **caregiver time costs** 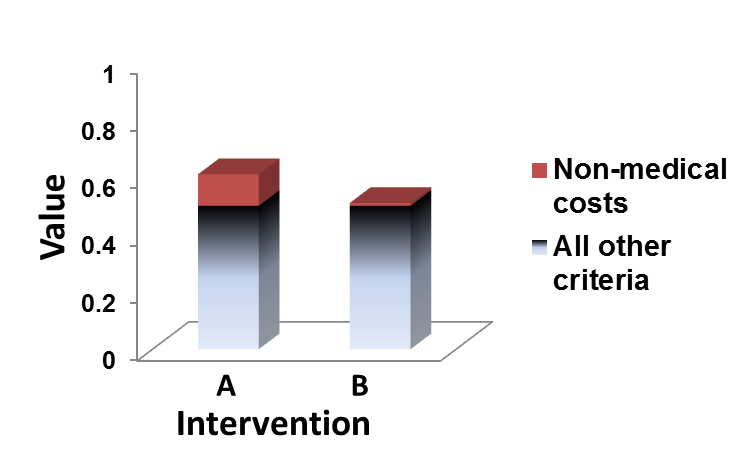 | Ethical foundation: **Preserve** societal and individual resources **wisely** from a broad perspective (aspect of *utilitarianism*).  Rationale for inclusion: An intervention that preserves and frees up non-medical resources has a greater value than those that require the use of additional non-medical resources.  This criterion captures the economic consequences of the intervention in terms of non-medical costs for the patient as well as society as a whole, excluding the cost of the intervention itself and the interventions that it is directly replacing. (This is captured in *Comparative cost consequences – cost of intervention*.) It captures the capacity of the intervention to reduce the burden of the disease in terms of non-medical costs (*e.g.*, lost productivity due to disability).  *This criterion captures a measure of the relative value of an intervention (*i.e.*, compared to interventions it is meant to replace) and includes a negative scale to reflect the full possible range of comparative effects.* | |

| **Knowledge about intervention** | | | |
| --- | --- | --- | --- |
| **Quality of evidence** *Possible sub-criteria:*   - *Validity* - *Relevance* - *Completeness of reporting* - *Type of evidence* | Extent to which evidence on the intervention is relevant to the decisionmaking body (in terms of population, disease stage, comparator interventions, outcomes, etc.) and valid with respect to scientific standards (*i.e.*, study design, etc.) and conclusions (*i.e.*, agreement of results between studies). This includes consideration of uncertainty (*e.g.*, conflicting results across studies, limited number of studies and patients). Complete reporting of evidence is a pre-requisite to assess coherence and validity. | **Highly relevant and valid**  **Not relevant and/or invalid**  Score the relevance and validity of evidence based on critical analysis and quality ratings generated using the quality assessment tools. Relative importance of quality ratings for types of evidence assessed is left to your judgment (*e.g.*, if quality ratings for clinical data is high and low for economic data, you may want to give more weight to clinical data).  *Score from an absolute point of view (not relative to comparative interventions)*  *Scoring example (for information only):*  *5 Highly relevant and valid*  *4*  *3*  *2*  *1*  *0 Not relevant and/or invalid*  **A: RCTs & observational**  **studies; real-life cost**  **data; strong epi data**  **B: 1 RCT; economic model**  **with many assumptions;**  **limited epi data** 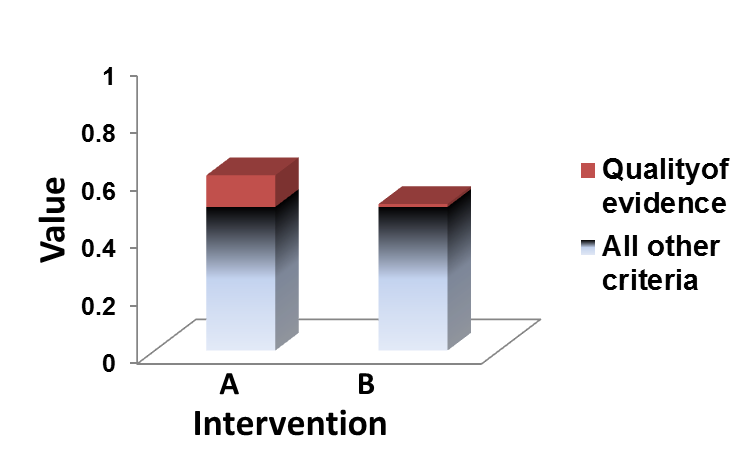 | Ethical foundation: Consider the **strength of claims** about the intervention **based on** **formal evidence** (*practical wisdom*, *imperative of evidence-based decisionmaking*).  Rationale for inclusion: An intervention for which evidence reporting is complete and consistent, relevant to the decision to be made and valid with respect to international scientific standards would have greater value than an intervention for which evidence reporting is incomplete and inconsistent and the evidence has low relevance and validity.  Relevance and validity of evidence contribute to the value of an intervention since limited validity and relevance reduces confidence in an intervention’s ability to improve health in a given context. Quality of reporting contributes to the value of an intervention as far as poor reporting will limit understanding of data, and thereby decrease the value of intervention.  This is kept separate from consideration of the actual extent of health gain or the economic impact of an intervention because scientific judgment on data quality is quite different from gauging the extent of a benefit. For example, if there was only one study indicating a health gain of 30%, or several studies averaging a health gain of 30%, the scoring for *Comparative effectiveness* would likely be the same, but not the scoring on validity and strength of evidence, which captures separately the overall level of uncertainty of the intervention outcomes.  *This criterion captures a measure of absolute value (not relative to comparative interventions).* |
| **Expert consensus / clinical practice guidelines** | Concurrence of the intervention (or similar alternatives) with the current consensus of experts on what constitutes state-of-the-art practices in the management of the targeted health condition; guidelines are usually developed via an explicit process and are intended to improve clinical practice. | **Strong recommendation for intervention above all other alternatives**  **Not recommended**  Score the strength of recommendation for the intervention. Consider whether interventions of the same type as the proposed intervention are recommended (in general) as well as whether the proposed intervention is recommended above other alternatives.  *Scoring example (for information only):*  *5 Strong recommendation for intervention above all other alternatives*  *4*  *3*  *2*  *1*  *0 Not recommended*  | Ethical foundation: Consider **strength of claims** about the intervention **based on expert knowledge and consensus** (aspect of *practical wisdom*).  Rationale for inclusion: Interventions strongly recommended on the basis of current expert consensus have greater value than those not recommended by clinical experts.  This criterion captures the strength of recommendations based on synthesis and interpretation of current scientific knowledge as well as bedside expertise not available from the published clinical study evidence (*Quality of evidence*). A strong positive clinical guideline recommendation raises confidence in the value of an intervention.  *This criterion captures a measure of absolute value (not relative to comparative interventions),* i.e.*, the position of experts on the intervention in the realm of existing interventions.* |
|  |  |  |  |

## Criteria of the CONTEXTUAL TOOL

| **Objectives / Criteria** | **Definition** | **Instructions for consideration** | **Ethical foundation, rationale for design & inclusion in Contextual tool** |
| --- | --- | --- | --- |
| **CONTEXTUAL TOOL - contextual objectives – qualitative appraisal** | | | |
| **Normative contextual criteria** | | | |
| **Mandate and scope of healthcare system** | Alignment of the intervention with the mandate/scope of the healthcare system. The goal of healthcare is to maintain normal functioning. Mission and scope of healthcare plans/systems derive from this principle. | **In the CONTEXTUAL TOOL**  Would this consideration have a positive, neutral or negative impact on the appraisal of the intervention?  **Positive**: aligned with mandate and scope  **Negative**: in potential disagreement with mandate and scope  Consider whether the intervention is actually aligned with the scope of the healthcare plan/system.  Requires that the mission and scope of the system be defined.  *Example*:  *Enhancement of otherwise normal traits (*e.g.*, growth hormone for short stature) may not be considered a medical issue but rather a social issue, and thus not aligned with the mandate of a healthcare system/plan. One would question here whether such consideration impacts the value/appraisal of growth hormone for individuals with short stature.*  ***OR***  **In the quantitative MCDA MODEL**  Once the mandate and scope are defined, they can be added to the MCDA Core Model for quantitative assessment to assign more value to interventions that are fully aligned with the mandate and scope of the healthcare system. | Ethical foundation: **Promote and protect health** of the population served (aspect of *utilitarianism*, *beneficence*).  Rationale for inclusion: An intervention falling within the mandate and scope of the healthcare system has greater value than one not aligned with these.  Ensures consideration of whether the intervention is actually producing a good that is a health benefit falling under the mandate and within the scope of the health plan/system (it often implicitly does).  The tool is used here to prompt identification of the mandate and scope of the healthcare system/plan. Once identified explicitly, this can become a criterion that may be operationalized (by defining low and high ends of scale) and added to the MCDA Core Model for quantitative assessment. |
| **Population priorities and access** *Possible sub-criteria:*   - *Current priorities of health system (*e.g. *low socioeconomic status; specific age groups)* - *Special populations (*e.g.*, ethnicity)* - *Remote communities* - *Rare diseases* - *Specific therapeutic areas* | Alignment of the intervention with current priorities of health system/plan. Priorities for specific groups of patients are defined by societies/decisionmakers and reflect their moral values. Such considerations are aligned with the principle of justice, which considers treating like cases alike and different cases differently and often gives priority to those who are worst-off. | **In the CONTEXTUAL TOOL**  Would this consideration have a positive, neutral or negative impact on the appraisal of the intervention?  **Positive**: aligned with established priorities  **Negative**: in potential disagreement with established priorities  Consider how the intervention fits with priorities in regard to population and access to care.  *Example*:  *Prioritize intervention targeted at vulnerable populations that can be delivered in remote regions with limited facilities.*  ***OR***  **In the quantitative MCDA MODEL**  Once priorities are defined, they can be added to the MCDA Core Model for quantitative assessment to assign more value to interventions aligned with identified priorities. | Ethical foundation: Prioritize those who are considered worst off in a given society/community (*distributive justice*).  Rationale for inclusion: Interventions targeting established priority populations or disease areas have greater value than those not aligned with these established priorities.  Ensures consideration of priorities defined by decisionmakers or society.  The tool is used here to prompt identification of the priorities (*e.g.*, priority to children, population in remote communities) of the healthcare system/plan. Sub-criteria are proposed to facilitate the process of identification of priorities. Once identified explicitly, these priorities can become criteria that may be operationalized (by defining low and high end of scale) and added to the MCDA Core Model for quantitative assessment. |
| **Common goal and specific interests** *Possible sub-criteria:*   - *Stakeholder pressures* - *Stakeholders barriers* - *Conflict of interest* | Pressures or barriers from groups of stakeholders or individuals are often part of the context surrounding healthcare interventions. Being aware of pressures and interests at stake and how they may affect decisionmaking helps ensure that decisions are aligned with the common goal. | Would this consideration have a positive, neutral or negative impact on the appraisal of the intervention?  **Positive**: aligned with the common goal  **Negative**: in potential disagreement with the common goal  Consider whether such pressures affect the decision.  *Examples:*  *Pressures from specialists to use innovative technologies; pressures from patient groups for compassionate use.* | Ethical foundation: Awareness of stakeholder pressures / barriers helps **ensure that decisions are fair-minded** and driven by the common goal and not unduly influenced by specific interests (aspect of *practical wisdom*).  Rationale for inclusion: Interventions aligned with the common goal have greater value than those aligned with special interests.  Ensures explicit consideration of such pressures/barriers and awareness of the interests at stake.  These pressures/barriers are usually not quantifiable but important to recognize and address to ensure that the decision is not unduly influenced by special interests. |
| **Environmental impact** *Possible sub-criteria:*   - *Environmental impact of production* - *Environmental impact of use* - *Environmental impact of implementation* | The extent to which the production, use or implementation of the intervention causes environmental damages. | **In the CONTEXTUAL TOOL**  Would this consideration have a positive, neutral or negative impact on the appraisal of the intervention?  **Positive**: aligned with the protection of the environment **Negative**: in potential disagreement with the protection of the environment  Consider whether potential environmental impact related to the intervention affects the decision  *Example:*  *The intervention necessitates the use of radioactive isotopes which have a negative impact on the environment and require special containment.*  ***OR***  **In the quantitative MCDA MODEL**  If deemed of sufficient importance, and if operationalizable, this criterion may be added to the MCDA Core Model for quantitative assessment, which will assign more value to interventions causing no environmental damage. | Ethical foundation: **Protect the environment** (aspect of *utilitarianism*, *beneficence*)  Rationale for inclusion: Interventions which are produced, used or implemented without causing environmental damage have greater value than those causing environmental damage.  This criterion is included in the Contextual Tool to ensure explicit consideration and to prompt identification of the potential environmental impact of the intervention from a qualitative standpoint. If data is available, this criterion could become operationalizable (by defining low and high ends of the scale) and be added to the MCDA Core Model for quantitative assessment. |
| **Feasibility contextual criteria** | | | |
| **System capacity and appropriate use of intervention** Possible sub-criteria:   - *Organizational requirements (*e.g.*, process, premises, equipment)* - *Skill requirements* - *Legislative requirements* - *Surveillance requirements* - *Risk of inappropriate use* - *Institutional limitations to uptake* - *Ability to reach the whole target region/population* | The capacity of a healthcare system to implement the intervention and to ensure its appropriate use depends on its infrastructure, organization, skills, legislation, barriers and risks of inappropriate use. Such considerations include mapping current systems and estimating whether the use of the intervention under scrutiny requires additional capacities. | **In the CONTEXTUAL TOOL**  Would this consideration have a positive, neutral or negative impact on the appraisal of the intervention?  **Positive**: aligned with system capacity and appropriate use  **Negative**: in potential disagreement with system capacity and appropriate use  Consider the capacity of the current system with regard to skills needed, operational structures, regulatory issues to implement intervention and ensure appropriate use.  Example:  *Ease of identifying patients for whom receiving the intervention would be most appropriate*  ***OR***  **In the quantitative MCDA MODEL**  Once critical aspects are defined, they may be added to the MCDA Core Model for quantitative assessment, which will assign more value to interventions aligned with key system aspects. | Ethical foundation: Ensure appropriate use of intervention to **realize its potential benefit and avoid unintended consequences** (aspect of *practical wisdom*).  Rationale for inclusion: Interventions are feasible if they can be used appropriately and preserve the healthcare system’s capacity.  A number of aspects may need to be considered under this umbrella (*e.g.*, skill requirements, organizational requirements). The tool is used here to prompt identification of these aspects. Sub-criteria are proposed to facilitate the process of identification of critical aspects by end-users of the tool. Once identified explicitly, these aspects can become criteria that can either remain in the Contextual Tool for qualitative consideration or, if operationalizable (by defining low and high ends of the scale), be added to the MCDA Core Model for quantitative assessment. |
| **Political, historical & cultural context**  *Possible sub-criteria:*   - *Political priorities and context* - *Cultural acceptability* - *Precedence (congruence with previous and future decisions)* - *Impact on innovation & research* - *Impact on partnership & collaboration among healthcare stakeholders* | The political, historical or cultural context may influence the value of an intervention with respect to specific political situations and overall priorities (*e.g.*, priority for innovation) as well as habits, traditions and precedence. | **In the CONTEXTUAL TOOL**  Would this consideration have a positive, neutral or negative impact on the appraisal of the intervention?  **Positive**: aligned with political, historical & cultural context  **Negative**: in potential disagreement with political, historical & cultural context  Consider whether historical approaches or the political or cultural context directly related to the intervention affect the decision.  *Example:*  *This type of intervention has historically always been considered in a similar fashion and is likely to be considered the same way (or for a new type of intervention, may set a precedent).*  ***OR***  **In the quantitative MCDA MODEL**  Once critical aspects are defined, they may be added to the MCDA Core Model for quantitative assessment to assign more value to interventions aligned with key political/historical/cultural objectives. | Ethical foundation: Awareness of political, historical, cultural aspects ensures that decisions are based on a **broad understanding of the context** (aspect of *practical wisdom*).  Rationale for inclusion: Interventions are feasible if their implementation is supported by the political, historical and cultural context.  This criterion is included in the Contextual Tool to ensure explicit consideration of the political, historical and cultural context, and to prompt the identification of various aspects that may need to be considered, *e.g.*, cultural acceptability, precedence, current political priorities, impact on healthcare research. Sub-criteria are proposed to facilitate the process of identification of critical aspects by users. Once identified explicitly, these may become criteria that can either remain in the Contextual Tool for qualitative consideration or, if operationalizable (by defining low and high ends of the scale), may be added to the MCDA Core Model for quantitative assessment. |

## Consideration of opportunity costs

| **OPPORTUNITY COSTS** | | | |
| --- | --- | --- | --- |
| **Opportunity costs and affordability** *Possible sub-criteria:*   - *Opportunity costs for patient (forgone resources)* - *Opportunity costs for population (forgone resources)* - *Affordability* | Consideration of the medical resources that may be forgone (opportunity costs) if the intervention is implemented and whether the healthcare system can afford implementing the intervention. Both affordability and opportunity cost considerations require a financial/budgeting exercise. Opportunity costs and affordability can be considered at the system/institution level and at the patient level. | Would this consideration have a positive, neutral or negative impact on the appraisal of the intervention?  **Positive**: savings, low opportunity cost  **Negative**: high opportunity cost  Consider what may be lost if the intervention is used/covered and whether the intervention is maximizing impact on health for a given level of resources or whether a health plan/system can afford it.  *Example*:  *Equipment requiring a lot of maintenance and skills to operate may divert hospital resources from basic hospital maintenance; more time with equipment may be less time with the patient.* | Ethical foundation: **Principle of efficiency and awareness** and potential displacements of resources (aspect of *utilitarianism* and aspect of *practical wisdom*).  Rationale for inclusion: Interventions are feasible if they are affordable and associated with a low opportunity cost.  Ensures consideration of what may be forgone if using or reimbursing the intervention (opportunity costs) and whether resources are actually optimized (at patient and societal levels) in doing so. It also includes consideration of affordability.  The actual financial impact of the intervention and available budgets (fixed, expandable *e.g.*, copayment, increased funding) need to be aligned using budgeting tools. Consideration of opportunity costs (forgone interventions), affordability and silo budgeting should be part of this financial exercise.  *Note: the normative aspects of economic considerations [*i.e.*, encouraging savings] are included in the MCDA Core Model* |
