# Supplementary material for: Can reflective multicriteria be the new paradigm for healthcare decision-making? The EVIDEM journey
Source: Cost Eff Resour Alloc. 2018 Nov 9;16(Suppl 1):54. doi: 10.1186/s12962-018-0116-9 (PMC6225552; doi:10.1186/s12962-018-0116-9)
Supplement: Supplementary file 2 — Additional file 2. Adapt and pilot. A step by step directly applicable manual to adapt and pilot the framework in context. [file 12962_2018_116_MOESM2_ESM.docx]

**Additional file 2**

**EVIDEM 10th Edition: Adapt & pilot**

**Table of Contents**

[framework ADAPTATION 6](#_Toc474784314)

[STEP 1: DEFINE OBJECTIVE OF FRAMEWORK 6](#_Toc474784315)

[STEP 2: SELECT AND STRUCTURE CRITERIA 6](#_Toc474784316)

[STEP 3: SELECT METHOD FOR PILOT 11](#_Toc474784317)

[PILOT – STEP A - MULTICRITERIA APPRAISAL 12](#_Toc474784318)

[QUALITATIVE APPROACH 12](#_Toc474784319)

[CORE MODEL 12](#_Toc474784320)

[CONTEXTUAL TOOL 12](#_Toc474784321)

[MIXED-QUALITATIVE - QUANTITATIVE APPROACH 14](#_Toc474784322)

[VALUE SYSTEM ELICITATION (DIRECT RATING SCALE WEIGHTING) - CORE MODEL CRITERIA 14](#_Toc474784323)

[ALTERNATIVE VALUE SYSTEM ELICITATION METHOD: HIERARCHICAL POINT ALLOCATION WEIGHTING 16](#_Toc474784324)

[APPRAISE INTERVENTION 18](#_Toc474784325)

[PILOT – STEP B - VISUALISATION OF REASONING 28](#_Toc474784326)

[MCDA VALUE ESTIMATE 28](#_Toc474784327)

[VISUALISATION DE L’ARGUMENTAIRE 28](#_Toc474784328)

[PILOT – STEP C - 7-RANKING, CONSIDERATION OF OPPORTUNITY COSTS & DELIBERATION 29](#_Toc474784329)

[DECISION/RECOMMENDATION 30](#_Toc474784330)

[Bibliography 31](#_Toc474784331)

[Methodology for synthesized evidence on intervention (EVIDEM Evidence Matrix) 31](#_Toc474784332)

[Analysis of the Quality of Evidence 31](#_Toc474784333)

[Evidence Tables 31](#_Toc474784334)

**IMPORTANT NOTICE - SUMMARY**

**Please read before using EVIDEM**

EVIDEM is a reflective multicriteria approach designed to support the culture of reasonable decision-making by promoting procedural and substantive legitimacy; this includes selection of representative decisionmakers, relevance of reasons for the decision, publicity, appeal, and implementation – based on the ethical framework of accountability of reasonableness (A4R).

To help insure that decisions are based on relevant reasons (substantive legitimacy), EVIDEM provides a set of generic decision criteria derived from the ethical imperatives that underlie the common goal of healthcare and its ultimate motivation: compassion. This represents a generic interpretive frame (MCDA reflective grid) that can be used to elicit individual values and facilitate sharing of diverse perspectives during committee deliberations or for other applications (e.g., patient-clinician shared decisionmaking). These generic criteria can be further concretized to reflect specificities of therapeutic areas or types of interventions. EVIDEM also provides a common structure for all members to express their interpretation of the evidence for each criterion and thereby share their reasoning with others. These interpretations can be expressed quantitatively through interpretive scores (quantitative criteria), qualitatively through impacts (qualitative criteria) as well as narratively through comments (all criteria).

To pursue its objectives, EVIDEM was designed to reduce constraints of the natural decision and deliberation process by ensuring that: all relevant generic criteria are included (whether they are considered qualitatively or quantitatively); scientific and colloquial evidence relevant to each criterion is made available through an efficient synthesis methodology; and face validity is checked at each step of the process (weights, scores and corresponding narratives, aggregated measures) to ensure that visual representations of quantitative outputs reflect the reasoning of individuals or, for system level decisions (e.g., for HTA or MoH), of the committee within and across assessments.

**IMPORTANT NOTICE - DETAILS**

**Please read before using EVIDEM**

Although EVIDEM does use some features of MCDA, its roots are not in the methodology itself but rather in the natural decision and deliberation process. Its goal is to stimulate reflection, deliberation and reasonable decisions rather than algorithms approaches that traditional MCDA tends to promote. Each aspect of its design is geared to support the natural thinking process. It is built to minimize constraints of the natural reasoning in decisionmaking by providing a generic interpretive frame that can be shared across policy committee members, patients and physicians, and healthcare stakeholders at large.

**CRITERIA**

Criteria are selected to support the substantive legitimacy of the decision with regard to the common goal of healthcare systems. The goal is articulated in three normative aspects (ethical imperatives): 1 - alleviate/prevent suffering of patients; 2 – prioritize those who are worst off while ensuring greatest good for greatest number; and 3- ensure sustainability. This is combined with the wisdom of making decisions informed by knowledge and adapted to context (feasibility aspect). These aspects are expressed in 20 criteria in agreement with MCDA methodological principles of non-redundancy, independence, operationalizability and completeness. This creates a generic interpretive frame which, by design, is a reminder of the common goal of healthcare.

THIS SHOULD BE BORNE IN MIND WHEN ADAPATING THE FRAMEWORK BY REMOVING/ADDING GENERIC CRITERIA

*Note 1: Attempts to limit the number of generic criteria for methodological reasons may constrain the reasoning and compromise the integrity of the comprehensive interpretive frame on which EVIDEM is built.*

*Note 2: For each generic criterion, a number of subcriteria are proposed in EVIDEM, which can be broken down further and integrated to reflect specificities of therapeutic areas or types of interventions.*

**REASONING LEADING TO A DECISION**

In the interpretive frame, the narratives and insights of the natural reasoning are structured by criteria and are complemented by qualitative and possibly quantitative outputs and visualization to facilitate their sharing and support the deliberation process.

***Evidence for Healthcare interventions***

When evaluating specific interventions, the goal of evidence synthesis and presentation (scientific and colloquial) is to provide for each criterion the best available and most relevant evidence in a clear format and ensure that the reflection is as unobstructed as possible by irrelevant or biased data.

***Qualitative approach*** The framework can be applied in a qualitative mode that uses the interpretive frame (MCDA grid) to capture interpretations of the available evidence for each criterion in a narrative form and uses implicit weights to arrive at a decision. *A qualitative approach is recommended until a culture of the non-conventional use of numbers that EVIDEM proposes is well established in users.*

WHEN USING THE QUANTITATIVE ASPECTS OF THE FRAMEWORK BEAR IN MIND THET THEY ARE MEANT TO HELP VISUALIZE AND SHARE THE REASONNING

***Mixed-Qualitative - Quantitative approach:***

*Qualitative considerations*

Since some criteria are not suitable for scoring (e.g., cultural and historical context) but nonetheless are an integral part of the reasoning, the framework provides a simple qualitative assessment tool to consider the impact of these criteria (positive, neutral or negative) on the value of interventions.

*Quantitative considerations involve weight and score elicitation and their aggregation.*

*Value system elicitation (Weights) for generic criteria*: Weighting of generic criteria is approached in EVIDEM as a way to explore the value systems (values) of individuals. Since its objective is to stimulate reflection on what matters most to each individual, direct (rather than indirect) weight elicitation methods are proposed, combined with a narrative and face validity exercise to confirm that the weights reflect the value system of the individual

*Preferences (Weights) for specific subcriteria*: subcriteria that are specific to a therapeutic area or type of intervention can be elicited within a generic criterion (e.g., growth hormone: efficacy/effectiveness subcriteria: height [outcome 1], metabolism [outcome 2] etc.); the weights assigned to these represent individual preferences.

*Scores*: Reflective multicriteria analysis encourages the user to reflect on the evidence and make a judgment on its meaning using an interpretive scoring scale and also to provide a narrative to explain the reasoning that underlies the score. (Scores are thus a quantitative representation of an interpretation of the evidence, not a mathematical transformation of data.) These narratives can be summarized for each criterion at the group level for committees’ deliberations. Face validity of the visual representation of the scores is essential to ensure that the scores reflect the reasoning.

*Weights and scores aggregation*: Simple linear aggregation models are applied to create as little mental distance as possible between the measurement and the reasoning. To check face validity, users are presented with a visual representation of the aggregated measurement along with the contribution of each criterion and the associated narratives.

*Modulation by qualitative criteria*: the impact of each qualitative criteria on the aggregated measurement is considered. Face validity is checked at the group level, with a visual representation and associated narratives.

*Last criterion for consideration*:

After the evaluation based on all the other criteria is completed, the criterion “Opportunity cost and financial feasibility” is considered through a budgeting exercise, the committee performs a final deliberation on all aspects brought up and the decision is made.

*Ranking*

As the committee performs multiple assessments, face validity checks are carried out to ensure that the ranking based on the modulated aggregated measures does reflect the group reasoning within and across assessments. Adaptation of the framework is carried out as applicable over time.

THE MATHEMATICAL ASPECTS ARE THUS DESIGNED TO HELP EXPRESS AND SHARE THE REASONING


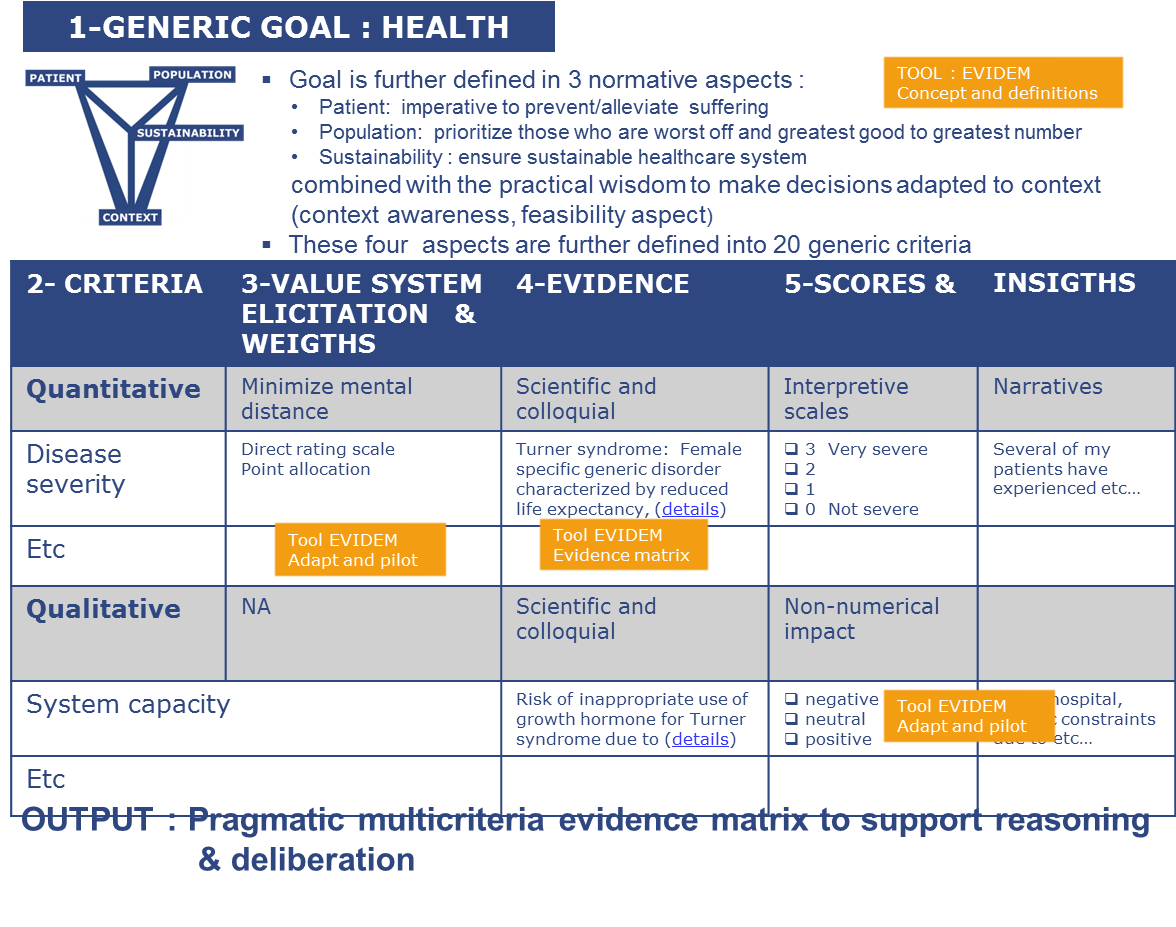

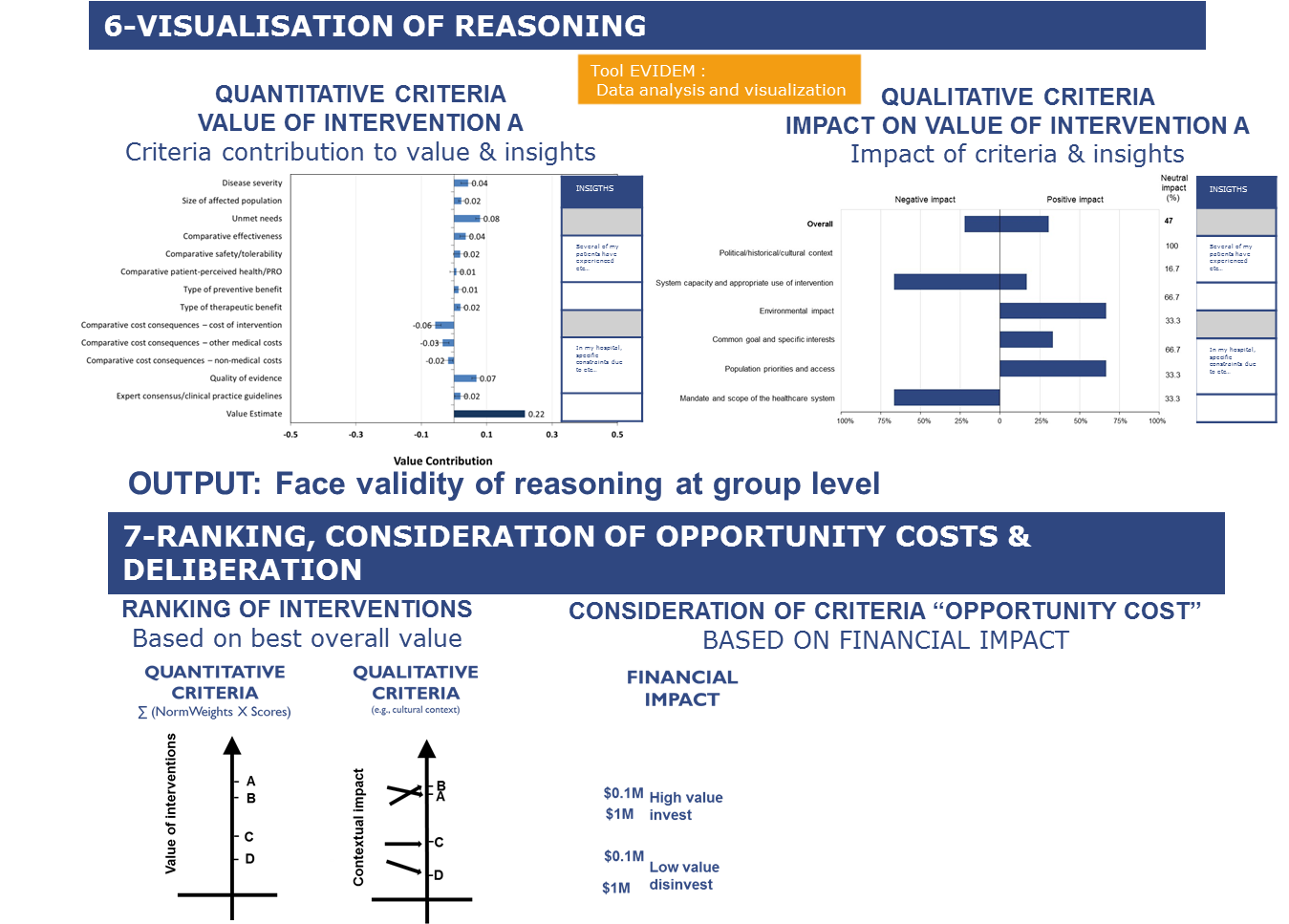


# **framework ADAPTATION**

## STEP 1: DEFINE OBJECTIVE OF FRAMEWORK

## STEP 2: SELECT AND STRUCTURE CRITERIA

**Instructions:** Indicate whether a criterion should or should not be systematically considered when appraising a healthcare intervention

*Note to Chair of session: In adapting the instruments to user context, some of the Contextual Tool criteria can be made quantifiable and added to the EVIDEM Core Model – details on how to consider/develop these contextual criteria are reported in the* **EVIDEM v4.0 Concepts & definitions***.*

*Note to Chair of session: In adapting the instruments to user context, sub-criteria may be added to the framework. To facilitate this process, a number of sub-criteria for each criterion of the EVIDEM Core Model and the Contextual Tool are available in the* **EVIDEM v4.0 Concepts & definitions***.*

| **DOMAINS / Criteria** | ***Possible sub-criteria*** | **Definitions** | **Should be considered?**  ***Delete this column if criteria selection/validation is not part of the workshop*** |
| --- | --- | --- | --- |
| **EVIDEM Core Model (criteria appraised quantitatively)** | | | |
| **NEED FOR INTERVENTION** | | | |
| **Disease severity** | - *Effect of disease on life-expectancy* - *Effect of disease on morbidity (includes disability and function)* - *Effect of disease on patients’ quality of life* - *Effect of disease on caregivers’ quality of life* | Severity of the health condition of patients treated with the proposed intervention (or severity of the health condition that is to be prevented) with respect to mortality, morbidity, disability, function, impact on quality of life, clinical course (i.e., acuteness, clinical stages). | Yes 🞎  No 🞎 |
| **Size of affected population** | - *Prevalence* - *Incidence* | Number of people affected by the condition (treated or prevented by the proposed intervention) among a specified population at a specified time; can be expressed as annual number of new cases (annual incidence) and/or proportion of the population affected at a certain point in time (prevalence). | Yes 🞎  No 🞎 |
| **Unmet needs** | - *Unmet needs in efficacy* - *Unmet needs in safety* - *Unmet needs in patient-reported outcomes* - *Patient demand* | Shortcomings of comparative interventions in their ability to prevent, cure, or ameliorate the condition targeted; also includes shortcomings with respect to safety, patient-reported outcomes and convenience. | Yes 🞎  No 🞎 |
| **COMPARATIVE OUTCOMES OF INTERVENTION** | | | |
| **Comparative effectiveness** | - *Magnitude of health gain* - *Percentage of the target population expected to realize the anticipated health gain* - *Onset and duration of health gain* - *Sub-criteria for the measure of efficacy specific to the therapeutic area* | Capacity of the intervention to prevent or to produce a desired (beneficial) change in signs, symptoms or course of the targeted condition above and beyond beneficial changes produced by alternative interventions. | Yes 🞎  No 🞎 |
| **Comparative safety / tolerability** | - *Adverse events* - *Serious adverse events* - *Fatal adverse events* - *Short-term safety* - *Long-term safety* - *Tolerability* | Capacity of the proposed intervention to produce a reduction in intervention-related harmful or undesired health effects compared to alternative interventions. | Yes 🞎  No 🞎 |
| **Comparative patient-perceived health / patient-reported outcomes** | - *Improvement in health-related quality of life* - *Impact on autonomy* - *Impact on dignity* - *Convenience / ease of use / mode & setting of administration* | Capacity of the intervention to produce beneficial changes in patient-perceived health and patient-reported outcomes (PROs) (e.g., quality of life) above and beyond beneficial changes produced by alternative interventions; also includes improvement in convenience to patients. | Yes 🞎  No 🞎 |
| **TYPE OF BENEFIT OF INTERVENTION** | | | |
| **Type of preventive benefit** |  | Nature of the preventive benefit or risk reduction provided by the proposed intervention at the population-level (e.g., eradication, prevention, reduction in disease transmission, reduction in the prevalence of risk factors). Public health perspective. | Yes 🞎  No 🞎 |
| **Type of therapeutic benefit** |  | Nature of the clinical benefit provided by the intervention at the patient level (e.g., symptom relief, prolonging life, cure). | Yes 🞎  No 🞎 |
| **ECONOMIC CONSEQUENCES OF INTERVENTION** | | | |
| **Comparative cost consequences – cost of intervention** | - *Net cost of intervention* - *Acquisition cost* - *Implementation/ maintenance cost* | Net cost of covering the intervention (excluding other spending). This represents the differential between expected expenditure for the intervention and potential cost savings that may result from replacement of other intervention(s) currently covered by the health plan. Limited to cost of intervention (e.g. acquisition cost, implementation and maintenance cost).  *Note: in countries where part of the intervention cost is paid by patients (e.g. copayment), this criteria should be adjusted consequently*. | Yes 🞎  No 🞎 |
| **Comparative cost consequences – other medical costs** | - *Impact on primary care expenditures* - *Impact on hospital care expenditures* - *Impact on long-term care expenditures* | Impact of the proposed intervention on other medical costs (excluding intervention cost) such as hospitalization, specialist consultations, adverse events costs, long-term care, etc.  *Note: in countries where part of the intervention cost is paid by patients (e.g. copayment), this criteria should be adjusted consequently*. | Yes 🞎  No 🞎 |
| **Comparative cost consequences – non-medical costs** | - *Impact on productivity* - *Financial impact on patients* - *Financial impact on caregivers* - *Costs to the wider social care system* | Impact of the proposed intervention on non-medical costs (excluding intervention cost) such as disability costs, social services, lost productivity, caregiver time, etc. | Yes 🞎  No 🞎 |
| **KNOWLEDGE ABOUT INTERVENTION** | | | |
| **Quality of evidence** | - *Validity* - *Relevance* - *Completeness of reporting* - *Type of evidence* | Extent to which evidence on the intervention is relevant to the decisionmaking body (in terms of population, disease stage, comparator interventions, outcomes, etc.) and valid with respect to scientific standards (i.e., study design, etc.) and conclusions (i.e., agreement of results between studies). This includes consideration of uncertainty (e.g., conflicting results across studies, limited number of studies and patients). Complete reporting of evidence is a pre-requisite to assess coherence and validity. | Yes 🞎  No 🞎 |
| **Expert consensus / clinical practice guidelines** |  | Concurrence of the intervention (or similar alternatives) with the current consensus of experts on what constitutes state-of-the-art practices in the management of the targeted health condition; clinical practice guidelines are usually developed via an explicit process that combines formal and expert knowledge, with the intent to improve clinical practice. | Yes 🞎  No 🞎 |
| **EVIDEM Contextual Tool (criteria appraised qualitatively)** | | | |
| **NORMATIVE CONTEXTUAL CRITERIA** | | | |
| **Mandate and scope of healthcare system** |  | Alignment of the intervention with the mandate/scope of the healthcare system. The goal of healthcare is to maintain normal functioning. Mission and scope of healthcare plans/systems derive from this principle. | Yes 🞎  No 🞎 |
| **Population priorities and access** | - *Current priorities of health system (e.g., disabled; low socioeconomic status; specific age groups)* - *Special populations (e.g., ethnicity)* - *Remote communities* - *Rare diseases* - *Specific therapeutic areas* | Alignment of the intervention with current priorities of health system/plan. Priorities for specific groups of patients are defined by societies/decisionmakers and reflect their moral values. Such considerations are aligned with the principle of justice, which considers treating like cases alike and different cases differently and often gives priority to those who are worst-off. | Yes 🞎  - Add to quantitative appraisal 🞎  No 🞎 |
| **Common goal and specific interests** | - *Stakeholder pressures* - *Stakeholders barriers* - *Conflict of interest* | Pressures or barriers from groups of stakeholders or individuals are often part of the context surrounding healthcare interventions. Being aware of pressures and interests at stake and how they may affect decisionmaking helps ensure that decisions are aligned with the common goal. | Yes 🞎  -  No 🞎 |
| **Environmental impact** | - *Environmental impact of production* - *Environmental impact of use* - *Environmental impact of implementation* | The extent to which the production, use or implementation of the intervention causes environmental damages. | Yes 🞎  - Add to quantitative appraisal 🞎  No 🞎 |
| **FEASIBILITY CONTEXTUAL CRITERIA** | | |  |
| **System capacity and appropriate use of intervention** | - *Organizational requirements (e.g., process, premises, equipment)* - *Skill requirements* - *Legislative requirements* - *Surveillance requirements* - *Risk of inappropriate use* - *Institutional limitations to uptake* - *Ability to reach the whole target region/population* | The capacity of a healthcare system to implement the intervention and to ensure its appropriate use depends on its infrastructure, organization, skills, legislation, barriers and risks of inappropriate use. Such considerations include mapping current systems and estimating whether the use of the intervention under scrutiny requires additional capacities. | Yes 🞎  - Add to quantitative appraisal 🞎  No 🞎 |
| **Political / historical / cultural context** | - *Political priorities and context* - *Cultural acceptability* - *Precedence (congruence with previous and future decisions)* - *Impact on innovation & research* - *Impact on partnership & collaboration among healthcare stakeholders* | The political, historical or cultural context may influence the value of an intervention with respect to specific political situations and overall priorities (e.g., priority for innovation) as well as habits, traditions and precedence. | Yes 🞎  No 🞎 |
| **Opportunity cost** | | | |
| **Opportunity costs and affordability** | - *Opportunity costs for patient (forgone resources)* - *Opportunity costs for population (forgone resources)* - *Affordability* | Consideration of the medical resources that may be forgone (opportunity costs) if the intervention is implemented and whether the healthcare system can afford implementing the intervention. Both affordability and opportunity cost considerations require a financial/budgeting exercise. Opportunity costs and affordability can be considered at the system/institution level and at the patient level. | Yes 🞎  No 🞎 |

**Other criteria to consider?**

- Subcriteria within generic criteria (e.g., specific treatment outcomes, specific instrument features)

**Criteria structure to reorganize?**

- This may be need to align the framework with existing law or processes

**Comments**

## STEP 3: SELECT METHOD FOR PILOT

- Type of approach: qualitative or mixed qualitative-quantitative;
- If mixed approach: select values elicitation (weighting) method (see below under pilote – direct rating scale (least constraints) or hierarchical point allocation, or both, or other)
- Select healthcare intervention(s) to assess

# **PILOT – STEP A - MULTICRITERIA APPRAISAL**

## QUALITATIVE APPROACH

*Paste synthesized evidence for selected intervention in table below –(prepared following instruction under* ***EVIDEM v4.0 Evidence matrix) -*** *can be high level synthesized evidence or detailed as best applicable*

| INTERVENTION DESCRIPTION | *Intervention category (e.g., drug class):*  *Indication (provide name of agency, e.g., EMA):*  *Dosage/Administration:*  *Intervention duration:*  *Comparators:* | | | |
| --- | --- | --- | --- | --- |
| ECONOMIC BURDEN OF DISEASE***Not considered a criterion that contributes to the value of an intervention but provides useful background information. The overall economic burden of the disease is a composite of disease severity, size of affected population and cost of current treatment, all of which are captured in 3 distinct criteria of the quantitative Core Model* |  | | | |
| **DOMAINS / Decision criteria** | | **Synthesized Evidence** | | **Comments from evaluators** |
| CORE MODEL | | | |  |
| **Need for intervention** | | |  |  |
| **Disease severity** | | |  |  |
| **Size of affected population** | | |  |  |
| **Unmet needs** | | |  |  |
| **Comparative outcomes of intervention** | | |  |  |
| **Comparative effectiveness** | | |  |  |
| **Comparative safety / tolerability** | | |  |  |
| **Comparative patient-perceived health / patient-reported outcomes** | | |  |  |
| **Type of benefit of intervention** | | |  |  |
| **Type of preventive benefit** | | |  |  |
| **Type of therapeutic benefit** | | |  |  |
| **Economic consequences of intervention** | | |  |  |
| **Comparative cost consequences – cost of intervention** | | |  |  |
| **Comparative cost consequences – other medical costs** | | |  |  |
| **Comparative cost consequences – non-medical costs** | | |  |  |
| **Knowledge about intervention** | | |  |  |
| **Quality of evidence** | | |  |  |
| **Expert consensus / clinical practice guidelines** | | |  |  |
| CONTEXTUAL TOOL | | |  |  |
| **Normative contextual criteria** | | |  |  |
| **Mandate and scope of healthcare system** | | |  |  |
| **Population priorities and access** | | |  |  |
| **Common goal and specific interests** | | |  |  |
| **Environmental impact** | | |  |  |
| **feasibility contextual criteria** | | |  |  |
| **System capacity and appropriate use of intervention** | | |  |  |
| **Political / historical / cultural context** | | |  |  |
| **opportunity cost** | | |  |  |
| **Opportunity costs and affordability** | | |  |  |

## MIXED-QUALITATIVE - QUANTITATIVE APPROACH

### VALUE SYSTEM ELICITATION (DIRECT RATING SCALE WEIGHTING) - CORE MODEL CRITERIA

This step is performed independently of the intervention to elicit and identify individual value system that underlies individual perspective on decisions

*Note: This is a simple weight elicitation technique with little constraint on the interpretive frame (below point allocation method is also proposed with a hierarchical approach which brings some constraints but might be preferred)*

| **VALUE SYSTEM ELICITATION**  **Weighting Method 1: Direct rating scale** | |
| --- | --- |
| **Instructions**   - From your perspective, assign a weight to each criterion of decision according to its **relative importance** when appraising a healthcare intervention in the context of appraisal (e.g., reimbursement in country X)**.** - Assign 5 to the criteria you consider most important - Assign 1 to the criteria you consider least important   Note*: applicable weights will be normalized*  *Note to Chair of session: define criteria – a brief description is available at the beginning of this document (details available in the* **EVIDEM v4.0 Concepts & definitions***)* | |
| **DOMAINS / Criteria** | **Weight (relative importance)**  **Low High** |
| **Need for intervention** | |
| Disease severity  *What is the relative importance of the severity of the disease targeted by the intervention?* | 1  2  3  4  5 |
| Size of affected population  *What is the relative importance of the size of the population targeted by the intervention?* | 1  2  3  4  5 |
| Unmet needs  *What is the relative importance of the level of unmet needs to manage this disease?* | 1  2  3  4  5 |
| **Comparative outcomes of intervention** | |
| Comparative effectiveness  *What is the relative importance of the efficacy/ effectiveness of an intervention compared to its alternatives?* | 1  2  3  4  5 |
| Comparative safety / tolerability  *What is the relative importance of the safety of an intervention compared to its alternatives?* | 1  2  3  4  5 |
| Comparative patient-perceived health / patient-reported outcomes  *What is the relative importance of the patient-perceived health/ patient-reported outcomes of an intervention compared to its alternatives?* | 1  2  3  4  5 |
| **Type of benefit of intervention** | |
| Type of preventive benefit  *What is the relative importance of the type of preventative health benefit (e.g., risk reduction) provided by an intervention?* | 1  2  3  4  5 |
| Type of therapeutic benefit  *What is the relative importance of the type of therapeutic health benefit provided by an intervention?* | 1  2  3  4  5 |
| **Economic consequences of intervention** | |
| Comparative cost consequences – cost of intervention  *What is the relative importance of the direct cost impact of the intervention (including acquisition, implementation and maintenance costs)?* | 1  2  3  4  5 |
| Comparative cost consequences – other medical costs  *What is the relative importance of the impact of the intervention on other medical costs such as hospitalization, specialist consultations, adverse events costs, long-term care, etc.?* | 1  2  3  4  5 |
| Comparative cost consequences – non-medical costs  *What is the relative importance of the impact of the intervention on non-medical costs such as* *disability costs, social services, lost productivity, caregiver time, etc.?* | 1  2  3  4  5 |
| **Knowledge about intervention** | |
| Quality of evidence  *What is the relative importance of the quality of the design of studies and their relevance to the context?* | 1  2  3  4  5 |
| Expert consensus / clinical practice guidelines  *What is the relative importance of the recommendations on product (or products of the same class) in well-established guidelines?* | 1  2  3  4  5 |
| **Other domains / criteria**  *Note: Adapt MCDA Core Model to specific applications by adding domains / criteria / sub-criteria (see above and see also details on adaptation available in the* **EVIDEM v4.0 Concepts & definitions***)* | |
|  | 1  2  3  4  5 |
|  | 1  2  3  4  5 |

### ALTERNATIVE VALUE SYSTEM ELICITATION METHOD: HIERARCHICAL POINT ALLOCATION WEIGHTING

| **ALTERNATIVE VALUE SYSTEM ELICITATION**  **Weighting Method 2: Direct rating scale** |
| --- |
| **Instructions** Divide 100 points across the domains, and divide 100 points across the criteria within each domain. Assign the points to the domains/criteria according to how much weight (**relative importance**) you think should be attached to that particular domain/criterion.  *Note to Chair of session: Adapt tree to specific applications by adding domains / criteria / sub-criteria (see above and also details on adaptation available in the*  **EVIDEM v4.0 Concepts & definitions***.*  **domains**  **Criteria**  **VALUE OF INTER**  **-**  **VENTION**  **Need for**  **intervention**  Disease severity  Size of affected population  Unmet needs  **Type of benefit of**  **intervention**  Type of preventive benefit  Type of therapeutic benefit  **Comparative**  **outcomes of**  **intervention**  Comparative effectiveness  Comparative safety / tolerability  Comparative patient-perceived health / patient-reported outcomes  **Economic**  **consequences of**  **intervention**  Impact of intervention on other medical  -  Comparative cost consequences –  cost of intervention  **Knowledge about**  **intervention**  Quality of evidence  Expert consensus / Clinical practice  guidelines  ….. pts  ….. pts  ….. pts  ….. pts  ….. pts  100 pts  …… pts  ….. pts  …… pts  100 pts  …… pts  …… pts  100 pts  100 pts  100 pts  100 pts  …… pts  …… pts  …… pts  ….. pts  …… pts  …… pts  ….. pts  …… pts  Comparative cost consequences –  other medical costs  Comparative cost consequences –  non-medical costs |

### APPRAISE INTERVENTION

*Paste synthesized evidence for selected intervention in table below (prepared following instruction under* ***EVIDEM V4.0 Evidence matrix***

| **Intervention:**  **Disease:**  **Setting:**  **Last update**: | |
| --- | --- |
| INTERVENTION DESCRIPTION | *Intervention category (e.g., drug class):*  *Indication (provide name of agency, e.g., EMA):*  *Dosage/Administration:*  *Intervention duration:*  *Comparators:* |
| ECONOMIC BURDEN OF DISEASE***Not considered a criterion that contributes to the value of an intervention but provides useful background information. The overall economic burden of the disease is a composite of disease severity, size of affected population and cost of current treatment, all of which are captured in 3 distinct criteria of the quantitative Core Model* |  |

| **CORE MODEL** | | |
| --- | --- | --- |
| **Instructions**   - For each criterion, assign a score (or range of scores to reflect your uncertainty) based on the evidence available. Comments may be provided. - In case of no data, assign a score or a range of scores reflecting how you usually deal with such situations.   ***Note*:** score ranges will be transformed into a measure of uncertainty.  *Note to Chair of session: provide scoring examples (see details in* **EVIDEM v4.0 Concepts & definitions***).* | | |
| **Criteria** | **Synthesis of available evidence for intervention** | **Scoring intervention & comments** |
| **Need for intervention** | | |
| **Disease severity**  *How severe is the disease targeted by the intervention?* |  | 5 Very severe  4  3  2  1  0 Not severe  Comments |
| **Size of affected population**  *What is the size of the population targeted by the intervention?* | **Prevalence/incidence** | 5 Common disease  4  3  2  1  0 Very rare disease  Comments |
| **Unmet needs**  *Are there many unmet needs to manage this disease with regard to the outcomes of comparative alternative interventions?* | - Efficacy / effectiveness: - Safety / tolerability: - Health-related quality of life (HRQL): - Other: | 5 Many & serious unmet needs  4  3  2  1  0 No unmet needs  Comments |
| **Comparative outcomes of intervention (Extent of Benefit)**  **– see details in Evidence Tables in the Appendix** | | |
| **Comparative effectiveness**  *How does this intervention compare to alternatives with respect to efficacy / effectiveness outcomes?* | **Efficacy data:**  **Comparators included in evaluation:**  **Effictiveness data:** | 5 Much better than comparator  4  3  2  1  0 No difference  -1  -2  -3  -4  -5 Much worse than comparator  Comments |
| **Comparative safety / tolerability**  *How does this intervention compare to alternatives with respect to safety / tolerability outcomes?* | **Common AEs:**  **Serious AEs:**  **Warnings:** | 5 Much better than comparator  4  3  2  1  0 No difference  -1  -2  -3  -4  -5 Much worse than comparator  Comments |
| **Comparative patient-perceived health / patient-reported outcomes**  *How does this intervention compare to alternatives with respect to patient-perceived health / patient-reported outcomes?* | **PATIENT-REPORTED OUTCOMES (PRO)/QUALITY OF LIFE (QOL) DATA**  **RCTs:**  **Observational studies:**  **Patient input:**    **CONVENIENCE** | 5 Much better than comparator  4  3  2  1  0 No difference  -1  -2  -3  -4  -5 Much worse than comparator  Comments |
| **Type of benefit of intervention** | | |
| **Type of preventive benefit**  *What type of preventative health gain or reduction of risk of disease is provided by the intervention?* | **Risk reduction/prevention** | 5 Elimination of multiple diseases  4  3  2  1  0 No reduction in risk of disease    Comments |
| **Type of therapeutic benefit**  *What type of health gain is provided by the intervention?* | **Goal of treatment (e.g., cure, symptom relief)** | 5 Cure / life saving  4  3  2  1  0 No therapeutic benefit  Comments |
| **Economic consequences of intervention** | | |
| **Comparative cost consequences – cost of intervention**  *What is the cost of the intervention (including acquisition, implementation and maintenance costs) compared to other current interventions (comparators)?* |  | 5 Substantial savings  4  3  2  1  0 No change in spending  -1  -2  -3  -4  -5 Substantial additional expenditures  Comments |
| **Comparative cost consequences – other medical costs**  *What is the impact of the intervention on other medical costs such as hospitalization, specialist consultations, adverse events costs, long-term care, etc.?* |  | 5 Substantial savings  4  3  2  1  0 No change in spending  -1  -2  -3  -4  -5 Substantial additional expenditures  Comments |
| **Comparative cost consequences – non-medical costs**  *What is the impact of the intervention on non-medical costs such as disability costs, social services, lost productivity, caregiver time, etc.?* |  | 5 Substantial savings  4  3  2  1  0 No change in spending  -1  -2  -3  -4  -5 Substantial additional expenditures  Comments |
| **Knowledge about intervention**  **– see detailed assessment of quality in Appendix: Analysis of the Quality of Evidence** | | |
| **Quality of evidence**  *What is the quality of the design of studies and their relevance to the context?* |  | 5 Highly relevant and valid  4  3  2  1  0 Not relevant and/or valid  Comments |
| **Expert consensus / clinical practice guidelines**  *Is the intervention (or similar interventioins) recommended in well-established guidelines? What type of recommendation (first line?, Level 1?)* |  | 5 Strong recommendation for intervention above all other alternatives  4  3  2  1  0 Not recommended  Comments |

Appraisal of intervention includes also considerations of contextual criteria, which are structured into the Contextual Tool.

*Note to Chair of session: In adapting the instruments to user context, some of these criteria can be made quantifiable and added to the quantitative EVIDEM Core Model (see details in* **EVIDEM v4.0 Concepts & definitions***.)*

| **CONTEXTUAL TOOL** | | |
| --- | --- | --- |
| **Instructions**   - Based on evidence and your insights, indicate how the consideration of each criterion **impacts** your appraisal of the intervention in the context of your health plan / healthcare system. - You may provide your insights/colloquial evidence regarding the criteria (optional). - Comments may be provided. | | |
| **Criteria** | **Synthesis of available evidence for intervention** | **Impact & comments** |
| **Normative contextual criteria** | | |
| **Mandate and scope of healthcare system** | *Is the intervention in indication aligned with the mandate and scope of your health plan / your country’s healthcare system?*  *What impact does the consideration of this criterion have on the value of the intervention?*  **Evidence**:  **Insights from appraiser**: | Impact  Negative  None  Positive  Comments |
| **Population priorities & access** | *Is the intervention aligned with specific priorities of your health plan / your country’s healthcare system?*  *What impact does the consideration of this criterion have on the value of the intervention?*  **Evidence:**  **Insights from appraiser**: | Impact  Negative  None  Positive  Comments |
| **Common goal and specific interests** | *Are you aware of pressures/barriers from stakeholders regarding the intervention?*  *What impact does the consideration of this criterion have on the value of the intervention?*  **Evidence:**  **Insights from appraiser**: | Impact  Negative  None  Positive  Comments |
| **Environmental impact** | *What are the potential environmental consequences of the intervention?*  *What impact does the consideration of this criterion have on the value of the intervention?*  **Evidence**:  **Insights from appraiser:** | Impact  Negative  None  Positive  Comments |
| **Feasibility contextual criteria** | | |
| **Opportunity costs & affordability** | *Does the intervention result in significant displacement of resources of the healthcare system in your region?*  *What impact does the consideration of this criterion have on the value of the intervention?*  **Evidence:**   - **Annual projected impact of reimbursing intervention on setting/setting spending for indication** (includes intervention and implementation/administration costs, based on origin and type of budget impact model)   **Scenario 1: *Brief description of scenario including availability of comparators***   \|  \| **Number of patients receiving intervention (N_i_)** \| **Average annual cost per patient receiving intervention (C_i_)** \| **Annual intervention cost (T_i_ = N_i_ × C*_i_*_­_)** \| **Total annual cost if intervention is covered  (T_ic_=T_i_ +N_a,ic_ × C_a_ + N_b,ic_×C_b_ + …)** \| **Total annual cost if intervention is not covered  (T_inc_=N_a,inc_×C_a_ + N_b,inc_×C_b_ + …)** \| **Incremental (net) impact of intervention on spending for indication  (T_ic_ -T_inc_)** \| \| --- \| --- \| --- \| --- \| --- \| --- \| --- \| \| Yr 1 \|  \|  \|  \|  \|  \|  \| \| Yr 2 \|  \|  \|  \|  \|  \|  \| \| Yr 3 \|  \|  \|  \|  \|  \|  \| \| Yr 4 \|  \|  \|  \|  \|  \|  \| \| Yr 5 \|  \|  \|  \|  \|  \|  \| \| **Total 5 years** \|  \|  \|  \|  \|  \|  \|   ic: intervention covered; inc: intervention not covered  * †   - Provide explanation on difference between cost to health plan and incremental budget impact - Provide relevant data that may significantly affect estimate (e.g., generics if not included in model) - Report results of sensitivity analyses   **Insights from appraiser**: | Impact  Negative  None  Positive  Comments |
| **System capacity and appropriate use of intervention** | *Does your health plan / your country’s healthcare system have sufficient capacity (e.g., skills, knowledge of intervention, surveillance system) to ensure appropriate use of the intervention?*  *What impact does the consideration of this criterion have on the value of the intervention?* | Impact  Negative  None  Positive  Comments |
| **Political / historical / cultural context** | *Are there any political/historical/cultural factors that may influence the value of this intervention (such as precedence, impact on innovation, impact on collaboration within the healthcare system)?*  *What impact does the consideration of this criterion have on the value of the intervention?*  **Evidence:**  **Insights from appraiser**: | Impact  Negative  None  Positive  Comments |

**Comments**

# **PILOT – STEP B - VISUALISATION OF REASONING**

## MCDA VALUE ESTIMATE

MCDA value estimates are generated by combining normalized weights, elicited independently of intervention, and scores for each criteria (linear model).

| **MCDA value estimate calculation** |
| --- |
| The MCDA value estimate (V) of the intervention is calculated based on a linear additive model as a sum of the value contributions (V*_x_*) [or combined normalized weights (W*_x_*) and standardized scores (S*_x_*)] of all (*n*) criteria of the quantitative EVIDEM Core Model. $\text{V}=\sum_{x=1}^{\text{n}} \text{V}_{x}=\sum_{x=1}^{n} \left(\text{W}_{x}\times\text{S}_{x}\right)$   **Data analysis and presentation** is performed using the EVIDEM Excel software posted on the web site:  ***EVIDEM v4.0 Data analysis and visualisation***. Results may be presented on the spot or after a session. |

## VISUALISATION OF VALUE CONTRIBUTION

| **VISUALISATION OF VALUE CONTRIBUTION** |
| --- |

**Quantitative criteria (for illustration only)** : Value contribution of each criteria to the intervention’s value


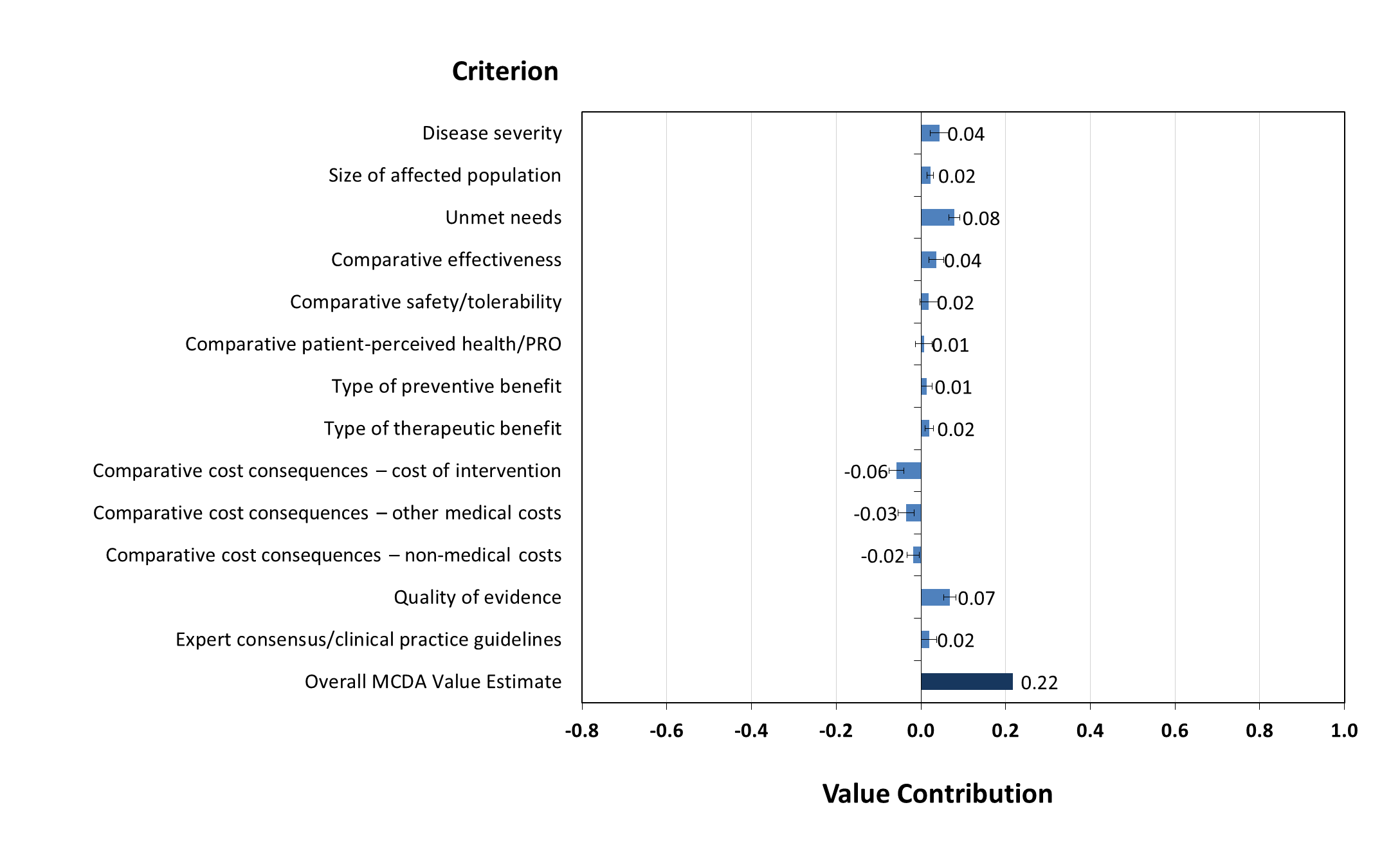


**Qualitative criteria (for illustration only) :** % of members who assigned a positive, negative or neutral impact

100% 75% 50% 25% 0 25% 50% 75% 100%


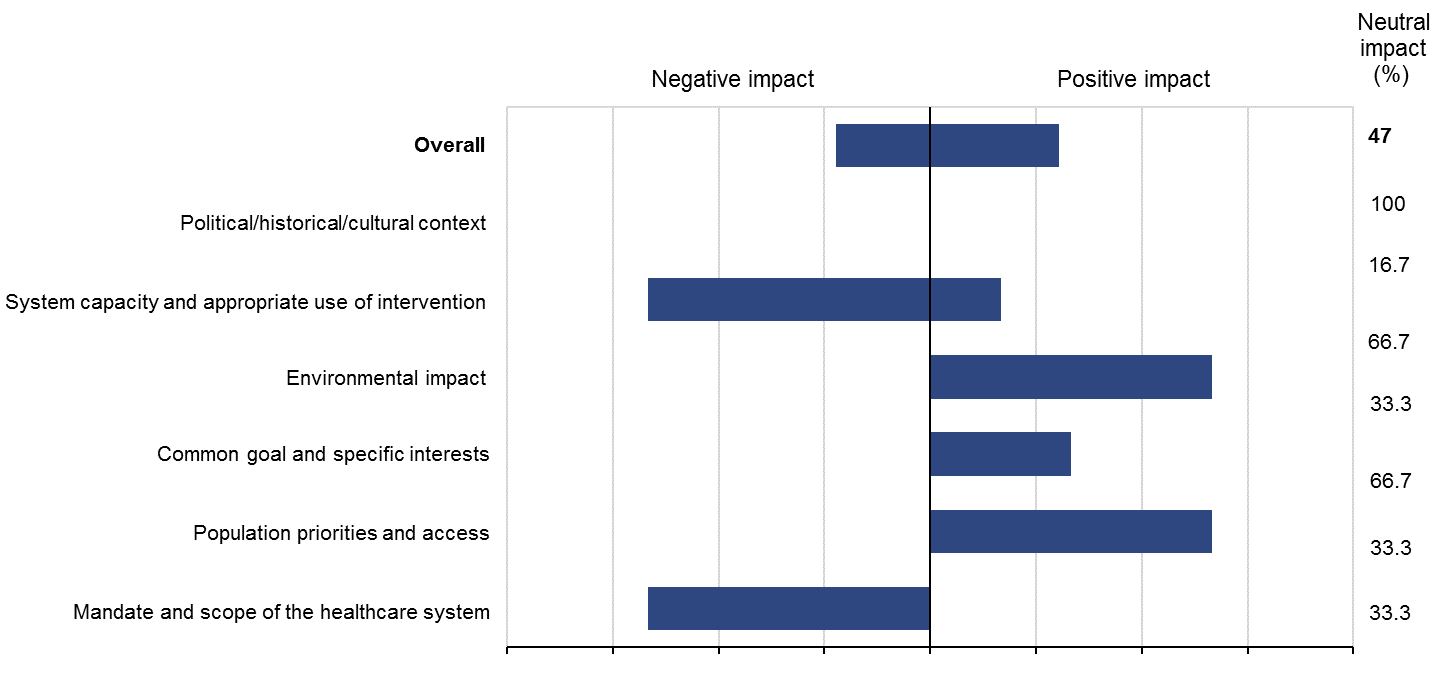


# **PILOT – STEP C - 7-RANKING, CONSIDERATION OF OPPORTUNITY COSTS & DELIBERATION**

*The MCDA value estimate can be used to rank interventions. Ranking based on the quantitative approach can be influenced by contextual qualitative considerations and consideration of opportunity costs.*

The MCDA value scale is rooted in the goal of healthcare, i.e., doing what is best for patients, populations and healthcare systems, which has been translated into the criteria of the framework. The boundaries of the value scale are defined as:

- **Maximum value** (1) represents a hypothetical intervention to prevent and cure severe endemic diseases with significant unmet needs that, compared to existing approaches, has demonstrated large improvements in efficacy, safety and PROs and positive economic consequences.
- M**inimum value** (below 0) represents a hypothetical intervention to relieve minor symptoms for a rare, mild condition with many treatment alternatives that has low-quality data suggesting limited efficacy and major safety and PRO issues as well as major additional spending.

The context of an intervention influence its value, including contextual norms (mandate, priorities), feasibility (capacity, affordability, stakeholders’ barriers & pressures, political/historical context), as represented in figure below. Finally, the opportunity costs is considered using a financial exercise and decision is made guided by identifying the holistic value of interventions, and their value “towards the goal”.


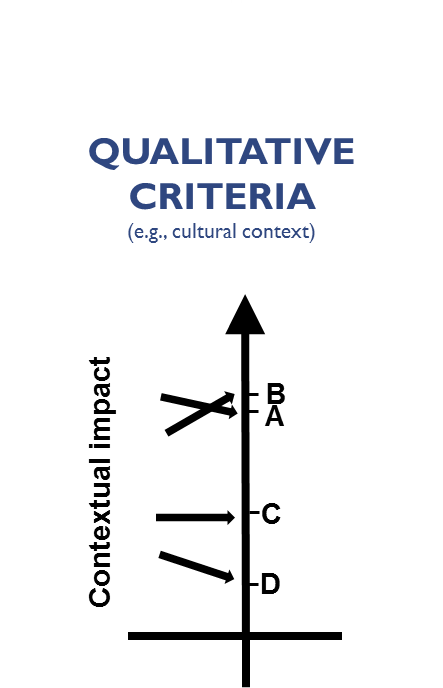

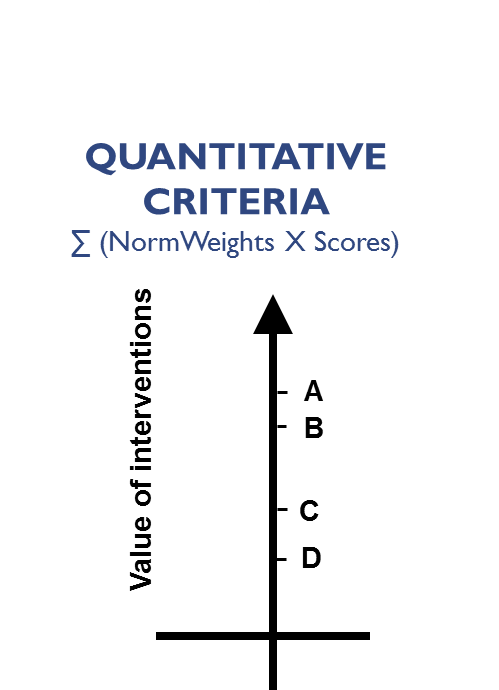

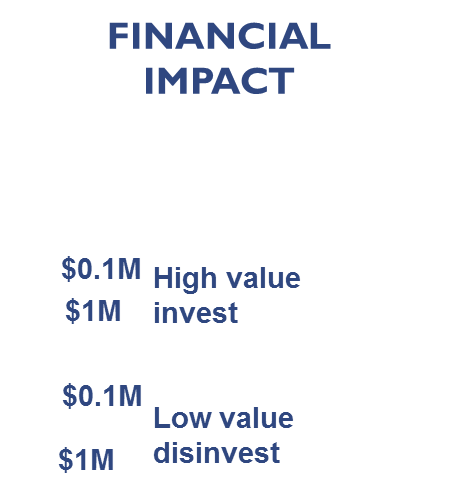


## DECISION/RECOMMENDATION

**Final comments**

**COVERAGE Decision**

*DEFINE OPTIONS of your insitution*

**XX**

YY

# **Bibliography**

*List bibliography cited in synthesized evidence section above*

# **Methodology for synthesized evidence on intervention (EVIDEM Evidence Matrix)**

*Applying this value assessment package requires that the following information is developed for the assessed intervention.*

- *Synthesizing evidence and assessing the quality of evidence for quantitative EVIDEM Model and Contextual Tool (see below)*
- *Review by EVIDEM trained investigator and validation by experts (as applicable)*

*The methodology actually applied should be described here (paste from completed* **EVIDEM v4.0 Evidence matrix** *for selected healthcare interventions)*

# **Analysis of the Quality of Evidence**

*Paste completed quality assessment instruments here*

# **Evidence Tables**

*Paste completed Evidence Tables from the* **EVIDEM v4.0 Evidence matrix** *here.*
