# Supplementary material for: Can reflective multicriteria be the new paradigm for healthcare decision-making? The EVIDEM journey
Source: Cost Eff Resour Alloc. 2018 Nov 9;16(Suppl 1):54. doi: 10.1186/s12962-018-0116-9 (PMC6225552; doi:10.1186/s12962-018-0116-9)
Supplement: Supplementary file 3 — Additional file 3. Evidence matrix, Detailed instructions based on HTA principles to research, synthesize and report evidence for each criterion; instruments to assess quality of evidence. [file 12962_2018_116_MOESM3_ESM.docx]

**Additional file 3**

**EVIDEM 10th Edition: Evidence matrix**

**Instructions for synthesizing evidence and performing quality assessment of studies**

**Table of contents**

[1 Overall EVIDENCE MATRIX SYNTHESIS methodology 8](#_Toc474784467)

[2 Literature review and data collection 8](#_Toc474784468)

[3 Highly Synthesized evidence – executive level 9](#_Toc474784469)

[DOMAINS / Decision criteria 10](#_Toc474784470)

[3.1 CORE MODEL 10](#_Toc474784471)

[3.1.1 NEED FOR INTERVENTION 10](#_Toc474784472)

[Disease severity 10](#_Toc474784473)

[Size of affected population 10](#_Toc474784474)

[Unmet needs 10](#_Toc474784475)

[3.1.2 COMPARATIVE OUTCOMES OF INTERVENTION 10](#_Toc474784476)

[Comparative effectiveness 10](#_Toc474784477)

[Comparative safety / tolerability 10](#_Toc474784478)

[Comparative patient-perceived health / patient-reported outcomes 11](#_Toc474784479)

[3.1.3 TYPE OF BENEFIT OF INTERVENTION 11](#_Toc474784480)

[Type of preventive benefit 11](#_Toc474784481)

[Type of therapeutic benefit 11](#_Toc474784482)

[3.1.4 Economic consequences of intervention 11](#_Toc474784483)

[Comparative cost consequences – cost of intervention 11](#_Toc474784484)

[Comparative cost consequences – other medical costs 11](#_Toc474784485)

[Comparative cost consequences – non-medical costs 11](#_Toc474784486)

[3.1.5 Knowledge about intervention 13](#_Toc474784487)

[Quality of evidence 13](#_Toc474784488)

[Expert consensus / clinical practice guidelines 13](#_Toc474784489)

[3.2 CONTEXTUAL TOOL 13](#_Toc474784490)

[3.2.1 Normative contextual criteria 13](#_Toc474784491)

[Mandate and scope of healthcare system 13](#_Toc474784492)

[Population priorities and access 13](#_Toc474784493)

[Common goal and specific interests 13](#_Toc474784494)

[Environmental impact 13](#_Toc474784495)

[3.2.2 Feasibility contextual criteria 13](#_Toc474784496)

[System capacity and appropriate use of intervention 13](#_Toc474784497)

[Political / historical / cultural context 14](#_Toc474784498)

[3.3 OPPORTUNITY COSTS 14](#_Toc474784499)

[Opportunity costs and affordability 14](#_Toc474784500)

[4 SYNTHESISED EVIDENCE: EVIDENCE MATRIX - REPORT LEVEL 15](#_Toc474784501)

[Criteria 16](#_Toc474784502)

[4.1 CORE MODEL 16](#_Toc474784503)

[4.1.1 Need for intervention 16](#_Toc474784504)

[Disease severity 16](#_Toc474784505)

[Size of affected population 17](#_Toc474784506)

[Unmet needs 18](#_Toc474784507)

[4.1.2 Comparative outcomes of intervention 18](#_Toc474784508)

[Comparative effectiveness 18](#_Toc474784509)

[Comparative safety / tolerability 20](#_Toc474784510)

[Comparative patient-perceived health / patient-reported outcomes 21](#_Toc474784511)

[4.1.3 Type of benefit of intervention 22](#_Toc474784512)

[Type of preventive benefit 22](#_Toc474784513)

[Type of therapeutic benefit 22](#_Toc474784514)

[4.1.4 Economic consequences of intervention 22](#_Toc474784515)

[Comparative cost consequences – cost of intervention 22](#_Toc474784516)

[Comparative cost consequences – other medical costs 24](#_Toc474784517)

[Comparative cost consequences – non-medical costs 24](#_Toc474784518)

[4.1.5 Knowledge about intervention 26](#_Toc474784519)

[Quality of evidence 26](#_Toc474784520)

[Expert consensus / clinical practice guidelines 27](#_Toc474784521)

[Criteria 28](#_Toc474784522)

[4.2 CONTEXTUAL TOOL 28](#_Toc474784523)

[4.2.1 Normative contextual criteria 28](#_Toc474784524)

[Mandate and scope of healthcare system 28](#_Toc474784525)

[Population priorities and access 28](#_Toc474784526)

[Common goal and specific interests 29](#_Toc474784527)

[Environmental impact 29](#_Toc474784528)

[4.2.2 Feasibility contextual criteria 29](#_Toc474784529)

[System capacity and appropriate use of intervention 29](#_Toc474784530)

[Political / historical / cultural context 29](#_Toc474784531)

[4.3 OPPORTUNITY COST 31](#_Toc474784532)

[Opportunity costs and affordability 31](#_Toc474784533)

[5 Reference list 32](#_Toc474784534)

[6 Evidence tables 32](#_Toc474784535)

[7 Quality assessment tools 34](#_Toc474784536)

[7.1 Epidemiological data 34](#_Toc474784537)

[7.1.1 Tool for assessing the quality of single epidemiological study 34](#_Toc474784538)

[7.1.2 Overall assessment tool for epidemiological studies 35](#_Toc474784539)

[7.2 Efficacy, effectiveness and patient-reported outcomes data 36](#_Toc474784540)

[7.2.1 Tool for assessing the quality of a single interventional study 36](#_Toc474784541)

[7.2.2 Tool for assessing the quality of a single observational study 38](#_Toc474784542)

[7.2.3 Tool for assessing the quality of an intervention outcomes modelling study 40](#_Toc474784543)

[7.2.4 Overall assessment tool for efficacy, effectiveness and patient-reported outcomes studies 41](#_Toc474784544)

[7.3 Economic evaluations data 42](#_Toc474784545)

[7.3.1 Tool for assessing the quality of cost-consequence modelling studies 42](#_Toc474784546)

[7.3.2 Tool for assessing the quality of cost-effectiveness modelling studies 44](#_Toc474784547)

[7.3.3 Tool for assessing the quality of budget impact model studies 46](#_Toc474784548)

**IMPORTANT NOTICE - SUMMARY**

**Please read before using EVIDEM**

EVIDEM is a reflective multicriteria approach designed to support the culture of reasonable decision-making by promoting procedural and substantive legitimacy; this includes selection of representative decisionmakers, relevance of reasons for the decision, publicity, appeal, and implementation – based on the ethical framework of accountability of reasonableness (A4R). *Goetghebeur et al. Lancet 17 Jun 2017*

To help insure that decisions are based on relevant reasons (substantive legitimacy), EVIDEM provides a set of generic decision criteria derived from the ethical imperatives that underlie the common goal of healthcare and its ultimate motivation: compassion. This represents a generic interpretive frame (MCDA reflective grid) that can be used to elicit individual values and facilitate sharing of diverse perspectives during committee deliberations or for other applications (e.g., patient-clinician shared decisionmaking). These generic criteria can be further concretized to reflect specificities of therapeutic areas or types of interventions. EVIDEM also provides a common structure for all members to express their interpretation of the evidence for each criterion and thereby share their reasoning with others. These interpretations can be expressed quantitatively through interpretive scores (quantitative criteria), qualitatively through impacts (qualitative criteria) as well as narratively through comments (all criteria).

To pursue its objectives, EVIDEM was designed to reduce constraints of the natural decision and deliberation process by ensuring that: all relevant generic criteria are included (whether they are considered qualitatively or quantitatively); scientific and colloquial evidence relevant to each criterion is made available through an efficient synthesis methodology; and face validity is checked at each step of the process (weights, scores and corresponding narratives, aggregated measures) to ensure that visual representations of quantitative outputs reflect the reasoning of individuals or, for system level decisions (e.g., for HTA or MoH), of the committee within and across assessments.

**IMPORTANT NOTICE - DETAILS**

**Please read before using EVIDEM**

Although EVIDEM does use some features of MCDA, its roots are not in the methodology itself but rather in the natural decision and deliberation process. Its goal is to stimulate reflection, deliberation and reasonable decisions rather than algorithms approaches that traditional MCDA tends to promote. Each aspect of its design is geared to support the natural thinking process. It is built to minimize constraints of the natural reasoning in decisionmaking by providing a generic interpretive frame that can be shared across policy committee members, patients and physicians, and healthcare stakeholders at large.

**CRITERIA**

Criteria are selected to support the substantive legitimacy of the decision with regard to the common goal of healthcare systems. The goal is articulated in three normative aspects (ethical imperatives): 1 - alleviate/prevent suffering of patients; 2 – prioritize those who are worst off while ensuring greatest good for greatest number; and 3- ensure sustainability. This is combined with the wisdom of making decisions informed by knowledge and adapted to context (feasibility aspect). These aspects are expressed in 20 criteria in agreement with MCDA methodological principles of non-redundancy, independence, operationalizability and completeness. This creates a generic interpretive frame which, by design, is a reminder of the common goal of healthcare.

THIS SHOULD BE BORNE IN MIND WHEN ADAPATING THE FRAMEWORK BY REMOVING/ADDING GENERIC CRITERIA

*Note 1: Attempts to limit the number of generic criteria for methodological reasons may constrain the reasoning and compromise the integrity of the comprehensive interpretive frame on which EVIDEM is built.*

*Note 2: For each generic criterion, a number of subcriteria are proposed in EVIDEM, which can be broken down further and integrated to reflect specificities of therapeutic areas or types of interventions.*

**REASONING LEADING TO A DECISION**

In the interpretive frame, the narratives and insights of the natural reasoning are structured by criteria and are complemented by qualitative and possibly quantitative outputs and visualization to facilitate their sharing and support the deliberation process.

***Evidence for Healthcare interventions***

When evaluating specific interventions, the goal of evidence synthesis and presentation (scientific and colloquial) is to provide for each criterion the best available and most relevant evidence in a clear format and ensure that the reflection is as unobstructed as possible by irrelevant or biased data.

***Qualitative approach*** The framework can be applied in a qualitative mode that uses the interpretive frame (MCDA grid) to capture interpretations of the available evidence for each criterion in a narrative form and uses implicit weights to arrive at a decision. *A qualitative approach is recommended until a culture of the non-conventional use of numbers that EVIDEM proposes is well established in users.*

WHEN USING THE QUANTITATIVE ASPECTS OF THE FRAMEWORK BEAR IN MIND THET THEY ARE MEANT TO HELP VISUALIZE AND SHARE THE REASONNING

***Mixed-Qualitative - Quantitative approach:***

*Qualitative considerations*

Since some criteria are not suitable for scoring (e.g., cultural and historical context) but nonetheless are an integral part of the reasoning, the framework provides a simple qualitative assessment tool to consider the impact of these criteria (positive, neutral or negative) on the value of interventions.

*Quantitative considerations involve weight and score elicitation and their aggregation.*

*Value system elicitation (Weights) for generic criteria*: Weighting of generic criteria is approached in EVIDEM as a way to explore the value systems (values) of individuals. Since its objective is to stimulate reflection on what matters most to each individual, direct (rather than indirect) weight elicitation methods are proposed, combined with a narrative and face validity exercise to confirm that the weights reflect the value system of the individual

*Preferences (Weights) for specific subcriteria*: subcriteria that are specific to a therapeutic area or type of intervention can be elicited within a generic criterion (e.g., growth hormone: efficacy/effectiveness subcriteria: height [outcome 1], metabolism [outcome 2] etc.); the weights assigned to these represent individual preferences.

*Scores*: Reflective multicriteria analysis encourages the user to reflect on the evidence and make a judgment on its meaning using an interpretive scoring scale and also to provide a narrative to explain the reasoning that underlies the score. (Scores are thus a quantitative representation of an interpretation of the evidence, not a mathematical transformation of data.) These narratives can be summarized for each criterion at the group level for committees deliberations. Face validity of the visual representation of the scores is essential to ensure that the scores reflect the reasoning.

*Weights and scores aggregation*: Simple linear aggregation models are applied to create as little mental distance as possible between the measurement and the reasoning. To check face validity, users are presented with a visual representation of the aggregated measurement along with the contribution of each criterion and the associated narratives.

*Modulation by qualitative criteria*: the impact of each qualitative criteria on the aggregated measurement is considered. Face validity is checked at the group level, with a visual representation and associated narratives.

*Last criteron for consideration*:

After the evaluation based on all the other criteria is completed, the criterion “Opportunity cost and financial feasibility” is considered through a budgeting exercise, the committee performs a final deliberation on all aspects brought up and the decision is made.

*Ranking*

As the committee performs multiple assessments, face validity checks are carried out to ensure that the ranking based on the modulated aggregated measures does reflect the group reasoning within and across assessments. Adaptation of the framework is carried out as applicable over time.

THE MATHEMATICAL ASPECTS ARE THUS DESIGNED TO HELP EXPRESS AND SHARE THE REASONING


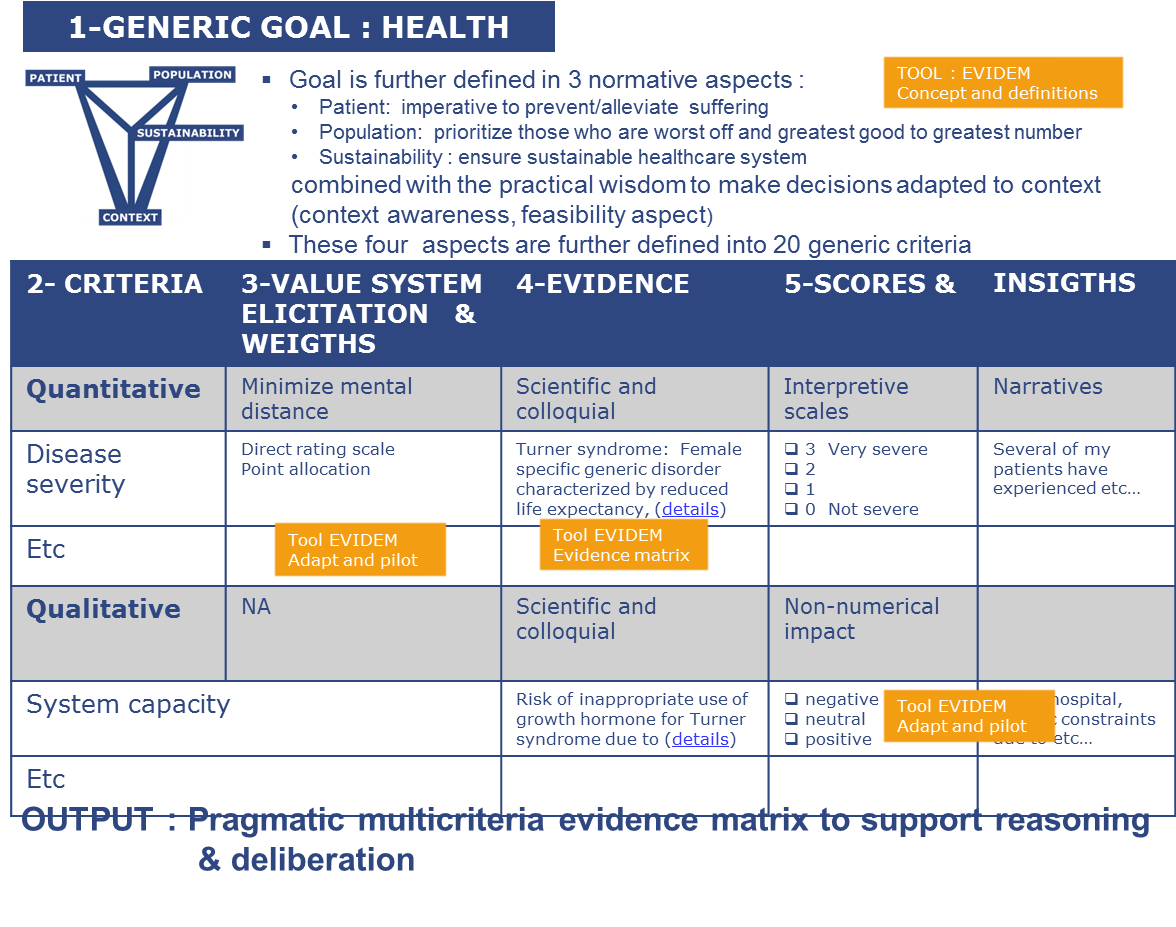

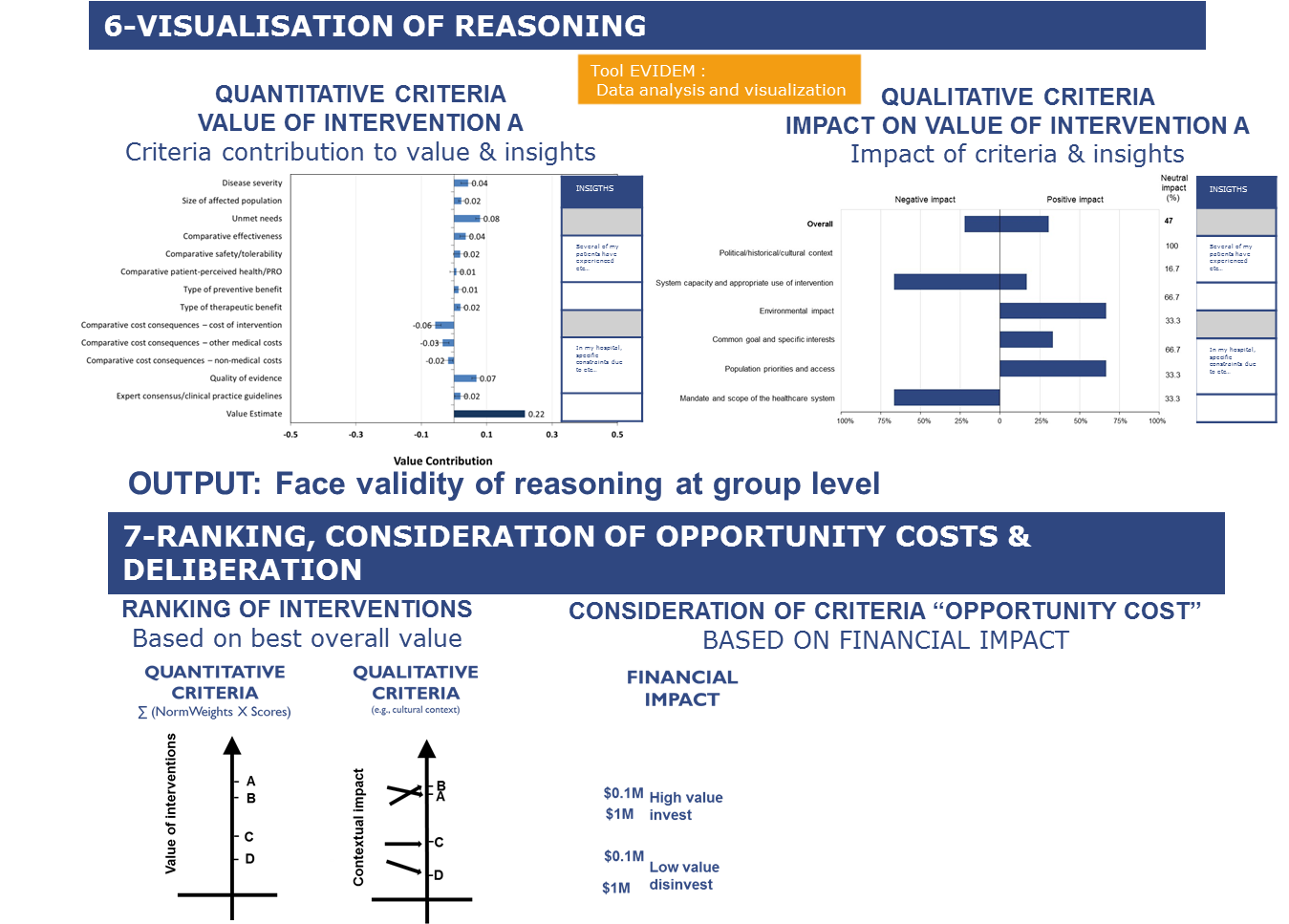


Note: Instructions to prepare synthesized evidence are in red and should be removed when complete

# Overall EVIDENCE MATRIX SYNTHESIS methodology

Once the preparation of the Evidence Matrix using the instructions below is completed, report the methodology used here indicating the following:

- Describe methodology used for identifying and selecting evidence and synthesizing it for the EVIDEM Core Model and EVIDEM Contextual Tool (sources, keywords, selection criteria, data extraction, etc.)
- Indicate which type of evidence was assessed for its quality (clinical, epidemiological, economic, model, etc.) an method used (see section below for EVIDEM tools)
- Indicate the review process and validation by experts, as applicable

# Literature review and data collection

- Perform a literature review and collect unpublished data (as applicable) to identify data required for each criterion
- Tables below may be used to guide the process

**Sources**

| **Source** | **Date Searched** |
| --- | --- |
| **PubMed/Medline,** |  |
| **Embase** |  |
| **Centers for Review and Dissemination** |  |
| **Trial registries** |  |
| **Disease Association web sites** |  |
| **Cochrane reviews** |  |
| **Assessment reports from AHRQ, NICE, CADTH, etc.** |  |
| **Hand searching of bibliographies** |  |
| **Other (please specify)** |  |

**Keywords**

| **Searches** | **Searched terms:** | **No. of records retrieved** |
| --- | --- | --- |
| **Topic: impact on mortality, morbidity and QoL** | |  |
|  |  |  |
|  |  |  |
|  | **Total articles selected** |  |
| **Topic: epidemiology** |  |  |
|  |  |  |
|  |  |  |
|  | **Total articles selected** |  |
| **Topic: clinical guidelines** |  |  |
|  |  |  |
|  |  |  |
|  | **Total articles selected** |  |
| **Topic: current treatment and unmet needs** | | |
|  |  |  |
|  |  |  |
|  |  |  |
|  | **Total articles selected** |  |
| **Topic. Economic context of disease** |  |  |
|  |  |  |
|  |  |  |
|  | **Total articles selected** |  |

# Highly Synthesized evidence – executive level

| INTERVENTION DESCRIPTION | *Intervention category (e.g., drug class):*  *Indication (provide name of agency, e.g., EMA):*  *Dosage/Administration:*  *Intervention duration:*  *Comparators:* |
| --- | --- |
| ECONOMIC BURDEN OF DISEASE* |  |

*Not considered a criterion that contributes to the value of an intervention but provides useful background information. The overall economic burden of the disease is a composite of disease severity, size of affected population and cost of current treatment, all of which are captured in 3 distinct criteria of the quantitative EVIDEM Core Model.

| DOMAINS / Decision criteria | **Highly synthesized evidence** |
| --- | --- |
| CORE MODEL | |
| NEED FOR INTERVENTION |  |
| Disease severity | - Identify, analyze and report necessary and sufficient evidence to understand disease and consequences (description, impact on morbidity, mortality and quality of life) - *Include potentially differentiating aspects of the intervention (e.g., special risks*) - All statements referenced |
| Size of affected population | - Identify, analyze and report size of population affected among a specified population at a specific time and its demographic characteristics - *Include potentially differentiating aspects of the intervention (e.g., sub-population*) - All statements referenced |
| Unmet needs | - Identify, analyze and report the main limitations of the established interventions with respect to efficacy, safety, tolerability, eligible populations, adherence and compliance, patient-reported outcomes (including convenience and patient acceptance) - *Include potentially differentiating aspects of the intervention (e.g., limitations in specific sub-populations*) - All statements referenced |
| COMPARATIVE OUTCOMES OF INTERVENTION | - Describe clinical program (number of trials, design, population, comparators) - Outcomes and their validity |
| Comparative effectiveness | - Identify, analyze and report efficacy and effectiveness data by outcome of interest - For each trial or meta-analysis indicate key information on trial design (randomization, blinding, duration, population of patients – N and description –, interventions compared, primary and secondary efficacy outcomes) and results and significance - *Identify and indicate differentiating aspects between product and comparators regarding efficacy/effectiveness* - All statements referenced   Example: In **XX**, an open-label, multicenter RCT in YY patients inadequately controlled on basal insulin, once-weekly product was superior to comparator with respect to outcome XX (at Week XX: -1.0 kg vs +1.7 kg, *P*<.0001). |
| Comparative safety / tolerability | - Identify and report most common and serious treatment-related adverse events for intervention and key comparator(s) in target population; specify exposure, duration of follow-up, and size of population - *Identify and indicate differentiating aspects between product and comparators regarding safety* - All statements referenced |
| Comparative patient-perceived health / patient-reported outcomes | - Identify, analyze and report patient-reported outcomes data on the intervention and comparator(s), instrument used (specify whether it was validated for the population) and statistical significance of differences - If data not from the same studies covered under effectiveness/efficacy, provide key information on study design - Report patient convenience and adherence data for intervention and comparators - *Identify and indicate differentiating aspects between product and comparators regarding PRO* - All statements referenced |
| TYPE OF BENEFIT OF INTERVENTION |  |
| Type of preventive benefit | - Provide information to identify the nature of the benefit that the intervention can provide to public health, i.e., at the population level (e.g., reduction of risk of disease and mortality, reduction of disease transmission, etc.), including risk reduction rates - *Identify and indicate differentiating aspects between product and comparators* - All statements referenced |
| Type of therapeutic benefit | - Provide information to identify the nature of the benefit that the intervention can provide to individual patients (e.g., cure, prolonging life, or symptom relief) - *Identify and indicate differentiating aspects between product and comparators* - All statements referenced |
| Economic consequences of intervention |  |
| Comparative cost consequences – cost of intervention | - Indicate cost of product - Briefly summarize available data on the differential in expected expenditures for the proposed intervention and the comparators currently covered by the health plan (including acquisition cost, implementation and maintenance cost) - *Identify and indicate differentiating aspects between product and comparators* - All statements referenced |
| Comparative cost consequences – other medical costs | - Identify, analyse and summarize data on the impact of the intervention and comparators on other medical expenditures (excluding intervention cost), such as hospitalization, specialist consultations, adverse events costs, long-term care, as available (data usually derived from the economic criteria above) - Estimate the total impact of intervention and comparator(s) and calculate the absolute difference (increment), if possible - *Identify and indicate differentiating aspects between product and comparators* - All statements referenced |
| Comparative cost consequences – non-medical costs | - Identify, analyse and summarize data on the impact of the intervention and comparators on non-medical expenditures (excluding intervention cost and other medical costs), such as disability costs, social services, lost productivity, caregiver time, as available (data usually derived from the economic criteria above) - Estimate the total impact of intervention and comparator(s) and calculate the absolute difference (increment), if possible - *Identify and indicate differentiating aspects between product and comparators* - All statements referenced |
| Knowledge about intervention |  |
| Quality of evidence | - Report summary of quality assessments for each type of evidence (e.g., clinical, epidemiological, economic as per protocol) - When several studies for one type of evidence (e.g., clinical data), report the global summary covering all studies |
| Expert consensus / clinical practice guidelines | - Identify, analyze and report clinical guideline recommendations for the management of the health condition, including recommendations with respect to the intervention or similar types of intervention to position the intervention within the management of the targeted health condition. - Include potentially differentiating aspects of intervention (e.g., sub-population) - All statements referenced |
| CONTEXTUAL TOOL | |
| Normative contextual criteria |  |
| Mandate and scope of healthcare system | - Provide elements of information to answer the following questions:   *Is the intervention aligned with the mandate/scope of the healthcare system in your country/region? Does the consideration of this criterion have an impact on the value of the intervention?*   - All statements referenced as much as possible; use of grey literature usually required |
| Population priorities and access | - Provide elements of information to answer the following question:   *Does the intervention fall under a defined priority of the healthcare system in your country/region? Does the consideration of this criterion have an impact on the value of the intervention?*   - All statements referenced as much as possible; use of grey literature usually required |
| Common goal and specific interests | - Supplemented by feedback from local stakeholders obtained - Provide elements of information to answer the following questions:   *Are you aware of pressures/barriers from stakeholders regarding this intervention? Does the consideration of this criterion have an impact on the value of the intervention?*   - All statements referenced as much as possible; use of grey literature usually required |
| Environmental impact | - All statements referenced as much as possible; use of grey literature usually required |
| Feasibility contextual criteria |  |
| System capacity and appropriate use of intervention | - Provide high-level synthesis of available data on implementation issues, including the risk of inappropriate use of the intervention and its consequences - Provide elements of information to answer the following questions:   *Does the healthcare system in your country/region have sufficient capacity (e.g., skills, knowledge of intervention, surveillance system) to ensure appropriate use of the intervention? Does the consideration of this criterion have an impact on the value of the intervention?*   - All statements referenced as much as possible; use of grey literature usually required |
| Political / historical / cultural context | - Include legal and social issues for health plan/healthcare system - Provide elements of information to answer the following questions:   *Are you aware of any political/historical/cultural aspect regarding this intervention (such as precedence, impact on innovation, impact on collaboration within the healthcare system)? Does the consideration of this criterion have an impact on the value of the intervention?*   - All statements referenced as much as possible; use of grey literature usually required |
| OPPORTUNITY COSTS | |
| Opportunity costs and affordability | - Provide high-level synthesis of available data on priorities in relevant settings and whether the intervention is targeting priority patient populations or diseases - Provide elements of information to answer the following questions:   *Does the intervention likely result in displacement of resources of the healthcare system in your country/region? Does the consideration of this criterion have an impact on the value of the intervention?*   - All statements referenced as much as possible; use of grey literature usually required |

# SYNTHESISED EVIDENCE: EVIDENCE MATRIX - REPORT LEVEL

**Instructions for developing synthesized evidence on intervention:**

- Analyze, extract and synthesize relevant data in a concise and transparent format (see detailed instructions with examples below – other examples on the Collaborative Registry website)
- Synthesized data should be reviewed by a data synthesis expert and validated by experts in relevant fields (clinical, economic)

Note: Once completed, remove the instructions and example column, and paste the table as an appendix in the ***EVIDEM v4.0 Adapt & pilot***.

| **Disease:**  **Intervention:**  **Setting:** | **Intervention category (e.g., drug class):** where applicable  **Indication *(provide name of agency, e.g., EMA)*:** insert information on approved indication (**source**: e.g., Product Monograph, Technical Sheet, Summary of Product Characteristics)  **Administration/Description:** insert relevant information (e.g., dose and mode of administration for a drug; description of screening test) (**source**: e.g., Product Monograph, Technical Sheet)  **Intervention duration:** where applicable (**source**: Product Monograph, Technical Sheet, clinical trials, practice patterns studies, expert opinion, etc., as available)  **Comparator(s):** list comparators considered (with brief rationale for choice of comparators), and include indication for comparators  **Economic burden of illness*:** Brief overview of direct costs, indirect costs and total costs (as available) associated with the treatment of patients with disease in key countries based on lit review   - Total costs - Direct medical costs of current treatments (e.g., drug costs, costs of administration, follow-up, hospitalization, physician visits, managing adverse events, supportive care, and other relevant costs) - Direct non-medical cost (e.g., patient transportation) - Indirect costs (e.g., productivity loss due to morbidity and pre-mature mortality) - Main cost drivers |
| --- | --- |

*Not considered a criterion that contributes to the value of an intervention but provides useful background information. The overall economic burden of the disease is a composite of disease severity, size of affected population and cost of current treatment, all of which are captured in 3 distinct criteria of the quantitative EVIDEM Core Model.

| Criteria | **Instructions** | **Example – see EVIDEM web site for more examples under the Collaborative Registry**  ***(references were removed for simplicity –***  ***statements should be referenced with access to full text sources)*** |
| --- | --- | --- |
| CORE MODEL | | |
| Need for intervention | | |
| Disease severity *How severe is the disease targeted by the intervention?*  ***Scoring scale****: 0: Not severe →5: Very severe*  *Possible sub-criteria:*   - *Effect of disease on life-expectancy* - *Effect of disease on morbidity (includes disability and function)* - *Effect of disease on patients’ quality of life* - *Effect of disease on caregivers’ quality of life* | **Sources**:   - Literature (*e.g.*, peer-review articles; if many studies on one subject, select recent studies with large representative populations, recent review in prominent journal, textbook such as Harrison’s)   **Content:** Essential information needed to understand the disease and its consequences, as well as to clarify the concepts/outcomes of clinical/PRO data.   - Describe disease (*e.g.*, disease definition, symptoms, etiology, clinical presentation, comorbidities & associated risks) and disease progression - Report impact of disease on mortality, morbidity, quality of life - Where applicable, describe stages or subtypes of disease that differentiate interventions, time course and target populations - **Don’t** include information on epidemiology ; this is covered under Size of population affected - **Don’t** include description of current treatment practice; this is covered under Unmet needs | At early stages of cervical cancer, when the tumor is limited to the cervix, the disease remains unnoticed. Symptoms appear when the cancer spreads to the vagina, the urinary bladder, or the rectum, and include abnormal vaginal bleeding, post-coital spotting, vaginal discharge, and pelvic or low back pain radiating to the lower extremities. At late stages, patients may experience severe backache, severe anemia, weight loss and uncontrolled release of urine and feces through the vagina.  Without adequate treatment, 50% of patients survive less than 18 months from the start of symptoms and only 3.1% survive more than five years. With adequate treatment, 5-year survival depends on the stage of the disease at diagnosis, ranging between close to 100% for stage IA disease (micro-invasive carcinoma), 30% to 50% for stage III disease (involvement of pelvic wall or lower third of vagina) and 5% to 15% for stage IV disease (involvement of bladder or rectum; distant metastasis). |
| Size of affected population *What is the size of the population targeted by the intervention?*  ***Scoring scale****: 0: Very rare disease →5: Common disease*  *Possible sub-criteria:*   - *Prevalence* - *Incidence* | **Sources**:   - Literature (*e.g.*, peer-reviewed studies, national statistics, disease associations, recent reviews) - Give priority to peer-reviewed epidemiological studies and recent reviews based on these; if not available, consult national statistics, surveys and other non-peer-reviewed sources (*e.g.*, databases)   **Content:** Size of the population affected by disease and its demographic characteristics (*i.e.*, age, gender and ethnicity)   - Report worldwide and local prevalence /incidence focusing on key markets; annual number of cases (if not available, use data from a comparable country/population) - Describe epidemiological data that differentiate the intervention from comparators (*e.g.*, special populations, such as those with comorbidities) - Describe briefly study design (*e.g.*, setting, number of individuals) - **Don’t** include information on proportion of patients receiving various interventions; this is covered under **Unmet needs** | Based on pooled data from cytogenetic studies from Canada, Japan, Russia, Denmark and Scotland that included a total of 48,744 newborn females, it was estimated that 50 out of 100,000 females are born with Turner syndrome (1 out of 2000 newborn females). |
| Unmet needs *Are there many unmet needs to manage this disease with regard to the outcomes of comparative alternative interventions?*  *Scoring scale: 0: No unmet needs →5: Many & serious unmet needs*  *Possible sub-criteria:*   - *Unmet needs in efficacy* - *Unmet needs in safety* - *Unmet needs in patient-reported outcomes* - *Patient demand* | **Sources**:   - Literature (*e.g.*, peer-reviewed studies, clinical practice guidelines [CPGs], reviews in prominent journals, Cochrane reviews) - Give priority to recent reviews by prominent researchers and evidence-based CPGs as well as recent studies with large representative populations   **Content:** Information on current practice and limitations in efficacy, safety, and patient-reported outcomes of current standard of care / comparators   - List main limitations of current interventions / comparators in country / setting with respect to efficacy, safety, patient-reported outcomes, proportion of patients eligible for treatment, adherence, convenience, patient acceptance, etc. - Report, if available, proportion of eligible patients currently treated - **Don’t** report limitations related to cost (covered under domain **Economic consequences of intervention**) - **Don’t** comment on whether proposed intervention can address limitations or compare proposed intervention with current interventions; this is covered under the domain *Comparative outcomes of intervention* | Currently, screening for cervical cancer is performed using conventional cytology (pap smear).  **Limitations of conventional cytology pap smear testing include:**   - Relatively low sensitivity (74%): this means that 26% of positive samples are misclassified as negative - Specificity (87%): 13% of negative samples are misclassified as positive (potentially impacting patient’s quality of life) - About 3% of samples are unsatisfactory (cannot be examined with accuracy) thus requiring patient recall to repeat the screening test - Low screening uptake (low adoption of screening programs): In South Africa, pap smears were taken in 4.1% of the female population aged 15 to 65 years during 2002. |
| Comparative outcomes of intervention | | |
| Comparative effectiveness *How does this intervention compare to alternatives with respect to efficacy / effectiveness outcomes?*  ***Scoring scale****: -5: much worse than comparator (negative contribution)→5: much better than comparator(positive contribution)*  *Possible sub-criteria:*   - *Magnitude of health gain* - *Percentage of the target population expected to realize the anticipated health gain* - *Onset and duration of health gain* - *Sub-criteria for the measure of efficacy specific to the therapeutic area* | **Sources**:   - Peer-reviewed studies (randomized controlled trials [RCTs], meta-analyses, observational studies), trial registries - Unpublished data/reports (*e.g.*, from manufacturer) - Give priority to published peer-reviewed comparative RCTs   **Content:** Efficacy and effectiveness data by outcome of interest. Key information on trial design to allow understanding of the trial and outcomes to identify differences between interventions   - Use the generic evidence table at the end of this document to organize clinical trial data before summarizing it - Summarize pivotal trial(s) data; if many trials, select large head-to-head RCTs using comparators most relevant to the setting:   - Include brief description of trial(s) (type of study, intervention, duration of treatment, number of patients, patient inclusion/ exclusion criteria, type of analysis, patient disposition)   - Report the most synthesized and most relevant efficacy data (i.e., most significant/ standard outcome measure; principal analysis) in a format that is most readable (*e.g.*, use percentages instead of fractions; in tables, keep percentage signs with numbers; round numbers keeping only significant digits); report absolute data rather than relative if baseline is comparable; indicate statistical significance - When multiple publications are on the same patient population, report data by cohort not by publication (avoid double reporting of outcomes in the same population) - If available, report key findings of meta-analyses, effectiveness and observational studies - If available, report key findings of modeling studies - If only placebo-controlled trials are available, provide summaries of pivotal comparator trials - Where applicable, indicate differences in population eligibility between proposed intervention and comparators | **EFFICACY DATA**  **Meta-analysis of 2 pivotal trials (prospective, randomized controlled trials; treatment-naïve patients; 24 months, Intention-to-treat analysis [ITT])**   - Trial 1: Treatment Y vs comparator 1 vs placebo - Trial 2: Comparator 1 vs comparator 2   Inclusion criteria**:** Age > 50 years; all types of patients for treatment Y, comparator 1 and placebo; only patients with specific subtype of eye disease X for comparator 2 (comparator 2 indication)  Exclusion criteria: previous treatment/intervention for eye disease X; liver disease; eye surgery within last 2 months  **Primary outcome at 24 months:** Mean change in visual acuity, number of letters (*standard outcome measure for eye disease X; a loss of 45 letters would be defined as legal blindness*)   \|  \| **Treatment Y** \| **Comp. 1** \| **Comp. 2** \| **Placebo** \| \| --- \| --- \| --- \| --- \| --- \| \| All types of disease X \| -7* (n=200) \| -6* (n=300) \| NA \| -15 (n=200) \| \| Subtype only \| -6^†^  (n=100) \| -6^†^  (n=200) \| -8^†^  (n=100) \| -16  (n=100) \|   *Significant differences against placebo*: ^*^*P*<.01, ^†^*P*<.05   - Patient characteristics comparable across groups: mean age 62 years, 45% women, 56% with comorbidity, 38% smokers, 59% retired - Subgroup analyses showed that benefit decreases with age for all treatments; no differences observed between men and women - Patient disposition: approximately 90% of randomized patients completed the studies   **Population of patients eligible for treatment**  Treatment Y and comparator 1 are effective for all types of eye disease X; comparator 2 is effective only in one subtype of eye disease X.  **effectiveness DATA**  Not available |
| Comparative safety / tolerability *How does this intervention compare to alternatives with respect to safety / tolerability outcomes?*  *Scoring scale: -5: much worse than comparator (negative contribution)→5: much better than comparator(positive contribution)*  *Possible sub-criteria:*   - *Adverse events* - *Serious adverse events* - *Fatal adverse events* - *Short-term safety* - *Long-term safety* - *Tolerability* | **Sources**:   - Peer-review studies (RCTs, observational studies, case reports) - Product monographs, technical sheets, surveillance & clinical databases (*e.g.*, from regulatory authorities) - Integrated safety analyses - Unpublished data /reports (*e.g.*, from manufacturer)   **Content:** Potential harms (adverse events [AEs], warnings) related to the use of the intervention and its comparators.   - Report AEs in tabular format (all-cause as well as treatment-related) for intervention and key comparator(s) in target population; specify exposure, duration of follow-up and size of population - If all-cause AEs list is very long, report only AEs with rates above a specific cut-off (report cut-off used) - Report for intervention and comparators: incidence of serious AEs & deaths, discontinuation due to AEs, warnings, and monitoring requirements - Present data for intervention and on comparators from head-to-head trials as available; otherwise from published studies or prescribing information - Identify and indicate differentiating aspects between intervention and comparators regarding safety | **SAFETY DATA**  **Based on meta-analysis of pivotal trials (see details on trials under *Comparative effectiveness*)**  **Incidence of adverse events (AEs)**: 24-month follow-up   \|  \| **Treatment Y**  **N=200** \| **Comp. 1**  **N=300** \| \| \| **Comp. 2**  **N=100** \| **Placebo**  **N=200** \| \| --- \| --- \| --- \| --- \| --- \| --- \| --- \| \| **Common AEs,** % of patients with all-cause AEs (treatment-related AEs) \| \| \| \| \| \| \| \| Eye pain \| 15 (1) \| \| 34 (9) \| 22 (4) \| \| 9 (0) \| \| Eye inflammation \| 6 (0.5) \| \| 17 (4) \| NA \| \| 4 (0.1) \| \| Vitreous opacities \| 8 (0) \| \| 22 (3) \| NA \| \| 7 (0) \| \| Photosensitivity \| 4 (0) \| \| 2 (0) \| 11 (2.5) \| \| 3 (0) \| \| Injection site AEs \| NA \| \| NA \| 12 (9) \| \| NA \| \| **Treatment related serious AEs ,** % of patients \| \| \| \| \| \| \| \| Endophthalmitis \| 0 \| \| 1 \| NA \| \| NA \| \| Vitreous hemorrhage \| 0 \| \| 2 \| NA \| \| NA \| \| Traumatic cataract \| 0 \| \| 1 \| NA \| \| NA \| \| **Discontinuation due to AEs,** % of patients \| \| \| \| \| \| \| \|  \| 1 \| \| 1 \| 4 \| \| 0 \|   **WARNINGS** (***from product monograph***)  ***Treatment Y***: hypertension; proteinurea; renal failure; cardiac failure; hepatotoxicity  ***Comparator 1***: hypersensitivity; haemorrhage; hypocalcemia;; dermatological toxicity; hypertension  MONITORING (*from product monograph*)  ***Treatment Y***: regularly monitor blood pressure, urine protein, clinical symptoms or signs of cardiac decompensation  ***Comparator 1***: regularly monitor blood pressure, electrocardiograms and electrolytes and liver function |
| Comparative patient-perceived health / patient-reported outcomes *How does this intervention compare to alternatives with respect to patient-perceived health / patient-reported outcomes?*  ***Scoring scale****: -5: much worse than comparator (negative contribution)→5: much better than comparator(positive contribution)*  *Possible sub-criteria:*   - *Improvement in health-related quality of life* - *Impact on autonomy* - *Impact on dignity* - *Convenience / ease of use / mode & setting of administration* | **Sources**:   - Peer-reviewed, published studies, registries - Unpublished data, reports, surveys (*e.g.*, from manufacturer) - Patient input   **Content:** Patient-reported outcomes (PRO) data related to the use of the intervention and its comparators.   - Report PRO data for intervention and comparator(s) in a tabular format: population, instrument used, outcomes reported, duration of treatment - Specify if instrument used was validated for population - Report patient convenience & adherence data for intervention and comparators - Provide synthesized patient input (as available) - **Don’t** include convenience for healthcare provider or adherence to healthcare provider guidelines (only patient perspective covered here) | **PATIENT-REPORTED OUTCOMES**:  **Outcome at 24 months**: change from baseline in perceived quality of life using a generic instrument scoring quality of life between 1 (*major problems*) and 5 (*perfect health*)   \|  \| **Treatment Y**  **N=200** \| **Comp. 1**  **N=300** \| **Comp. 2**  **N=100** \| **Placebo**  **N=200** \| \| --- \| --- \| --- \| --- \| --- \| \| **Scores at baseline** \| 3.2 \| 3.3 \| 3.4 \| 3.1 \| \| **Change from baseline at 24 months** \| -0.2 \| -0.4 \| -0.6 \| -1.2 \|   **Convenience**:   - **Treatment Y**: self-administered drops daily - **Comparator 1**: intravitreous injection (into the eyeball) every 6 weeks by trained specialist - **Comparator 2**: intravenous infusion of drug A (over 10 min) followed, 15 minutes later, by laser light administration administered by a trained specialist; avoid exposure of skin or eyes to direct sunlight or bright indoor light for 2 days; patient should be reevaluated every 3 months   **Adherence**: *No data on adherence – Proxy measure:*   - **Comparator 1:** 90% of patients in each treatment arm completed the 24 months - **Comparator 2**: 85% completed 24-month examination |

| Type of benefit of intervention | | |
| --- | --- | --- |
| Type of preventive benefit *What type of preventive health benefit or reduction of risk of disease is provided by the intervention?*  ***Scoring scale:*** *0: No preventive benefit →5: Eradication* | **Sources**:   - Peer-reviewed, published studies; see also criterion *Comparative effectiveness*   **Content:** Nature of the benefit of the intervention at the population level   - Report risk reduction rates resulting from intervention - Provide information to identify the nature of the benefit the proposed intervention can provide to public health, i.e., the population level (*e.g.*, reduction of mortality, reduction of disease transmission) - **Don’t** compare proposed intervention with comparators | Intervention X is expected to reduce the age-standardized incidence of disease X in population Y by over 50%. |
| Type of therapeutic benefit *What type of therapeutic benefit is provided by the intervention?*  ***Scoring scale:*** *0: No therapeutic benefit→5: Cure* | **Sources**:   - Peer-reviewed, published studies; see also criterion *Comparative effectiveness*   **Content**: Nature of the benefit of the intervention at the patient level.   - Provide information to identify the nature of the benefit that the proposed intervention can provide to individual patients (*e.g.*, cure, prolonging life, symptom relief) - **Don’t** compare proposed intervention with comparators | The ultimate goal of growth hormone treatment is to promote growth and improve psychosocial wellbeing of short stature patients with Turner syndrome.   - Efficacy data demonstrates that GH treatment in patient with Turner syndrome for 5–7 years promotes height gain of approximately 7 cm over untreated (see *Comparative effectiveness* criterion) - Patient-reported outcomes data is limited and non-conclusive (see *Comparative patient-perceived health/patient-reported outcomes*) |
| Economic consequences of intervention | | |
| Comparative cost consequences – cost of intervention *What is the cost of the intervention (including acquisition, implementation and maintenance costs) compared to other current interventions (comparators)?*  ***Scoring scale:*** *-5: substantial additional expenditures (negative contribution)→5: substantial savings(positive contribution)*  *Possible sub-criteria:*   - *Net cost of intervention* - *Acquisition cost* - *Implementation/ maintenance cost* | **Sources**:   - Peer-reviewed, published studies - Unpublished data (*e.g.*, from health plan or manufacturer) - Cost data, *e.g.*, from databases   **Content:** Capture the extent of additional or reduced expenditures per patient stemming from covering the intervention.   - Tabulate data on cost per patient of implementing the intervention and comparators (unit cost, frequency/duration of treatment, equipment associated costs, administration cost, annual estimate, etc.). Provide relevant data that may significantly affect estimate (*e.g.*, generics if not included).   **Intervention price, frequency and/or duration of administration and cost of administration *Note: the table below is most applicable for drug products-it needs to be adapted for other types of interventions***   \|  \| **Inter-vention** \| **Compa-rator 1** \| **Compa-rator 2** \| \| --- \| --- \| --- \| --- \| \| **Price per unit (e.g., available dosing form)** \|  \|  \|  \| \| **Recommended daily dose (e.g., from SmPC)** \|  \|  \|  \| \| **Actual daily dose†** \|  \|  \|  \| \| **Average daily drug cost per patient (based on actual dose)** \|  \|  \|  \| \| **Average monthly/annual drug cost per patient** \|  \|  \|  \| \| **Administration cost per month‡** \|  \|  \|  \| \| **Average total monthly/or annual cost per patient** \|  \|  \|  \| \| **Average duration of treatment §** \|  \|  \|  \| \| **Average total cost per treatment course** \|  \|  \|  \|   NA: not applicable  Assumptions and sources:  *  †  ‡  ^§^ | **Intervention price, frequency and/or duration of administration and cost of administration per patient**   \|  \| **Intervention** \| **Comparator a** \| **Comparator b** \| \| --- \| --- \| --- \| --- \| \| **Dose and frequency of administration*** \| 20 mg  2 × daily* \| 30 mg  3 × daily \| 20 U/kg/every week \| \| **Average price per unit sold** \| $7,000 for 60  20-mg capsules \| $5,000 for 90  30-mg capsules \| $200 for  200-U vial \| \| **Average weight of patients** \| Not applicable \| \| 65 kg† \| \| **Cost per treatment** \| $233 per day \| $167 per day \| $1,400 per infusion‡ \| \| **Average number of treatments per year** \| 329 days^§^ \| \| 47 infusions^§^ \| \| **Average annual pharmaceutical cost** \| **$76,767** \| **$54,833** \| **$65,800** \| \| **Average annual cost of administration** \| 0 \| \| $5,000^¶^ \| \| **Average annual therapy cost per patient** \| **$76,767** \| **$54,833** \| **$70,800** \|   * Recommended dose based on SmPC  † Based on mean weight of patients in study XX  ‡ Assuming that no drug is wasted and that unused contents of opened vials are discarded  ^§^ Assuming 90% adherence  ^¶^ IV infusion settings: 40% home, 60% hospital |
| Comparative cost consequences – other medical costs *What is the impact of the intervention on other medical costs such as hospitalization, specialist consultations, adverse events costs, long-term care, etc.?*  ***Scoring scale:*** *-5: substantial additional expenditures (negative contribution)→5: substantial savings(positive contribution)*  *Possible sub-criteria:*   - *Impact on primary care expenditures* - *Impact on hospital care expenditures* - *Impact on long-term care expenditures* | **Sources**:   - Peer-reviewed, published studies; economic evaluations - Unpublished data (*e.g.*, from health plan or manufacturer) - Data often derived from economic evaluations   **Content:** Capture the impact of providing coverage for the proposed intervention on other medical costs (excluding cost of intervention and its comparators)   - Tabulate impact of intervention and comparators on other medical costs, such as hospitalization, specialist consultations, adverse events costs, long-term care, as available. - Estimate total impact of intervention and comparator(s) and calculate the absolute difference (increment), if possible - If no savings with intervention, explain why; if intervention results in net savings, explain why (if not obvious from the table) - **Don’t** report on cost of implementing intervention or comparators (*e.g.*, acquisition cost for a drug, equipment associated cost); covered under *Comparative cost consequences – Cost of intervention*) - **Don’t** include potential for future cost increases for the intervention; this should be covered under *Comparative cost consequences – cost of intervention* - **Don’t** include data on non-medical costs of intervention (covered in *Comparative cost consequences – non-medical costs)* | **Study description**: indicate characteristics of the study from which the data presented is obtained including as applicable: Population; description of intervention (dose, duration) and comparators; model type, time horizon & key model features and assumptions including discounting; Costs included (except cost of intervention itself – see criteria above) (e.g., hospital costs, physician visits, adverse events, etc and how costs were obtained e.g. based on studies, expert opinion, assumptions); sensitivity analyses (on which data, based on which assumptions)  **Study results:**  **Example**  **Impact of treatment Y on other medical expenditures (excluding drug cost, see *Comparative cost consequences – cost of intervention*)**  Includes hospitalization costs, adverse events costs and nursing home costs, based on data available in economic evaluation  **Mean costs (CAD$) per patient:** at 24 months   \|  \| **Treatment costs*** \| **Adverse event costs^†^** \| **Costs of nursing home** \| **Total** \| **Treatment Y savings** \| \| --- \| --- \| --- \| --- \| --- \| --- \| \| **Treatment Y** \| 600 \| 2 \| 5,000 \| 5,602 \| - \| \| **Comp 1** \| 3,000 \| 20 \| 4,500 \| 7,524 \| 1,922 \| \| **Comp 2** \| 2,900 \| 10 \| 5,500 \| 8,408 \| 2,806 \| \| **No treatment** \| 300 \| 1 \| 8,000 \| 8,301 \| 2,699 \|   *includes physician visits, treatment administration and required tests; excludes drug cost  ^†^includes endophthalmitis, vitreous hemorrhage, traumatic cataract, intravenous injection site AEs, photosensitivity |
| Comparative cost consequences – non-medical costs *What is the impact of the intervention on non-medical costs such as disability costs, social services, lost productivity, caregiver time, etc.?*  ***Scoring scale:*** *-5: substantial additional expenditures (negative contribution)→5: substantial savings (positive contribution)*  *Possible sub-criteria:*   - *Impact on productivity* - *Financial impact on patients* - *Financial impact on caregivers* - *Costs to the wider social care system* | **Sources**:   - Peer-reviewed, published studies; economic evaluations - Unpublished data (*e.g.*, from health plan or manufacturer) - Data often derived from economic evaluations   **Content:** Capture the impact of providing coverage for the proposed intervention on non-medical costs   - Tabulate impact of intervention and comparators on non-medical costs, such as, disability, lost productivity, caregiver expenses as available. - Estimate total impact of intervention and comparator(s) and calculate the absolute difference (increment), if possible - If no savings with intervention, explain why; if intervention results in net savings, explain why (if not obvious from the table) - **Don’t** report data on cost of implementing intervention and comparators (*e.g.*, acquisition cost for a drug, equipment associated cost, etc.; covered under *Comparative cost consequences – cost of intervention*) - **Don’t** include potential for future cost increases for the intervention; this should be covered under *Comparative cost consequences – cost of intervention* - **Don’t** include data on other medical expenditures (covered in *Comparative cost consequences – other medical costs*) | **Study description**: indicate characteristics of the study from which the data presented is obtained including as applicable: Population; description of intervention (dose, duration) and comparators; model type, time horizon & key model features and assumptions including discounting; Costs included (except cost of intervention itself – see criteria above) (e.g., hospital costs, physician visits, adverse events, etc and how costs were obtained e.g. based on studies, expert opinion, assumptions); sensitivity analyses (on which data, based on which assumptions)  **Study results**  **Example**  **Impact of treatment Y on non-medical costs**  Includes patient productivity loss due to morbidity, patient productivity loss due to pre-mature mortality and paid caregiver costs based on data available in economic evaluation  **Mean costs (CAD$) per patient:** at 10 years   \|  \| **Patient produc-tivity loss due to morbidity** \| **Patient productivity loss due to pre-mature mortality** \| **Paid caregiver costs** \| \| --- \| --- \| --- \| --- \| \| **Treatment Y** \| 600 \| 5,000 \| 2 \| \| **Comp 1** \| 3,000 \| 4,500 \| 20 \| \| **Comp 2** \| 2,900 \| 5,500 \| 10 \| \| **No treatment** \| 300 \| 8,000 \| 1 \| |
| Knowledge about intervention | | |
| Quality of evidence *What is the quality of the design of studies and their relevance to the context?*  ***Scoring scale:*** *0: Not relevant and/or invalid →5: Highly relevant and valid*  *Possible sub-criteria:*   - *Validity* - *Relevance* - *Completeness of reporting* - *Type of evidence* | **Use instruments available under framework tools: *EVIDEM v4.0 Evidence matrix***.  **Sources**   - Quality assessment data is obtained by applying EVIDEM instruments to key studies for each type of evidence (usually includes clinical data, epidemiological data & economic data)   **Content:** Capture the overall quality of all types of evidence with respect to relevance and validity   - Report summary of quality assessment for each type of evidence - When several studies for one type of evidence (*e.g.*, clinical data), report the global summary covering all studies - Provide links to full quality assessments - **Don’t** report quality grades | \| **Type of evidence** \| **Quality assessment** \| \| --- \| --- \| \| **Clinical data** \| Overall, RCTs consistently demonstrate that GH treatment promotes height gain in girls with Turner Syndrome. Some uncertainty remains on extent to which GH may affect final height. High attrition rates were noted in the Canadian clinical trial; 2 multiphase trials were missing a control arm (no GH treatment) and chose GH administration mode (frequency of injections) that did not correspond to current practice. Safety data monitoring is generally limited, despite the numerous warnings and AEs associated with GH treatment in Turner Syndrome populations.  *See full assessment (Appendix)* \| \| **Economic evaluation** \| The relevance of one of the main outcomes chosen (cost per cm of final height) is questionable because it assigns the same value to each additional centimeter in height and precludes comparisons with other healthcare interventions. Other major issues are the exclusion of adverse events and the weakness of the utility data. A number of minor issues related to model design and assumptions are also present.  *See full assessment (Appendix)* \| \| **Epidemio-logical data** \| Etc. \| |
| Expert consensus / clinical practice guidelines *Is the intervention (or similar interventions) recommended in well-established guidelines? What type of recommendation (first line? Level 1?)*  ***Scoring scale:*** *0: Not recommended →5: Strong recommendation above all other alternatives* | **Sources**:   - Current clinical practice guidelines (CPGs) from professional specialists’ organizations, disease associations, HTA agencies; international as well as specific to the country / setting of interest (if available) - If CPGs not available, use recent peer-reviewed, review articles published in prominent journals or HTA reports to provide some expert recommendation   **Content**: Recommended treatment pathways and level of recommendation according to CPGs (country-specific and/or international), including place of intervention within the management of the health condition   - Report recommended treatment pathways for disease - Report position of CPGs regarding intervention or, if intervention-specific recommendations not yet available, similar types of intervention (*e.g.*, same therapeutic class) - Indicate recommended line of treatment and grade / strength of recommendation - Indicate which organization developed the guidelines, methodology, setting, participants, funding - **Don’t** include information on funding / reimbursement decisions for intervention; these are covered under *Political/historical/cultural context* | *Canadian Pain Society guidelines:*  *Chronic non cancer pain: mild to moderate: no mention of tramadol; 1st line non-opioid, 2nd line opioid (moderate to severe pain: switch to opioid earlier)*  *Chronic neuropathic pain: 1st line tricyclic antidepressants (TCA), gabapentin or pregabalin; 2nd line venlafaxine or duloxetine; 3rd line tramadol or conventional opioid*  *Limited information on the use of tramadol in Canadian guidelines was supplemented by guidelines from other countries recommending tramadol for:*  *Chronic low back pain: 2nd line, USA and Europe*  *Osteoarthritis: 2nd line, USA*  *Fibromyalgia: 2nd line, USA*  *Insert Table to facilitate comparison* |

| Criteria | **Background and Instructions** |
| --- | --- |
| CONTEXTUAL TOOL | |
| Normative contextual criteria | |
| Mandate and scope of healthcare system | **Background**  This criterion is included to ensure consideration whether the intervention is actually producing a good that is a health benefit falling under the mandate and within the scope of the health plan/healthcare system (it often implicitly does). In general terms, the goal of healthcare is to maintain normal functioning. Such consideration is aligned with the positions of utilitarianism (producing the “greatest benefits for the greatest number”) and beneficence.  **Sources**:   - Literature, disease association web sites, discussing such issues regarding the intervention or related interventions (e.g., should healthcare systems cover lifestyle interventions; is the intervention addressing a medical or social issue) - Information from health plan/system regarding explicitly defined mandate / scope (*e.g.*, a health plan may not consider lifestyle interventions to be part of its mandate). - Other specific information from health plan   **Content:** Provide information that can support the discussion on whether the proposed intervention is in line with the goal of the health plan/healthcare system and whether it does produce an actual health benefit. For example, enhancement of otherwise normal traits (*e.g.*, growth hormone for short stature) may not be considered a medical issue but rather a social issue, and thus not aligned with the mandate of a healthcare system/plan. One would question here whether such consideration impacts the value/appraisal of growth hormone for individuals with short stature. |
| Population priorities and access *Possible sub-criteria:*   - *Current priorities of health system strategic plan (e.g., disabled; low socioeconomic status; specific age groups)* - *Special populations (e.g., ethnicity)* - *Remote communities* - *Rare diseases* - *Specific therapeutic areas* | **Background**  This criterion is included to ensure consideration of priorities defined by decisionmakers or society. Such priorities may not be clearly defined but this criterion is meant to stimulate reflection on priorities and whether the intervention targets a group of patients that falls under prioritized populations. Priorities for specific groups of patients are defined by societies/decisionmakers and reflect their moral values. Such considerations are aligned with the principle of fairness, which considers treating like cases alike and different cases differently and often gives priority to those who are worst-off (theory of justice).  **Sources:**   - Literature - Laws & regulations for health plan/healthcare system outlining priorities, as available - Other specific information from health plan   **Content**: Provide information on:   - Priorities in relevant settings (*e.g.*, vulnerable populations, rare diseases, remote populations, prioritized therapeutic areas, etc.) - Characteristics of the intervention relevant to whether it is targeting a prioritized group. - Are patients afflicted by the condition considered worst off and thus should be prioritized under the realm of the “theory of justice”? Is the intervention associated with issues of access to care? Any discussion on whether the intervention triggers issues related to the concept of “treat like cases similarly”? - For example, interventions may be prioritized that target vulnerable populations and can be delivered in remote regions with limited facilities. |
| Common goal and specific interests *Possible sub-criteria:*   - *Stakeholder pressures* - *Stakeholder barriers* - *Conflict of interest* | **Background**  This criterion is included to prompt explicit consideration of pressures/barriers from stakeholders and awareness of the interests at stake, which are often part of the context surrounding healthcare decisionmaking. Awareness of stakeholder pressures / barriers helps ensure that decisions are fair-minded and driven by the common goal and not unduly influenced by specific interests (practical wisdom).  **Sources:**   - Literature, disease association websites, popular media - Other specific information from health plan   **Content**: Identify societal pressures and pressures from specific groups of stakeholders (clinicians, patients, industry, etc.), Example: pressures from specialists to use innovative technologies; pressures from patient groups for compassionate use. |
| Environmental impact *Possible sub-criteria:*   - *Environmental impact of production* - *Environmental impact of use* - *Environmental impact of implementation* | **Background**  This criterion is included to ensure explicit consideration of the environmental impact. The tool is used here to prompt identification of the potential environmental impact of the intervention from a qualitative standpoint. Its ethical implication is to protect the environment based on the ethical positions of utilitarianism and beneficence.  **Sources:**   - Literature   **Content**: Example: The intervention necessitates the use of radioactive isotopes which have a negative impact on the environment and require special containment. |
| Feasibility contextual criteria | |
| System capacity and appropriate use of intervention *Possible sub-criteria:*   - *Organizational requirements (e.g., process, premises, equipment)* - *Skill requirements* - *Legislative requirements* - *Surveillance requirements* - *Risk of inappropriate use* - *Institutional limitations to uptake* - *Ability to reach the whole target region/population* | **Background**  This criterion is included to ensure consideration of the capacity of the healthcare system to implement the intervention and to ensure its appropriate use in order to realize its potential benefit and avoid unintended consequences (practical wisdom). This depends on its infrastructure, organization, skills, legislation, barriers and risks of inappropriate use. Such considerations include mapping current systems and estimating whether the use of the intervention under scrutiny requires additional capacities (note: if economic estimate is available, it could be included in the economic criteria of the EVIDEM Core Model).  **Data sources**   - Literature - Specific information from health plan   **Content:** Provide information on:   - Risk of inappropriate use of intervention and its consequences - Implementation issues (infrastructure, organization, skills, legislation, barriers including human barriers) - Example: Ease of identifying patients who are most appropriate to receive the intervention - **Don’t** include economic estimates (costs) here. This should be covered under the economic criteria of the EVIDEM Core Model |
| Political / historical / cultural context *Possible sub-criteria:*   - *Political priorities and context* - *Cultural acceptability* - *Precedence (congruence with previous and future decisions)* - *Impact on innovation & research* - *Impact on partnership & collaboration among healthcare stakeholders* | **Background**  This criterion is included to ensure consideration of specific political/historical/cultural context that may impact the decision in consideration of political situations and overall priorities (*e.g.*, priority to innovation) as well as habits, traditions and precedence (practical wisdom).  **Sources:**   - Literature, disease association web site, popular media - Other specific information from health plan   **Content**: Provide information on:   - The political/historical/cultural context or precedence and traditions associated with the intervention or related interventions - Funding decisions for proposed intervention by other jurisdictions - Explicit governmental /political priorities (*e.g.*, innovation in healthcare, budget constraints in economic recession) beyond healthcare priorities (covered in *Opportunity costs and affordability*) - Example: *This type of intervention has historically always been considered in a similar fashion and is likely to be considered the same way (or for a new type of intervention, may set a precedent).* |
| OPPORTUNITY COST | |
| Opportunity costs and affordability *Possible sub-criteria:*   - *Opportunity costs for patient (foregone resources)* - *Opportunity costs for population (foregone resources)* - *Affordability* | **Background**  This criterion is included to ensure consideration of what may not be available anymore if using/reimbursing intervention (identifying foregone intervention in context of limited budget) and whether available resources are actually maximized (at patient and societal level) in doing so. This criterion also covers the concept of affordability. Both affordability and opportunity cost considerations require a financial/budgeting exercise, proposed below. It reflects the principle of efficiency (utilitarianism, practical wisdom).  **Sources and content:**  **Opportunity cost and affordability at patient level**: Discuss the role and cost of the intervention taking in account other types of interventions to manage/treat the condition (*e.g.*, psychological support for short stature rather than growth hormone treatment). Sources: Literature, disease association web sites, etc.  **Health plan level (cost at population level;** *comparative cost at patient level is covered under the cost criteria of the Core Model)* :   - **Opportunity costs:** after establishing the cost of the intervention for your population (see calculations below for affordability), explore with health plan which intervention could be disinvested if intervention under scrutiny is adopted/reimbursed ; - **Affordability**: Net impact of reimbursing the intervention on the budget of health plan (may include only intervention expenditures, but also other spending. Cost data on which these calculations are based should be those used in the Core Model (see Comparative cost criteria) Present methods and results of a relevant budget impact model:   - Indicate type of model (claim based, epidemiological), costs included (*e.g.*, dispensing fees, mark-up, cost of treatment administration, hospitalization, etc.) & major assumptions (number of patients, market share and switching from comparators a, b, etc., disease management patterns with intervention and comparators, etc.)   - Tabulate projected budget impact (number of patients, cost to health plan, incremental budget impact) as below:   **Projected annual impact of covering *intervention* on health plan/setting spending for *indication*** (includes intervention and implementation/administration and etc. costs, based on *origin* and *type* of budget impact model)  **Scenario : *Brief description of scenario including availability of comparators***   \|  \| **Number of patients receiving intervention (N_i_)** \| **Average annual cost per patient receiving intervention (C_i_)** \| **Annual intervention cost (T_i_ = N_i_ × C_i­_)** \| **Total annual cost if intervention is covered  (T_ic_=T_i_ +N_a,ic_ × C_a_ + N_b,ic_×C_b_ + …)** \| **Total annual cost if intervention is not covered  (T_inc_=N_a,inc_×C_a_ + N_b,inc_×C_b_ + …)** \| **Incremental (net) impact of intervention on spending for indication  (T_ic_ -T_inc_)** \| \| --- \| --- \| --- \| --- \| --- \| --- \| --- \| \| Year 1 \|  \|  \|  \|  \|  \|  \| \| Year 2 \|  \|  \|  \|  \|  \|  \| \| Year 3 \|  \|  \|  \|  \|  \|  \| \| Year 4 \|  \|  \|  \|  \|  \|  \| \| Year 5 \|  \|  \|  \|  \|  \|  \| \| **Total 5 years** \|  \|  \|  \|  \|  \|  \|   ic: intervention covered; inc: intervention not covered   - Provide explanation on difference between cost to health plan and incremental budget impact - Provide relevant data that may significantly affect estimate (*e.g.*, generics if not included in model) - Report results of sensitivity analyses   ***EXAMPLE:***  **Projected annual impact of covering the intervention on Canadian provincial / territorial drug plan spending for disease** (includes intervention and administration cost, based on health plans’ epidemiological model)  **Scenario: Year 5 after full reimbursement of intervention; intervention replaces comparators a and b**   \| **Annual number of patients receiving intervention per 100,000 population** \| **Average annual cost per patient receiving intervention** \| **Annual intervention cost of per 100,000 population** \| **Total annual cost per 100,000 population if intervention is covered** \| **Total annual cost per 100,000 population if intervention is not covered** \| **Incremental (net) impact of intervention on total spending for disease per 100,000 population** \| \| --- \| --- \| --- \| --- \| --- \| --- \| \| 150 \| $76,767 \| $11.5 million \| $21.7 million \| $20.3 million \| + $1.4 million \|   Assumptions:   - Prevalence of disease: 375 /100,000; 80% of patients are treated (i.e., 300 per 100,000) - Treatment mix in Year 5: If intervention not covered: comparator a: 20%, comparator b: 80%; If intervention covered: intervention: 50%, comparator a: 10%, comparator b: 40% - Mark-up, dispensing fees and discounts not included   The projected additional expenditures are due to replacement of lower-cost therapies (comparators a and b) with intervention.  Incremental budget impact sensitive to:   - Cost of comparator b (depends strongly on dose [U/kg], patient weight, and annual number of infusions): savings of $2.0 million if mean comparator b dose increases to 30 U/kg/week (actual dose observed in some studies) - If treatment rate increases to 90%: incremental net impact of intervention increases to $4.1 million |

# Reference list

List bibliography cited

Once completed, paste the list as an appendix in the ***EVIDEM v4.0 Adapt & pilot***.

# Evidence tables

- Evidence tables can be used to organized data from individual studies to facilitate the synthesis for the EVIDEM Evidence Matrix. The following template includes instructions and an example to facilitate the process.
- In the EVIDEM Evidence Matrix (see above), data is presented by outcome rather than by study, and data from the same cohort (but potentially presented in different publications) is presented only once in agreement with good HTA practices.

Note: Once completed, remove instructions and paste table as an appendix in the ***EVIDEM v4.0 Adapt & pilot***.

| **Study** | **Design** | **Population** | **Intervention** | **Outcomes** | **Main findings** |
| --- | --- | --- | --- | --- | --- |
| Name or citation,  country, CEBM level | Type of study, type of analysis setting, | Patients/disease state, N, attrition (drop out), n & age per cohort, inclusion and/or exclusion criteria | Treatments and dosages, duration, other interventions | Primary, secondary | Comparative table (outcome, intervention, comparators, *P*-value); data with stats (e.g., SD, IQR) |
| ***Example*** |  |  |  |  |  |
| John et al. 2011  **Netherlands**  **CEBM level: 2**  **(**hyperlink**)**  **Critical analysis (**hyperlink**)** | **Design**: RCT comparing GH treatment versus no treatment (control)  **Analyses**: per protocol  **Setting**: 20 Dutch hospital centers | **Patient**: infants and prepubertal children with PWS  **Enrolled: n** = 91  **Attrition:** 2 withdrawal due to adverse events IGF levels increased, spinal surgery, CPAP, psychotic events), 18 excluded from analyses (data not yet available at time of analyses)  **Population 1 - Infants,**  **n=** 42  **GH**: enrolled: n=20 (age 2.0 years); analyzed: n= 16 yr 1  **Control**: enrolled: n=22 (age 1.3 years); analyzed: n= 15 yr 1  **Population 2: Prepubertal children,**  **n=** 49  **GH**: enrolled: n=25 (age 6.8 years)  analyzed: n= 21 (yr 1), n= 20 (yr 2)  **Control**: enrolled: n=22 (age 5.9 years)  analyzed: n= 21 (yr 1), n= 20 (yr 2)  **Inclusion/exclusion criteria**: | **GH**; 1 mg/m2/day s.c.  **Control**: no treatment  **Treatment duration**  **Group 1:** 1 year (after yr1, infants were offered a year of GH treatment – not reported here as no control group)  **Group 2:** 2 years  **Other interventions**: | **Primary**  Lean body mass (DEXA Lunar Prodigy);  % fat (DEXA lunar Prodigy)  Head circumference (not reported here);  **Secondary**:  Etc. | **Infants**   \| **Outcome**  **(median [IQR])** \| **GH** \| **Control** \| ***P^a^*** \| \| --- \| --- \| --- \| --- \| \| **Height SDS** \|  \|  \|  \| \| Baseline \| -2.3 (-2.8 - -0.7) \| -2.1 (-3.2 - -1.0) \|  \| \| Year 1 \| -1.0 (-1.9 – 0.1) \| -1.8 (-3.5 - -1.4) \| <0.001 \| \| etc \|  \|  \|  \|   ***^a^***Change from baseline vs control  **Prebubertal children**   \| **Outcome**  **(median (IQR)** \| **GH** \| **Control** \| ***P*** \| \| --- \| --- \| --- \| --- \| \| **Fasting LDL (mmol/L)** \|  \|  \|  \| \| Baseline \|  \|  \|  \| \| Year 1 \|  \|  \|  \| \| Year 1 \|  \|  \|  \| \| etc \|  \|  \|  \| |

# Quality assessment tools

## Epidemiological data

### Tool for assessing the quality of single epidemiological study

| **Epidemiological data** | | | | | |
| --- | --- | --- | --- | --- | --- |
| **Instructions for assessment of quality of evidence**   - Perform a critical analysis of study(ies) to comment on all dimensions below, provide a critical overview and a quality grade - Circulate to reviewer and to an expert for validation of content and to reach a consensus on critical overview and quality grade | | | | | |
| **Study:**  **Disease:**  **Setting:** | | | | | |
|  | | **Questions** | **Critical overview** | | **Quality grade** |
| **Overall assessment of epidemiological data** | | Is the study question relevant (setting, condition of interest, outcome measures)?  Is the design appropriate (sample type and size, data sources and measurements, analyses, statistics)?  *See dimensions below* |  | | 1  Low  2  Moderate  3  High  4  Excellent |
|  | **Dimension** | **Questions** | | **Comment** | |
| **1** | **Study population** | Is the study population (sample) representative of the target population (usually the general population) for which prevalence or incidence estimates are sought?  Were the sampling method (e.g., random sampling) and the sampling frame (*e.g.*, registry, insurance claims database) to select the study population valid? Were valid methods used to assess and address sampling bias?  Are the items above fully reported? | |  | |
| **2** | **Setting and period of data collection** | Are the setting, including locations, clinical setting and period of data collection, reported? Are they relevant and valid? | |  | |
| **3** | **Condition of interest** | Is the condition, for which prevalence or incidence estimates are reported, clearly defined, including case definition, diagnostic criteria and codes, disease stage, age of onset, duration, frequency of symptoms, comorbidities?  Is the condition, for which prevalence or incidence estimates are reported (as defined above), relevant and valid? Does it correspond to the indication in which the intervention is envisioned to be used? | |  | |
| **4** | **Outcome measures** | Are the reported outcome measures (*e.g.*, prevalence at birth, point prevalence, incidence density, cumulative incidence) clearly defined? Are they relevant with respect to the condition of interest? | |  | |
| **5** | **Data sources/ measurements** | Are the methods used to establish the presence/absence of the condition fully reported? Are these methods valid (*e.g.*, validity of diagnostic test, frequency of examination [for incidence studies])?  Are the sources used to establish the occurrence of the condition (*e.g.*, outcomes measured specifically for the study, medical records, insurance claims databases, registries, self-report) reported? Are they valid? (All diagnosable cases in study sample identified? Any misclassification prevented?) | |  | |
| **6** | **Study sample size** | Was the study sample size sufficient to arrive at reliable epidemiological estimates, including estimates for population subgroups? | |  | |
| **7** | **Analyses** | Are the analyses performed clearly described? Are the analyses appropriate and comprehensive with sound and relevant statistics (*e.g.*, missing data; control of confounding; population subgroup analyses)? | |  | |
| **8** | **Conclusion** | Are the conclusions supported by the results? | |  | |

### Overall assessment tool for epidemiological studies

| **SUMMARY**  **Epidemiological studies** | | | |
| --- | --- | --- | --- |
| **Disease:**  **Setting:** | | **Series of studies**   - List individual studies included; a detailed assessment using instrument above should be available for each study prior to summarizing for the series of studies for a given type of evidence | |
| **Type of evidence** | **Questions** | **Critical overview** | **Quality grade** |
| **Epidemiological data** | How relevant is the research program with regard to epidemiological data? Are conclusions valid across the range of studies (conclusions across studies consistent or conflicting)?  Are individual trials relevant and valid? COMPLETE THE INSTRUMENT ABOVE FOR EACH INDIVIDUAL STUDY |  | 1  Low  2  Moderate  3  High  4  Excellent |

## Efficacy, effectiveness and patient-reported outcomes data

### Tool for assessing the quality of a single interventional study

| **INTERVENTIONAL STUDY Efficacy, effectiveness, safety, and patient-reported outcomes data** | | | | | |
| --- | --- | --- | --- | --- | --- |
| **Instructions for assessment of quality of evidence**   - Perform a critical analysis of study(ies) to comment on all dimensions below, provide a critical overview and a quality grade - Circulate to reviewer and to an expert for validation of content and to reach a consensus on critical overview and quality grade | | | | | |
| **Study:**  **Disease:**  **Intervention:**  **Setting:** | | | | | |
|  | | **Questions** | **Critical overview** | | **Quality grade** |
| **Overall assessment of efficacy / effectiveness, safety and patient-reported outcomes data** | | Is the study question relevant (choice of comparator, time horizon, patient population, and outcome measures)?  Is the design appropriate with a low risk of bias (setting & design, sample size, patient allocation, analyses, statistics)?  *See dimensions below* |  | | 1  Low  2  Moderate  3  High  4  Excellent |
|  | **Dimension** | **Questions** | | **Comment** | |
| **1** | **Target population** | Are population characteristics fully reported? Is the setting clearly described?  Is the target population relevant (age, gender, disease stage, comorbidities, inclusion criteria/exclusion criteria, setting, etc.)? Does it correspond to the actual population in which the intervention is envisioned to be used? | |  | |
| **2** | **Intervention & comparators** | Is the intervention in agreement with expected use? Does the choice of comparators reflect standard of care? | |  | |
| **3** | **Outcome measures** | Are all outcomes measures well defined (efficacy, safety and PRO)? Are the selected outcome measures (efficacy, safety and PRO) relevant? Are rationales for outcomes selection reported? Are they valid?  Are the instruments/methods/units used to measure outcomes reported? Are the instruments/methods/units used to measure outcomes (efficacy, safety, PRO) valid? | |  | |
| **4** | **Study design** | Was the sample size appropriate?  Is the design fully detailed? Is the design appropriate? For RCTs, this includes: randomization; patient allocation concealment; blinding as appropriate; completeness of follow-up; avoidance of carry-over effects.  Were the groups similar at the outset of the study in terms of prognostic factors (*e.g.*, severity of disease)? | |  | |
| **5** | **Adverse events** | Are methods for monitoring the occurrence of adverse events and assumptions with regard to which adverse events are related to the intervention(s) relevant and valid in light of previous experience with similar interventions/populations?  Are all relevant adverse events reported (deaths, serious, common, discontinuations), both treatment-related and all-cause? | |  | |
| **6** | **Time horizon** | Is time horizon long enough to capture all meaningful differences in key outcomes between the intervention and comparators? (*e.g.*, Was the study stopped prematurely because of observed benefits or harms?) | |  | |
| **7** | **Analyses** | Are the analyses clearly described?  Are the analyses appropriate and comprehensive with sound and relevant statistics (*e.g.*, intention-to-treat; missing data; control of confounding; subgroup analyses)? | |  | |
| **8** | **Results (precision and strength of effect)** | Are results clearly reported, including patient disposition (population flow)?  Are differences observed meaningful (clinically and statistically)? Is there evidence of a dose-response relationship? | |  | |
| **9** | **Conclusion** | Are the conclusions supported by the results? | |  | |

### Tool for assessing the quality of a single observational study

| **OBERVATIONAL STUDY: Efficacy, effectiveness, safety, and patient-reported outcomes data** | | | | | |
| --- | --- | --- | --- | --- | --- |
| **Instructions for assessment of quality of evidence**   - Perform a critical analysis of study(ies) to comment on all dimensions below, provide a critical overview and a quality grade - Circulate to reviewer and to an expert for validation of content and to reach a consensus on critical overview and quality grade | | | | | |
| **Study:**  **Disease:**  **Intervention:**  **Setting**: | | | | | |
|  | | **Questions** | **Critical overview** | | **Quality grade** |
| **Overall assessment of efficacy / effectiveness, safety and patient-reported outcomes data** | | Is the study question relevant (choice of comparator, time horizon, patient population, and outcome measures)?  Is the design appropriate with a low risk of bias (setting & design, sample size, patient allocation, analyses, statistics)?  *See dimensions below* |  | | 1  Low  2  Moderate  3  High  4  Excellent |
|  | **Dimension** | **Questions** | | **Comment** | |
| **1** | **Population** | Are the population characteristics fully reported? Is the setting clearly described?  Is the study population relevant (age, gender, disease stage, comorbidities, inclusion criteria/exclusion criteria, setting, etc.)? Does it correspond to the actual population in which the intervention is envisioned to be used? | |  | |
| **2** | **Exposures** | Are the exposures investigated relevant with respect to the intervention being assessed, in terms of type, dose and duration? | |  | |
| **3** | **Outcome measures** | Are all outcomes measures well defined (efficacy, safety and PRO)?  Are the selected outcome measures (efficacy, safety and PRO) relevant?  Are assumptions for outcomes selection reported? Are rationales for outcome selection valid?  Are the instruments/methods/units used to measure outcomes reported? Are the instruments/methods/units used to measure outcomes (*e.g.*, database analysis, patient interviews, surveys, etc.) valid? | |  | |
| **4** | **Study design** | Was the sample size appropriate?  Is the design fully detailed? Was the design appropriate? This includes: avoidance of carry-over effects; completeness of follow-up; appropriate matching algorithm (others?). Were the groups similar (*i.e.*, well matched) in terms of prognostic factors (*e.g.*, severity of disease)? | |  | |
| **5** | **Time horizon** | Is the period of data collection clearly described?  For cohort studies, was the time horizon adequate to capture all meaningful differences in key outcomes between the exposed and un-exposed? For cross-sectional and case-control studies, was the study carried out recently? | |  | |
| **6** | **Analyses** | Are the analyses clearly described?  Are the analyses appropriate and comprehensive with sound and relevant statistics (*e.g.*, adherence to intention-to-treat principle; handling of missing data; control of confounding; subgroup analyses)? | |  | |
| **7** | **Results (precision and strength of effect)** | Are results clearly reported, including patient disposition (population flow)?  Are differences observed meaningful (clinically and statistically)? Is there evidence of a dose-response relationship? | |  | |
| **8** | **Conclusion** | Are the conclusions supported by the results? | |  | |

### Tool for assessing the quality of an intervention outcomes modelling study

| **INTERVENTION OUTCOMES MODELING STUDY: Efficacy, effectiveness, safety, and patient-reported outcomes data** | | | | | | |
| --- | --- | --- | --- | --- | --- | --- |
| **Instructions for assessment of quality of evidence**   - Perform a critical analysis of study(ies) to comment on all dimensions below, provide a critical overview and a quality grade - Circulate to reviewer and to an expert for validation of content and to reach a consensus on critical overview and quality grade | | | | | | |
| **Disease:**  **Intervention:**  **Setting:** | | | | **Study:** | | |
|  | | **Questions** | **Critical overview** | | | **Quality grade** |
| **Overall assessment of outcomes models** | | Is the study question relevant (choice of comparator, time horizon, patient population, outcomes)?  Is the design appropriate (*i.e.*, how close to real disease progression, strength of assumptions, sensitivity analyses, quality of sources [clinical and natural history])?  *See dimensions below* |  | | | 1  Low  2  Moderate  3  High  4  Excellent |
|  | **Dimension** | **Questions** | | | **Comment** | |
| **1** | **Target population** | Is the target population fully defined?  Is the target population relevant (age, gender, disease stage, co-morbidities, etc.)? Is it comparable to the trial/study population in which efficacy/effectiveness data was obtained? Does it correspond to the actual population in which the treatment is envisioned to be used? | | |  | |
| **2** | **Intervention and setting** | Are the interventions (comparators) fully described? Is the setting reported?  Are assumptions/design regarding interventions (dose & duration, mode of delivery) & setting (hospital/community, country, etc.) reported? Are these valid with respect to the indication/proposed coverage & clinical context? | | |  | |
| **3** | **Comparators** | Does the choice of comparators reflect current practice? | | |  | |
| **4** | **Outcome measures** | Are the selected outcome measures (efficacy, safety and patient reported outcomes [PRO]) relevant? Are the primary efficacy/effectiveness measures used, and major side effects included? Are instruments to estimate PROs valid? Are assumptions for outcomes selection valid? | | |  | |
| **5** | **Time horizon** | Is the time horizon reported? Is the time horizon long enough to capture all meaningful differences in outcomes between the intervention and comparators?  Are all assumptions made reported? Are they valid? | | |  | |
| **6** | **Clinical event pathway** | Is the event pathway fully reported? Does the model reflect a realistic event pathway according to current knowledge? | | |  | |
| **7** | **Parameters** | Are all parameters used in the model/study (effectiveness data, adverse event probabilities, health states, PROs) reported?  Are the sources and methods used to estimate the parameters reported (effectiveness data, adverse event probabilities, health states, PROs)? Are they solid?  Are all assumptions made reported? Are they valid? | | |  | |
| **8** | **Sensitivity analyses** | Do sensitivity analyses cover the most critical parameters within a valid range? | | |  | |
| **9** | **Results** | Are disaggregated results reported? Are sensitivity analyses fully reported? | | |  | |
| **10** | **Conclusion** | Are conclusions supported by results? | | |  | |

### Overall assessment tool for efficacy, effectiveness and patient-reported outcomes studies

| **SUMMARY**  **Efficacy, effectiveness and patient-reported outcomes studies (interventional, observational, modeling)** | | | |
| --- | --- | --- | --- |
| **Disease:**  **Intervention:**  **Setting**: | | **Series of studies**   - List individual studies included; a detailed assessment using instruments above should be available for each study prior to summarizing for the series of studies for a given type of evidence | |
| **Type of evidence** | **Questions** | **Critical overview** | **Quality grade** |
| **Overall assessment of efficacy / effectiveness, safety and patient-reported outcomes data** | How relevant is the research program with regard to interventional and observational data? Are conclusions valid over the range of studies (conclusions across studies consistent or conflicting)?  Are individual trials relevant and valid? COMPLETE INSTRUMENT ABOVE FOR EACH INDIVIDUAL STUDY |  | 1  Low  2  Moderate  3  High  4  Excellent |

## Economic evaluations data

### Tool for assessing the quality of cost-consequence modelling studies

| **Economic modelling study – Cost-consequence** | | | | | | |
| --- | --- | --- | --- | --- | --- | --- |
| **Instructions for assessment of quality of evidence**   - Perform a critical analysis of study(ies) to comment on all dimensions below, provide a critical overview and a quality grade - Circulate to reviewer and to an expert for validation of content and to reach a consensus on critical overview and quality grade | | | | | | |
| **Disease:**  **Intervention:**  **Setting**: | | | | **Study:** | | |
|  | | **Questions** | **Critical overview** | | | **Quality grade** |
| **Overall assessment of economic models** | | Is the study question relevant (choice of comparator, time horizon, patient population, types of costs included)?  Is the design appropriate (*i.e.*, how close to real disease progression, strength of assumptions, intervention use projections [if performed], sensitivity analyses, quality of sources [unit costs, resource use])?  *See dimensions below* |  | | | 1  Low  2  Moderate  3  High  4  Excellent |
|  | **Dimension** | **Questions** | | | **Comment** | |
| **1** | **Target population** | Is the target population fully defined?  Is the target population relevant (age, gender, disease stage, co-morbidities, etc.)? Is it comparable to the trial/study population in which efficacy/effectiveness data was obtained? Does it correspond to the actual population in which the treatment is envisioned to be used? | | |  | |
| **2** | **Intervention and setting** | Are the interventions (comparators) fully described? Is the setting reported?  Are assumptions/design regarding interventions (dose & duration, mode of delivery) & setting (hospital/community, country, etc.) reported? Are they valid with respect to the indication/proposed coverage & clinical context? | | |  | |
| **3** | **Comparators** | Does the choice of comparators reflect current practice? For drugs, are generics included? | | |  | |
| **4** | **Perspective and costs included** | Is the perspective of the analysis reported? Are all costs relevant to the chosen perspective included (*e.g.*, cost of intervention [for drugs, dispensing fees], healthcare professional visits & procedures, hospitalization, long-term care, lab tests)? Are assumptions for cost inclusion/ exclusion valid? | | |  | |
| **5** | **Time horizon** | Is the time horizon reported? Is the time horizon long enough to capture all meaningful differences in costs between the intervention and comparators? Are assumptions used valid? | | |  | |
| **6** | **Clinical event pathway** | Is the event pathway fully reported? Does the model reflect a realistic event pathway according to current knowledge? | | |  | |
| **7** | **Parameters estimates** | Are all parameters used in the model/study (resource use, unit costs, clinical event probabilities) reported?  Are the sources and methods used to estimate the model parameters valid (resource use, unit costs, claims, number of patients, clinical event probabilities)? Are assumptions and sources used valid? | | |  | |
| **8** | **Discount rate** | Is the recommended discount rate applied? | | |  | |
| **9** | **Sensitivity analyses** | Do sensitivity analyses cover the most critical parameters within a valid range? | | |  | |
| **10** | **Results** | Are disaggregated results reported? Are sensitivity analyses fully reported? | | |  | |
| **11** | **Conclusion** | Are conclusions supported by results? | | |  | |

### Tool for assessing the quality of cost-effectiveness modelling studies

| **Economic modelling study – Cost-Effectiveness** | | | | | | |
| --- | --- | --- | --- | --- | --- | --- |
| **Instructions for assessment of quality of evidence**   - Perform a critical analysis of study(ies) to comment on all dimensions below, provide a critical overview and a quality grade - Circulate to reviewer and to an expert for validation of content and to reach a consensus on critical overview and quality grade | | | | | | |
| **Disease:**  **Intervention:**  **Setting**: | | | | **Study:** | | |
|  | | **Questions** | **Critical overview** | | | **Quality grade** |
| **Overall assessment of economic evaluation** | | Is the study question relevant (choice of comparator, time horizon, patient population, outcome, perspective)?  Is the design appropriate (*i.e.*, how close to real disease progression, costs included, strength of assumptions, sensitivity analyses, quality of sources [clinical, costs, epidemiology, and utilities])?  *See dimensions below* |  | | | 1  Low  2  Moderate  3  High  4  Excellent |
|  | **Dimension** | **Questions** | | | **Comment** | |
| **1** | **Target population** | Is the target population fully defined?  Is the target population relevant (age, gender, disease stage, co-morbidities, etc.)? Is it comparable to the trial/study population in which efficacy/effectiveness data was obtained? Does it correspond to the actual population in which the treatment is envisioned to be used? | | |  | |
| **2** | **Intervention and setting** | Are the interventions (comparators) fully described? Is the setting reported?  Are assumptions/design regarding interventions (dose & duration, mode of delivery) & setting (hospital/community, country, etc.) reported? Are they valid with respect to the indication/proposed coverage & clinical context? | | |  | |
| **3** | **Comparators** | Does the choice of comparators reflect current practice? For drugs, are generics included? | | |  | |
| **4** | **Perspective and costs included** | Is the perspective of the analysis reported? Are all costs relevant to the chosen perspective included (*e.g.*, cost of intervention [for drugs, dispensing fees], healthcare professional visits & procedures, hospitalization, long-term care, lab tests)? Are assumptions for cost inclusion/ exclusion valid? | | |  | |
| **5** | **Time horizon** | Is the time horizon reported? Is the time horizon long enough to capture all meaningful differences in costs and outcomes between the intervention and comparator? Are assumptions used valid? | | |  | |
| **6** | **Clinical event pathway** | Is the event pathway fully reported? Does the model reflect a realistic event pathway according to current knowledge? | | |  | |
| **7** | **Parameter estimates** | Are all parameters used in the model/study (resource use, unit costs, clinical event probabilities, utilities) reported?  Are the sources and methods used to estimate the model parameters valid (resource use, unit costs, claims, number of patients, clinical event probabilities)? Are assumptions and sources used valid? | | |  | |
| **8** | **Outcome measures** | Are the selected outcome measures (efficacy, safety and patient-reported outcomes [PRO]) relevant? Are the primary efficacy/effectiveness measures used, and major side effects included? Are instruments to estimate PRO valid? Are assumptions for outcomes selection valid? | | |  | |
| **9** | **Discount rate** | Is the recommended discount rate applied? | | |  | |
| **10** | **Sensitivity analyses** | Do sensitivity analyses cover the most critical parameters within a valid range? | | |  | |
| **11** | **Results** | Are disaggregated results reported? Are sensitivity analyses fully reported? | | |  | |
| **12** | **Conclusion** | Are conclusions supported by results? | | |  | |

### Tool for assessing the quality of budget impact model studies

| **Budget impact model** | | | | | | |
| --- | --- | --- | --- | --- | --- | --- |
| **Instructions for assessment of quality of evidence**   - Perform a critical analysis of study(ies) to comment on all dimensions below, provide a critical overview and a quality grade - Circulate to reviewer and to an expert for validation of content and to reach a consensus on critical overview and quality grade | | | | | | |
| **Disease:**  **Intervention:**  **Setting**: | | | | **Study:** | | |
|  | | **Questions** | **Critical overview** | | | **Quality grade** |
| **Overall assessment of budget impact model** | | Is the study question relevant (perspective, choice of comparators, time horizon, and patient population)?  Is the design appropriate (how close to real market evolution, costs included [dispensing fees and wholesaler mark-up], strength of assumptions, sensitivity analyses, quality of sources [clinical, costs, epidemiology])?  Are generics included?  *See dimensions below* |  | | | 1  Low  2  Moderate  3  High  4  Excellent |
|  | **Dimension** | **Question** | | | **Comment** | |
| **1** | Target population | Does the target population correspond to the expected use/target coverage (age, disease stage, comorbidities, first line, second line after failure with XX, etc.)? | | |  | |
| **2** | Intervention and setting | Are assumptions/design regarding interventions (treatment dose, treatment duration, mode of delivery) valid with respect to indication/proposed coverage & clinical context? | | |  | |
| **3** | Model & projections | Is the model structure valid? Are rationales for projections (new patients, switching between interventions, market movement & future introduction of other interventions) based on evidence? Is it realistic according to current knowledge? Is time horizon long enough? | | |  | |
| **4** | Input data | Are input data, their sources and methods for estimates valid (claims, patients, epidemiology, unit costs for intervention, clinical data)? For drugs, are generics included? | | |  | |
| **5** | Cost of intervention | Are all relevant costs of the intervention considered (*e.g.*, for drugs, dispensing fees)? Are assumptions for cost estimates valid? | | |  | |
| **6** | Sensitivity analyses | Do sensitivity analyses cover the most critical parameters within a valid range? | | |  | |
| **7** | Conclusion | Are conclusions supported by results? | | |  | |
| **8** | Transparency of reporting | Are the target population, event pathway and interventions fully reported? Is the design/model fully described? Are all the parameters used in the model/study and their sources/methods to obtain reported? Are all assumptions listed? Is the design fully detailed? Are the analyses clearly described and disaggregated results and sensitivity analyses fully reported? | | |  | |
